# Bismuth(III)-catalyzed three-component selenation of imidazo[1,2-*a*]pyridines or related arenes with selenium and arylboronic acids

**DOI:** 10.3762/bjoc.22.96

**Published:** 2026-08-28

**Authors:** Naoki Aiba, Mio Matsumura, Yuki Tada, Yuki Murata, Shuji Yasuike

**Affiliations:** 1 School of Pharmaceutical Sciences, Aichi Gakuin University, 1-100 Kusumoto-cho, Chikusa-ku, Nagoya 464-8650, Japan

**Keywords:** arylboronic acid, bismuth catalyst, 3-selanylimidazopyridine, selenium, three-component reaction

## Abstract

Unsymmetrical diaryl selenides bearing heteroaromatic moieties have attracted increasing attention because of their notable biological activities. In this paper, a novel bismuth-catalyzed three-component reaction is reported for the synthesis of 3-selanylimidazopyridines from imidazopyridines, arylboronic acids, and elemental selenium. The reaction proceeded under aerobic conditions at 100 °C using BiI_3_ (10 mol %) as the catalyst, affording the desired products in moderate to good yields. The application of this method to other electron-rich arenes was also explored. This transformation represents the first example of a three-component reaction catalyzed by a bismuth reagent. The use of BiI_3_, a non-hygroscopic and low-toxicity catalyst, offers an alternative to traditional transition-metal-catalyzed systems.

## Introduction

**Scheme 1:**
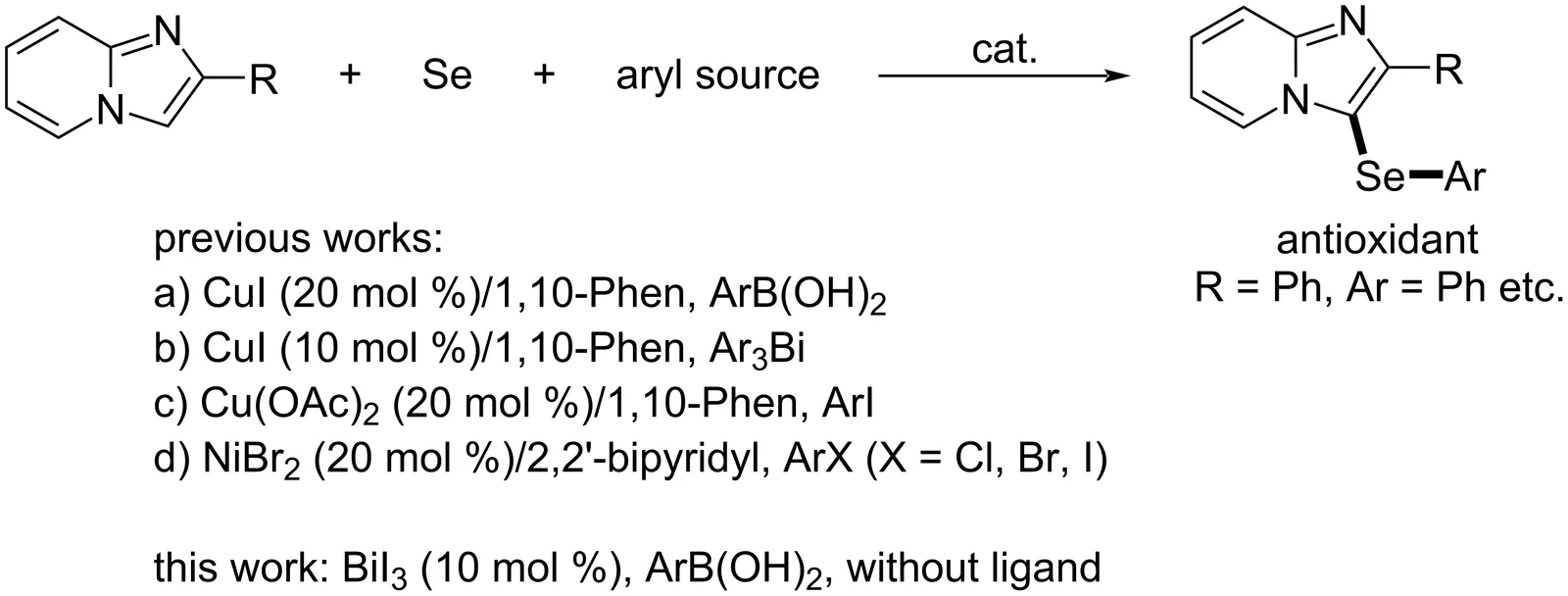
Syntheses of 3-(arylselanyl)imidazopyridines using three-component reactions.

Efficient and sustainable strategies for C–Se bond formation [1–8] are crucial in organic synthesis, as they enable the production of pharmaceuticals and bioactive compounds that incorporate selenium atoms [7–12]. For example, one-pot double selenation offers a straightforward method for synthesizing unsymmetrical diaryl selenides via a transition-metal-catalyzed three-component reaction using cost-effective and stable selenium powder as the selenium source [2,13–14]. This approach has attracted considerable interest as a powerful but demanding pathway for assembling unsymmetrical selenides, allowing two distinct functional aryl groups to be attached to a single selenium atom simultaneously. 3-(Arylselanyl)imidazopyridines have also garnered attention due to their interesting biological activities, which include antioxidant effects [15]. Consequently, a number of synthetic methods based on three-component reactions of various aryl sources (e.g., arylboronic acids, triarylbismuthines, or aryl halides) with imidazopyridines and Se powder in the presence of an appropriate transition-metal catalyst (e.g., CuI, Cu(OAc)_2_, or NiBr_2_) and ligand (e.g., 1,10-phenanthroline or 2,2'-bipyridyl) have been reported (Scheme 1a–d) [16–19]. However, due to toxicity concerns related to the transition-metal catalysts, the development of synthetic methods with a reduced environmental impact is desirable.

Bismuth is located in the 6th period of the 15th group of the periodic table and is a heavy main group element. Inorganic compounds containing bismuth have gained attention in organic synthesis because of their excellent reactivities as mild Lewis acids, in addition to their low toxicities and environmental friendliness [20–25]. For example, BiCl_3_ has been used as a catalyst for a wide range of transformations. These include the Mukaiyama aldol reactions [26–27], the nucleophilic ring-opening reactions of epoxides [28], deoxygenative allylation [29], and Diels–Alder reactions [30–31]. It has also been applied to multicomponent reactions involving aldehydes with amines and alkynes or ketones for the synthesis of propargylamines or β-aminocarbonyl compounds [32–33]. Moreover, BiCl_3_ has demonstrated catalytic activity in Friedel–Crafts reactions [34], oxa-Michael addition reactions [35], the aminooxygenation of propargylamidines [36], and in the tandem cyclization of tryptamine-ynamides [37]. Recently, BiCl_3_ has garnered interest as an alternative to transition-metal catalysts in organic synthesis. Chakraborty et al. demonstrated its utility in the Suzuki reaction, which involves C(Ar)–C(Ar) bond formation [38]. Additionally, Swu and Aamir Bin Riyaz reported the effectiveness of BiCl_3_ in promoting the reaction of benzimidazol-2-amines with arylboronic acids, leading to the formation of *N*-arylbenzimidazol-2-amines via C(Ar)–N bond formation [39]. In contrast, BiI_3_ is primarily employed in semiconductor and solar cell development [40–41]; however, its utility as a catalyst in organic synthesis remains largely underexplored. Furthermore, the reported uses of catalytic BiI_3_ have been limited to a few reactions, such as acetal deprotection, guanylation via the desulfurization of thioureas with amines, and the *S,S*-acetalization of benzaldehyde [42–44]. Recently, our research group reported that the reaction of indoles with diaryl diselenides in the presence of 10 mol % BiI_3_ yielded 3-selanylindoles via a regioselective C–H selenation at the 3-position of the indole [45]. Thus, focusing on the expanding applicability of BiI_3_, this paper presents a novel bismuth(III)-catalyzed three-component reaction of Se powder with imidazo[1,2-*a*]pyridines and arylboronic acids for the synthesis of 3-(arylselanyl)imidazopyridines (Scheme 1, this work) and its application to other electron-rich arenes.

## Results and Discussion

Initially, the optimal conditions were investigated for the synthesis of 3-(phenylselanyl)imidazopyridine **9aa** via a three-component reaction involving 2-phenylimidazo[1,2-*a*]pyridine (**1a**), Se, and aryl reagents **2a**–**8**. The results obtained during the screening of various catalysts, solvents, aryl donors, and reagent ratios are summarized in Table 1. Initially, **1a** (0.5 mmol) was reacted with Se (0.5 mmol) and various aryl reagents **2a**‒**8** in the presence of 10 mol % of BiI_3_ under aerobic conditions in dimethylformamide (DMF) at 100 °C for 24 h (Table 1, entries 1–7). The reactions employing phenylboronic acid (**2a**) and triphenylbismuthine (**7**) afforded the corresponding compound **9aa** in moderate yields of 56% and 44%, respectively (Table 1, entries 1 and 6). In contrast, other boron reagents **3** and **4**, silicon and tin reagents **5** and **6** as well as iodobenzene (**8**) proved less effective in producing the desired product. Moreover, despite the fact that triphenylbismuthine (**7**) contains three phenyl groups, it produced only a moderate product yield. Therefore, phenylboronic acid (**2a**) was selected as the phenyl-group donor and several commercially available Lewis acids were screened to evaluate their suitability as catalyst in the reaction between **1a** and **2a** (Table 1, entries 1 and 8–16). As indicated, only BiI_3_ was found to catalyze the reaction, with minimal reaction progress being observed for the other Lewis acids. A subsequent solvent screening revealed that the reaction proceeded efficiently in DMF (56%), whereas *N*-methyl-2-pyrrolidone (NMP) and dimethyl sulfoxide (DMSO) were less efficient and toluene, 1,4-dioxane, 1,2-dichloroethane, and ethanol failed to yield the desired product (Table 1, entries 1, 17–22). Next, the optimum amounts of Se powder and phenylboronic acid (**2a**) for addition to **1a** were also investigated (Table 1, entries 1, 23, and 24). Specifically, the reaction of **1a** with Se powder (1.2 equiv) and boronic acid **2a** (1.2 equiv) proved to be superior, affording product **9aa** in a good yield (72%, Table 1, entry 23). With all other parameters kept unchanged, no improvements in the product yield were observed upon increasing the reaction temperature to 130 °C or increasing the BiI_3_ catalyst loading to 20 mol % (Table 1, entries 25 and 26). Additionally, performing the reaction under oxygen produced **9aa** in a 71% yield, which was almost the same as that obtained under aerobic conditions. In contrast, the reaction was completely suppressed under an argon atmosphere (0%, Table 1, entry 28). Notably, the optimal results were obtained under aerobic conditions, offering practical advantages in terms of the operational simplicity (Table 1, entry 23).

**Table 1:** Screening of reaction conditions.^a^

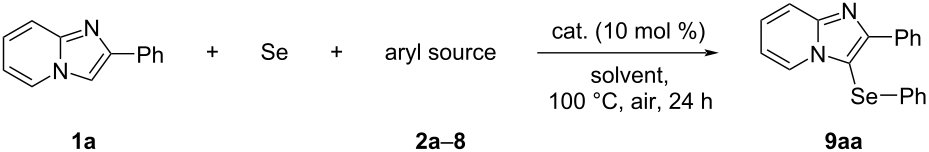					

Entry	Aryl source	Ratio **1a**:Se:**2a**–**8**	Cat.	Solvent	Yield (%)^b^

1	PhB(OH)_2_, **2a**	1:1:1	BiI_3_	DMF	56
2	PhBpin, **3**	1:1:1	BiI_3_	DMF	0
3	PhBF_3_K, **4**	1:1:1	BiI_3_	DMF	0
4	PhSi(OEt)_3,_ **5**	1:1:1	BiI_3_	DMF	0
5	PhSn(*n-*Bu)_3_, **6**	1:1:1	BiI_3_	DMF	15
6	Ph_3_Bi, **7**	1:1:1	BiI_3_	DMF	44
7	PhI, **8**	1:1:1	BiI_3_	DMF	0
8	PhB(OH)_2_, **2a**	1:1:1	BiBr_3_	DMF	19
9	PhB(OH)_2_, **2a**	1:1:1	BiCl_3_	DMF	0
10	PhB(OH)_2_, **2a**	1:1:1	BiF_3_	DMF	0
11	PhB(OH)_2_, **2a**	1:1:1	Bi(OTf)_3_	DMF	3
12	PhB(OH)_2_, **2a**	1:1:1	SbBr_3_	DMF	0
13	PhB(OH)_2_, **2a**	1:1:1	SbI_3_	DMF	7
14	PhB(OH)_2_, **2a**	1:1:1	AlCl_3_	DMF	0
15	PhB(OH)_2_, **2a**	1:1:1	FeCl_3_	DMF	0
16	PhB(OH)_2_, **2a**	1:1:1	InCl_3_	DMF	0
17	PhB(OH)_2_, **2a**	1:1:1	BiI_3_	NMP	46
18	PhB(OH)_2_, **2a**	1:1:1	BiI_3_	DMSO	38
19	PhB(OH)_2_, **2a**	1:1:1	BiI_3_	toluene	0
20	PhB(OH)_2_, **2a**	1:1:1	BiI_3_	1,4-dioxane	0
21^c^	PhB(OH)_2_, **2a**	1:1:1	BiI_3_	1,2-DCE	0
22^c^	PhB(OH)_2_, **2a**	1:1:1	BiI_3_	EtOH	0
23	PhB(OH)_2_, **2a**	1:1.2:1.2	BiI_3_	DMF	72
24	PhB(OH)_2_, **2a**	1:1.5:1.5	BiI_3_	DMF	70
25^d^	PhB(OH)_2_, **2a**	1:1.2:1.2	BiI_3_	DMF	70
26^e^	PhB(OH)_2_, **2a**	1:1.2:1.2	BiI_3_	DMF	68
27^f^	PhB(OH)_2_, **2a**	1:1.2:1.2	BiI_3_	DMF	71
28^g^	PhB(OH)_2_, **2a**	1:1.2:1.2	BiI_3_	DMF	0

^a^Conditions: **1a** (0.5 mmol), Se (0.5–0.75 mmol), aryl source **2**–**8** (0.5–0.75 mmol), solvent (2 mL); ^b^yield of isolated product; ^c^at 90 °C; ^d^at 130 °C; ^e^BiI_3_ (20 mol %); ^f^under O_2_; ^g^under Ar.

**Scheme 2:**
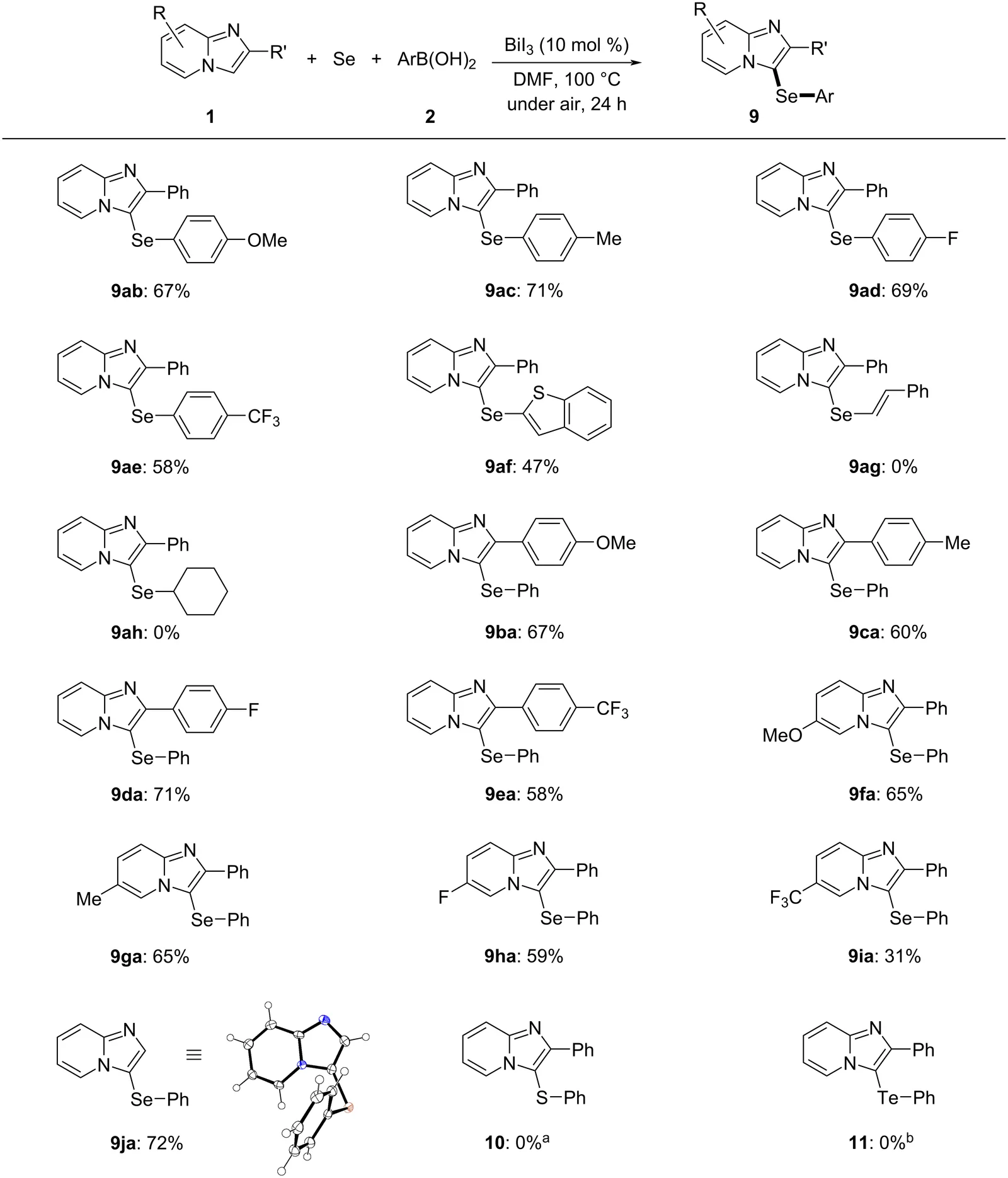
Synthesis of 3-(arylselanyl)imidazopyridines via three-component reaction of Se powder with various imidazopyridines **1** and boronic acids **2**. Conditions: imidazopyridines **1** (0.5 mmol), Se (0.6 mmol), arylboronic acids **2** (0.6 mmol), BiI_3_ (0.05 mmol), DMF (2 mL), and isolated product yields are indicated. ^a^Sulfur powder was used instead of selenium; ^b^tellurium powder was used instead of selenium.

To demonstrate the efficiency and generality of this three-component system, the reactions of Se powder (0.6 mmol) with various imidazopyridines **1** (0.5 mmol) and boronic acids **2** (0.6 mmol) were investigated under the optimized conditions (Scheme 2). Specifically, the reaction of 2-phenylimidazopyridine **1a** with Se powder and arylboron reagents **2b**–**e** bearing electron-donating and electron-withdrawing groups at the 4-position of the benzene ring afforded the desired products **9ab**–**ae** in 58–71% yields. The reaction with benzo[*b*]thiophene-2-boronic acid (**2f**) also gave the desired product **9af**. However, when (*E*)-styrylboronic acid (**2g**) or cyclohexylboronic acid (**2h**) were used, the reaction did not proceed, and the corresponding vinyl- and alkylselanyl derivatives **9ag** and **9ah** were not obtained. These results suggest that the reaction is specific to arylboronic acids. Subsequently, the reactions of substrates **1b**–**e**, which bear substituents on the phenyl group at the 2-position of the imidazopyridine moiety with Se powder and phenylboronic acid (**2a**) produced the corresponding products **9ba**–**ea** in good to moderate yields. Additionally, 2-phenylimidazopyridine derivatives **1f**–**i** bearing various electron-donating and electron-withdrawing substituents at the 6-position successfully provided the desired products **9fa**–**ha** in fair to good yields (59–65%). However, in the presence of the strongly electron-withdrawing trifluoromethyl group, the corresponding product **9ia** was formed in a low yield (31%). Notably, the unsubstituted imidazopyridine **1j** underwent a regioselective transformation to give 3-selanylimidazopyridine **9ja** in a good yield. The reactions of **1a** with sulfur or tellurium were attempted, but unfortunately, they did not proceed.

**Scheme 3:**
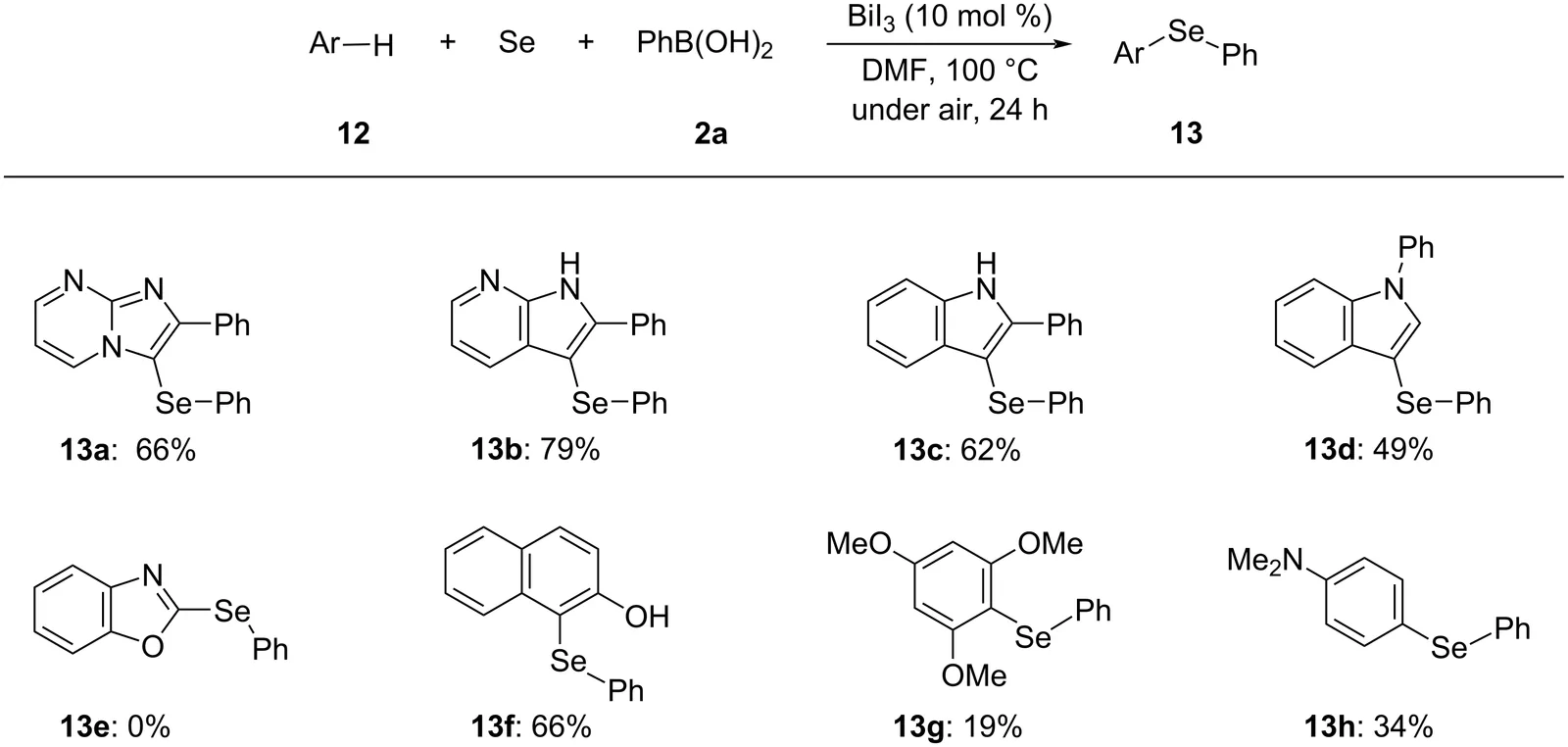
Selenation of other electron-rich arenes. Conditions: arenes **12** (0.5 mmol), Se (0.6 mmol), phenylboronic acid (**2a**, 0.6 mmol), BiI_3_ (0.05 mmol), DMF (2 mL), and isolated product yields are indicated.

To further explore the scope of this reaction, we examined its applicability not only to imidazopyridines but also to other arenes, as summarized in Scheme 3. The reaction of Se powder and boronic acid **2a** with 2-phenylimidazo[1,2-*a*]pyrimidine (**12a**) afforded the corresponding selanyl derivative **13a** in 66% yield. Furthermore, pyrrolo[2,3-*b*]pyridine (**12b**) or indoles **12c** and **12d** underwent 3-selective selenation to afford the desired products **13b**–**d** in good yield. In contrast, when benzoxazole (**12e**) was employed the target product **13e** was not generated, and only the starting material was recovered. These results suggest that both the nature and position of the heteroatoms within the aromatic ring significantly influence the reactivity. Furthermore, among the various benzene derivatives bearing activating substituents, the reaction proceeded smoothly using β-naphthol (**12f**), whereas 1,3,5-trimethoxybenzene (**12g**) and *N,N*-dimethylaniline (**12h**) gave the corresponding products in low yields, and the starting materials were recovered. These results indicate that the reaction tends to proceed more readily with electron-rich aromatic compounds or nitrogen-containing heterocycles.

**Scheme 4:**
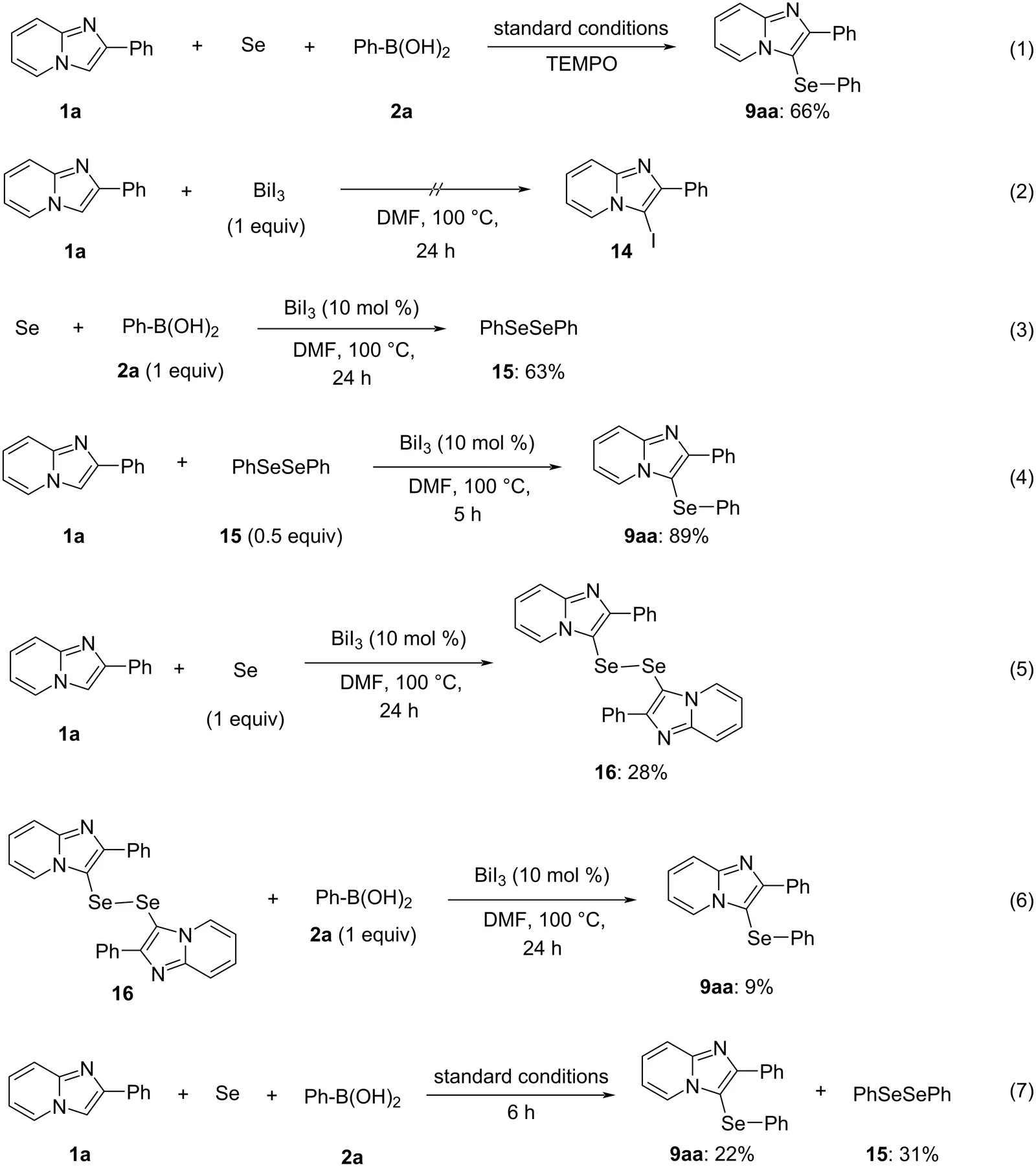
Control reactions.

Subsequently, a series of control experiments was conducted to investigate the reaction pathway and its underlying mechanism. Initially, 2-phenylimidazo[1,2-*a*]pyridine (**1a**) was reacted with Se powder and phenylboronic acid (**2a**) under standard conditions in the presence of 1 equiv of 2,2,6,6-tetramethylpiperidine 1-oxyl (TEMPO) as a radical scavenger. The reaction proceeded without a significant decrease in the yield, suggesting that a radical mechanism is unlikely (Scheme 4, reaction 1). The reaction of **1a** with BiI_3_ alone resulted in recovery of the starting material, with no formation of the iodination product **14** being observed (Scheme 4, reaction 2). In contrast, the reaction of **2a** with Se powder afforded diphenyl diselenide (**15**) in 63% yield (Scheme 4, reaction 3). Upon the subsequent reaction of **15** with **1a** in the presence of BiI_3_, the corresponding 3-(phenylselanyl)imidazopyridine **9aa** was obtained in a high yield of 89% (Scheme 4, reaction 4). Additionally, the reaction of **1a** with Se powder gave imidazopyridine-substituted diselenide **16** in a 28% yield (Scheme 4, reaction 5); however, the subsequent reaction of **16** with phenylboronic acid (**2a**) afforded the desired product **9aa** in a low yield (9%) (Scheme 4, reaction 6). Furthermore, upon quenching the three-component reaction of **1a** with Se powder and **2a** after 6 h, product **9aa** and diselenide **15** were isolated in 22% and 31% yields, respectively (Scheme 4, reaction 7). These findings suggest that the three-component reaction proceeds primarily with diphenyl diselenide **15** as the key intermediate.

**Figure 1:**
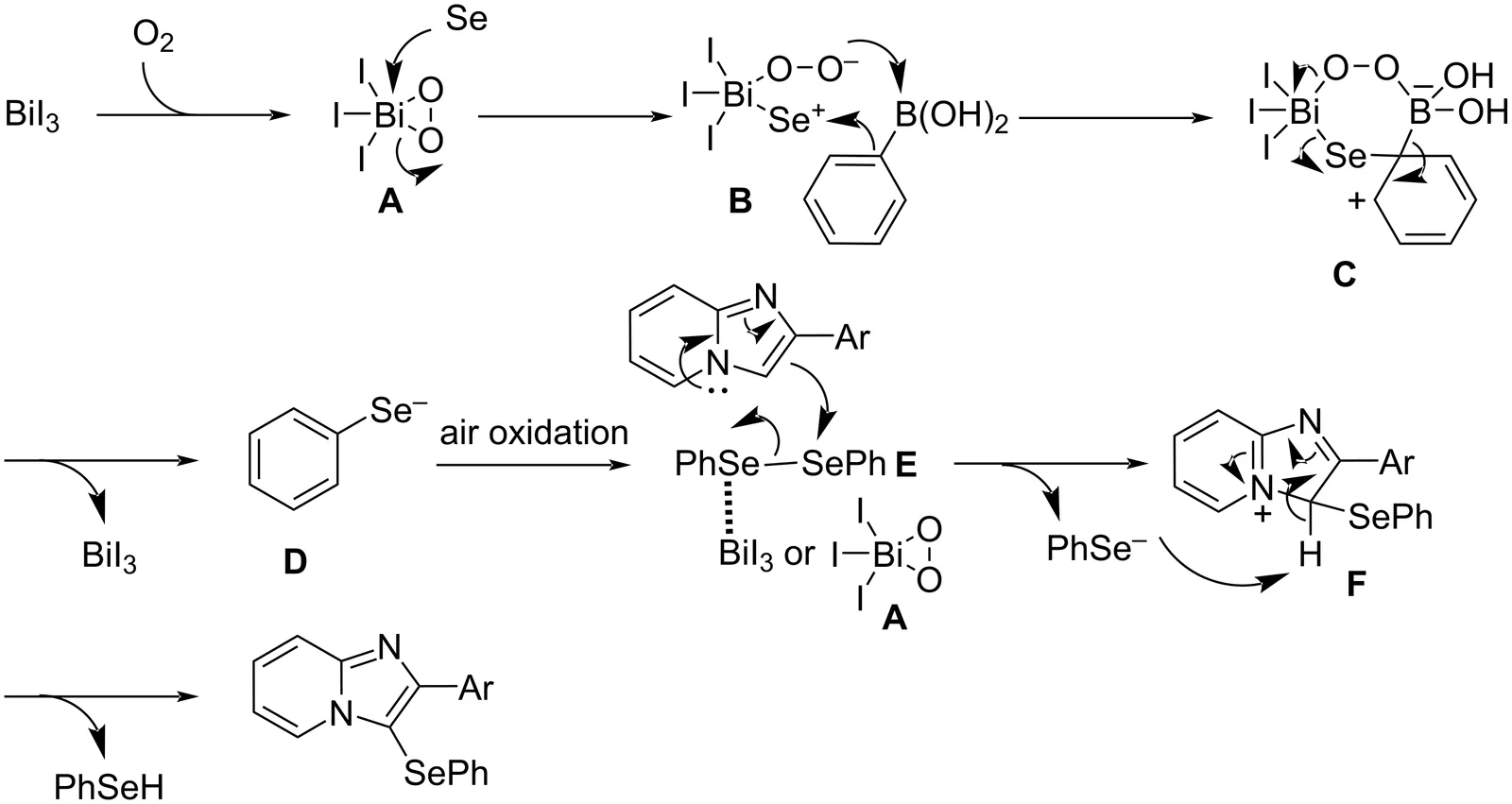
Proposed mechanism.

Although some mechanistic details remain ambiguous, a plausible reaction pathway for the three-component process is proposed in Figure 1, based on the above control experiments and the supporting evidence described below. The reaction was influenced by the surrounding atmosphere and proceeded efficiently in the presence of molecular oxygen, but was significantly inhibited under inert gas conditions (Table 1, entries 23, 27, and 28). This is likely due to the ability of BiI_3_ to form pentacoordinate complexes with oxygen-containing reagents and solvents such as Mo_8_O_26_ and THF, wherein bismuth acts as the central atom [46–47]. This process is initiated by the formation of the pentacoordinate Bi–peroxo complex **A** through the interaction of BiI_3_ with molecular oxygen. Complex **A** undergoes nucleophilic attack from Se to form intermediate **B**, which then reacts with arylboronic acid via *ipso*-substitution [48] to generate the six-membered intermediate **C**. Subsequently, the reductive elimination of BiI_3_ forms selenide anion **D**, which is then oxidized by air to give diselenide **E**. BiI_3_ or complex **A** then acts as a Lewis acid to activate diselenide **E** [49], which undergoes electrophilic substitution with the imidazopyridine to give the corresponding 3-selanylimidazopyridine via **F**. At this time, the proposed bismuth complexes **A**–**C**, which are presumed to form during the reaction, have not yet been identified or isolated, leaving this as a subject for further investigation.

## Conclusion

In conclusion, a novel bismuth-catalyzed three-component reaction was developed for the synthesis of 3-(arylselanyl)imidazopyridines from elemental selenium, arylboronic acids, and imidazopyridines. The reaction was tested using several other electron-rich arenes, suggesting a limited but possible extension of this method. This transformation proceeded under aerobic conditions without the requirement for additives. Additionally, this reaction enabled simultaneous C–H selanylation and arylselanylation to be performed under relatively mild conditions using BiI_3_ as the catalyst, which is less toxic than conventional transition-metal catalysts. Although the reaction yields require further optimization, the method provides access to potentially valuable organoselenium compounds. Studies on the mechanism of this reaction, as well as on the application of BiI_3_ as an alternative to transition-metal catalysts in other chalcogen–carbon bond-formation reactions, are currently underway.

## Experimental

### Typical procedure

A solution of imidazo[1,2-*a*]pyridine **1** or arene **12** (0.5 mmol), selenium powder (47 mg, 0.6 mmol, 1.2 equiv), arylboronic acid (0.6 mmol, 1.2 equiv), BiI_3_ (29 mg, 0.05 mmol, 10 mol %) in DMF (2 mL) was heated at 100 °C under air atmosphere for the indicated time (Scheme 2 or Scheme 3) until complete consumption of starting material was observed by TLC. After completion of the reaction, the mixture was allowed to cool to room temperature and CH_2_Cl_2_ (10 mL) and water (10 mL) were added. The aqueous layer was extracted with CH_2_Cl_2_ (20 mL × 2). The combined organic layer was washed with water (30 mL) and brine (30 mL), dried (MgSO_4_) and concentrated under reduced pressure. The crude residue was purified by column chromatography on silica gel eluting with hexane/EtOAc (**9**, **13a**, **13b**, **13e**–**g**) or hexane/CH_2_Cl_2_ (**13c**, **13d**, **13h**), to afford products **9** or **13**. The spectroscopic data of known products are in accordance with the literature.

## Supporting Information

File 1 Characterization data of all products **9** and **13**, X-ray crystallography details, and copies of spectra.

File 2 Crystallographic information file for **9ja**.

## Data Availability

All data that supports the findings of this study is available in the published article and/or the supporting information of this article.
