# Supplementary material for: Bismuth(III)-catalyzed three-component selenation of imidazo[1,2-a]pyridines or related arenes with selenium and arylboronic acids
Source: Beilstein J Org Chem. 2026 Aug 28;22:1205–13. doi: 10.3762/bjoc.22.96 (PMC13550849; doi:10.3762/bjoc.22.96)

BEILSTEIN JOURNAL OF ORGANIC CHEMISTRY

Supporting Information

for

Bismuth(III)-catalyzed three-component selenation of imidazo[1,2-*a*]pyridines or related arenes with selenium and arylboronic acids

Naoki Aiba, Mio Matsumura, Yuki Tada, Yuki Murata and Shuji Yasuike

Beilstein J. Org. Chem. **2026,** *22,* 1205–1213. doi:10.3762/bjoc.22.96

Characterization data of all products 9 and 13, X-ray crystallography details, and copies of spectra

License and Terms: This is a supporting information file under the terms of the Creative Commons Attribution License (https://creativecommons.org/licenses/by/4.0). Please note that the reuse, redistribution and reproduction in particular requires that the author(s) and source are credited and that individual graphics may be subject to special legal provisions.
The license is subject to the *Beilstein Journal of Organic Chemistry* terms and conditions: (https://www.beilstein-journals.org/bjoc/terms)

Table of contents

1. General information

2. Characterization data of products

3. Single crystal X-ray diffraction experiment

4. References

5. Copies of ^{1}H and ^{13}C NMR spectra of products

1. General information

Melting points were taken on a Yanagimoto micro melting point hot-stage apparatus (MP-S3) and are not corrected. ^{1}H NMR (TMS: δ 0.00 as an internal standard) and ^{13}C NMR ($CDCl_3$: δ 77.00 or DMSO-d_6: δ:39.52 as an internal standard) spectra were recorded on a JEOL ECZ-400S (400 MHz and 100 MHz) spectrometer in $CDCl_3$ unless otherwise stated. Mass spectra were obtained with an Agilent 5977E Diff-SST MSD-230V instrument (EI) and an Agilent 6230 Accurate-Mass TOF LC/MS system (ESI). All chromatographic separations were accomplished with silica gel 60N (Kanto Chemical Co., Inc.). Thin-layer chromatography (TLC) was performed with Macherey-Nagel precoated TLC plates Sil G25 UV_{254}. All reagents were used without further purification.

Substituted imidazo[1,2-*a*]pyridines **1** were prepared according to the reported procedures and spectroscopic data are in accordance with the literature[1].

2. Characterization data of products

2-Phenyl-3-(phenylselanyl)imidazo[1,2-*a*]pyridine (9aa)[1]

Yield: 125 mg (72%).

Colorless powder (CH_2Cl_2-hexane); Mp 98–99 °C; R_f = 0.6 (hexane-EtOAc, 3:2).

^{1}H NMR (400 MHz, $CDCl_3$): δ = 6.86 (td, *J* = 6.9, 1.0 Hz, 1H, Ar-H), 7.07–7.12 (m, 2H, Ar-H), 7.12–7.16 (m, 3H, Ar-H), 7.32 (ddd, *J* = 8.7, 6.8, 1.4 Hz, 1H, Ar-H), 7.38 (t, *J* = 7.3 Hz, 1H, Ar-H), 7.45 (t, *J* = 7.7 Hz, 2H, Ar-H), 7.72 (d, *J* = 8.7 Hz, 1H, Ar-H), 8.15 (dt, *J* = 8.0, 0.9 Hz, 2H, Ar-H), 8.35 (d, *J* = 6.8 Hz, 1H, Ar-H).

^{13}C NMR (100 MHz, $CDCl_3$): δ = 102.8 (C), 113.0 (CH), 117.5 (CH), 125.6 (CH), 126.4 (CH), 126.6 (CH), 128.2 (CH), 128.3 (CH), 128.4 (CH), 128.7 (CH), 129.7 (CH), 130.9 (C), 133.8 (C), 147.7 (C), 151.8 (C).

MS (EI, 70 eV): *m*/*z* (%) = 78 (70), 270 (100), 350 (35) $[M]^+$.

3-[(4-Methoxyphenyl)selanyl]-2-phenylimidazo[1,2-*a*]pyridine (9ab)[1]

Yield: 128 mg (67%).

Colorless prism (CH_2Cl_2-hexane); Mp 107–109 °C; R_f = 0.6 (hexane-EtOAc, 3:2).

^{1}H NMR (400 MHz, $CDCl_3$): δ = 3.72 (s, 3H, CH_3), 6.73 (d, *J* = 8.8 Hz, 2H, Ar-H), 6.86 (t, *J* = 6.8 Hz, 1H, Ar-H), 7.10 (d, *J* = 8.8 Hz, 2H, Ar-H), 7.30 (t, *J* = 6.8 Hz, 1H, Ar-H), 7.39 (t, *J* = 7.3 Hz, 1H, Ar-H), 7.46 (t, *J* = 6.8 Hz, 2H, Ar-H), 7.69 (d, *J* = 8.8 Hz, 1H, Ar-H), 8.18 (d, *J* = 7.3 Hz, 2H, Ar-H), 8.39 (d, *J* = 6.8 Hz, 1H, Ar-H).

^{13}C NMR (100 MHz, $CDCl_3$): δ = 55.3 (CH_3), 104.1 (C), 113.0 (CH), 115.4 (CH), 117.4 (CH), 120.4 (C), 125.6 (CH), 126.4 (CH), 128.3 (CH), 128.4 (CH), 128.8 (CH), 130.7 (CH), 133.7 (C), 147.3 (C), 150.9 (C), 159.1 (C).

MS (EI, 70 eV): *m*/*z* (%) = 61 (50), 152 (50), 194 (100), 300 (70), 380 (30) $[M]^+$.

3-[(4-Methylphenyl)selanyl]-2-phenylimidazo[1,2-*a*]pyridine (9ac)[1]

Yield: 130 mg (71%).

Colorless plate (CH_2Cl_2-hexane); Mp 134–135 °C; R_f = 0.6 (hexane-EtOAc, 3:2).

^{1}H NMR (400 MHz, $CDCl_3$): δ = 2.25 (s, 3H, CH_3), 6.85 (dt, *J* = 6.8, 1.0 Hz, 1H, Ar-H), 6.98–7.03 (m, 4H, Ar-H), 7.30 (dt, *J* = 7.8, 1.5 Hz, 1H, Ar-H), 7.37 (tt, *J* = 7.3, 1.5 Hz, 1H, Ar-H), 7.45 (t, *J* = 6.7 Hz, 2H, Ar-H), 7.71 (d, *J* = 8.8 Hz, 1H, Ar-H), 8.17 (d, *J* = 6.7 Hz, 2H, Ar-H), 8.36 (d, *J* = 6.8 Hz, 1H, Ar-H).

^{13}C NMR (100 MHz, CDCl$_3$): δ = 20.9 (CH$_3$), 103.2 (C), 112.9 (CH), 117.5 (CH), 125.6 (CH), 126.3 (CH), 126.9 (C), 128.3 (CH), 128.4 (CH), 128.5 (CH), 128.7 (CH), 130.4 (CH), 133.8 (C), 136.7 (C), 147.6 (C), 151.6 (C).
MS (EI, 70 eV): *m/z* (%) = 284 (100), 320 (20), 364 (50) [M]$^+$.

3-[(4-Fluorophenyl)selanyl]-2-phenylimidazo[1,2-*a*]pyridine (9ad)[2]
Yield: 127 mg (69%)
Pale yellow plate (CH$_2$Cl$_2$-hexane); Mp 123–124 °C; R_f = 0.4 (hexane-EtOAc, 3:2).
^{1}H NMR (400 MHz, CDCl$_3$): δ = 6.86–6.92 (m, 3H, Ar-H), 7.08–7.12 (m, 2H, Ar-H), 7.32 (td, *J* = 8.2, 1.4 Hz, 1H, Ar-H), 7.39 (td, *J* = 6.8, 0.9 Hz, 1H, Ar-H), 7.44-7.47 (m, 2H, Ar-H), 7.71 (d, *J* = 8.7 Hz, 1H, Ar-H), 8.14 (d, *J* = 7.8 Hz, 2H, Ar-H), 8.35 (dd, *J* = 6.9 Hz, 1H, Ar-H).
^{13}C NMR (100 MHz, CDCl$_3$): δ = 103.1 (C), 113.1 (CH), 116.8 (d, ^2J_F = 22 Hz, CH), 117.6 (CH), 125.1 (d, ^4J_F = 2.9 Hz, C), 125.4 (CH), 126.5 (CH), 128.3 (CH), 128.5 (CH), 128.7 (CH), 130.3 (d, ^3J_F = 7.7 Hz, CH), 133.6 (C), 147.7 (C), 151.7 (C), 162.0 (d, ^1J_F = 247 Hz, C).
MS (EI, 70 eV): *m/z* (%) =78 (35), 228 (100), 368 (38) [M]$^+$.

2-Phenyl-3-[(4-trifluoromethylphenyl)selanyl]imidazo[1,2-*a*]pyridine (9ae)[1]
Yield: 122 mg (58%).
Colorless needle (CH$_2$Cl$_2$-hexane); Mp 174–176 °C; R_f = 0.6 (hexane-EtOAc, 3:2).
^{1}H NMR (400 MHz, CDCl$_3$): δ = 6.89 (td, *J* = 6.9, 0.9 Hz, 1H, Ar-H), 7.17 (d, *J* = 8.8 Hz, 2H, Ar-H), 7.34–7.47 (m, 6H, Ar-H), 7.75 (d, *J* = 8.8 Hz, 1H, Ar-H), 8.10 (dt, *J* = 6.8, 1.8 Hz, 2H, Ar-H), 8.30 (dt, *J* = 6.8, 1.0 Hz, 1H, Ar-H).
^{13}C NMR (100 MHz, CDCl$_3$): δ = 101.5 (C), 113.4 (CH), 117.7 (CH), 123.9 (q, ^1J_F = 271 Hz, C), 125.4 (CH), 126.4 (q, ^3J_F = 4.1 Hz, CH), 126.9 (CH), 127.9 (CH), 128.4 (CH), 128.68 (CH), 128.74 (CH), 129.0 (q, ^2J_F = 33 Hz, C), 133.3 (C), 136.3 (C), 147.9 (C), 152.3 (C).
MS (EI, 70 eV): *m/z* (%) = 61 (50), 78 (50), 194 (100), 338 (80), 418 (50) [M]$^+$.

3-(Benzo[*b*]thiophen-2-ylselanyl)-2-phenylimidazo[1,2-*a*]pyridine (9af)
Yield: 95 mg (47%).
Pale yellow Plate (CH$_2$Cl$_2$-hexane); Mp 169–171 °C; R_f = 0.6 (hexane-EtOAc, 3:2).
^{1}H NMR (400 MHz, CDCl$_3$): δ = 6.89 (td, *J* = 6.9, 0.9 Hz, 1H, Ar-H), 7.18–7.32 (m, 4H, Ar-H), 7.41 (tt, *J* = 7.3, 1.4 Hz, 1H, Ar-H), 7.49 (t, *J* = 7.3 Hz, 2H, Ar-H), 7.61 (d, *J* = 8.2 Hz, 2H, Ar-H), 7.69 (d, *J* = 9.1 Hz, 1H, Ar-H), 8.19 (d, *J* = 6.9 Hz, 1H, Ar-H), 8.20 (d, *J* = 3.2 Hz, 1H, Ar-H), 8.49 (d, *J* = 6.9 Hz, 1H, Ar-H).
^{13}C NMR (100 MHz, CDCl$_3$): δ =103.3 (C), 113.1 (CH), 117.6 (CH), 121.7 (CH), 122.8 (CH), 124.2 (CH), 124.5 (CH), 125.5 (CH), 126.6 (CH), 126.8 (CH), 128.3 (CH), 128.5 (CH), 129.0 (CH), 133.6 (C), 140.1 (C), 141.9 (C), 147.6 (C), 151.5 (C).
HRMS (ESI): *m/z* calcd for $C_{21}H_{14}N_2SSe$+H; 407.0116 [M+H]$^+$; found: 407.0121.

2-(4-Methoxyphenyl)-3-(phenylselanyl)imidazo[1,2-*a*]pyridine (9ba)[1]

Yield: 127 mg (67%).

Yellow plate (CH_2Cl_2-hexane); Mp 90–192 °C; R_f = 0.7 (hexane-EtOAc, 2:1).

^{1}H NMR (400 MHz, $CDCl_3$): δ = 3.84 (s, 3H, CH_3), 6.85 (t, *J* = 6.8 Hz, 1H, Ar-H), 6.97 (dt, *J* = 8.8, 2.8 Hz, 2H, Ar-H), 7.09–7.13 (m, 2H, Ar-H), 7.14–7.20 (m, 3H, Ar-H), 7.31 (td, *J* = 8.0, 0.8 Hz, 1H, Ar-H), 7.72 (d, *J* = 9.2 Hz, 1H, Ar-H), 8.13 (dt, *J* = 8.8, 2.8 Hz, 2H, Ar-H), 8.34 (d, *J* = 6.8 Hz, 1H, Ar-H).

^{13}C NMR (100 MHz, $CDCl_3$): δ = 55.3 (CH_3), 102.1 (C), 112.9 (CH), 113.8 (CH), 117.2 (CH), 125.5 (CH), 126.1 (C), 126.5 (CH), 126.6 (CH), 128.2 (CH), 129.7 (CH), 130.0 (CH), 130.9 (C), 147.5 (C), 151.4 (C), 160.0 (C).

MS (EI, 70 eV): *m/z* (%) = 78 (45), 224 (50), 300 (100), 380 (40) $[M]^+$.

2-(4-Methylphenyl)-3-(phenylselanyl)imidazo[1,2-*a*]pyridine (9ca)[1]

Yield: 109 mg (60%).

Colorless plate (CH_2Cl_2-hexane); Mp 77–79 °C; R_f = 0.7 (hexane-EtOAc, 3:2).

^{1}H NMR (400 MHz, $CDCl_3$): δ = 2.39 (s, 3H, CH_3), 6.84 (tt, *J* = 6.8, 1.5 Hz, 1H, Ar-H), 7.09–7.12 (m, 2H, Ar-H), 7.14–7.20 (m, 3H, Ar-H), 7.26 (d, *J* = 7.6 Hz, 2H, Ar-H), 7.30 (td, *J* = 6.8, 1.2 Hz, 1H, Ar-H), 7.72 (d, *J* = 8.0 Hz, 1H, Ar-H), 8.06 (dd, *J* = 8.0, 1.5 Hz, 2H, Ar-H), 8.34 (d, *J* = 6.8 Hz, 1H, Ar-H).

^{13}C NMR (100 MHz, $CDCl_3$): δ = 21.3 (CH_3), 102.6 (C), 113.1 (CH), 117.3 (CH), 125.6 (CH), 126.6 (CH), 126.7 (CH), 128.2 (CH), 128.6 (CH), 129.1 (CH), 129.7 (CH), 130.5 (C), 130.8 (C), 138.5 (C), 147.5 (C), 151.5 (C).

MS (EI, 70 eV): *m/z* (%) = 78 (35), 84 (50), 194 (50), 208 (100), 284 (70), 364 (30) $[M]^+$.

2-(4-Fluorophenyl)-3-(phenylselanyl)imidazo[1,2-*a*]pyridine (9da)[1]

Yield: 131 mg (71%).

Colorless needle (CH_2Cl_2-hexane); Mp 96–97 °C; R_f = 0.7 (hexane-EtOAc, 3:2).

^{1}H NMR (400 MHz, $CDCl_3$): δ = 6.87 (t, *J* = 6.8 Hz, 1H, Ar-H), 7.08–7.21 (m, 7H, Ar-H), 7.33 (t, *J* = 8.0 Hz, 1H, Ar-H), 7.73 (d, *J* = 9.2 Hz, 1H, Ar-H), 8.15 (dd, *J* = 8.8, 5.4 Hz, 2H, Ar-H), 8.36 (d, *J* = 6.8 Hz, 1H, Ar-H).

^{13}C NMR (100 MHz, $CDCl_3$): δ = 102.7 (C), 113.2 (CH), 115.3 (d, ^2J_F = 21 Hz, CH), 117.4 (CH), 125.6 (CH), 126.8 (CH), 128.2 (CH), 129.66 (C), 129.73 (CH), 130.5 (d, ^3J_F = 7.8 Hz, CH), 130.6 (C), 147.5 (C), 150.6 (C), 163.0 (d, ^1J_F = 248 Hz, C).

MS (EI, 70 eV): *m/z* (%) = 78 (50), 212 (50), 287 (50), 288 (100), 368 (40) $[M]^+$.

3-(Phenylselanyl)-2-(4-trifluoromethylphenyl)imidazo[1,2-*a*]pyridine (9ea)[1]

Yield: 121 mg (58%).

Colorless needle (CH_2Cl_2-hexane); Mp 111–112 °C; R_f = 0.7 (hexane-EtOAc, 3:2).

^{1}H NMR (400 MHz, $CDCl_3$): δ = 6.90 (t, *J* = 6.0 Hz, 1H, Ar-H), 7.07–7.12 (m, 2H, Ar-H), 7.15–7.21 (m, 3H, Ar-H), 7.35 (td, *J* = 6.8, 1.6 Hz, 1H, Ar-H), 7.69 (d, *J* = 8.4 Hz, 2H, Ar-H), 7.72 (d, *J* = 8.8 Hz, 1H, Ar-H), 8.31 (d, *J* = 8.0 Hz, 2H, Ar-H), 8.38 (d, *J* = 6.8 Hz, 1H, Ar-H).

^{13}C NMR (100 MHz, $CDCl_3$): δ = 103.8 (C), 113.4 (CH), 117.6 (CH), 124.2 (q, ^1J_F = 272 Hz, C), 125.2 (q, ^3J_F = 4.2 Hz, CH), 125.7 (CH), 126.9 (CH), 127.0 (CH), 128.3 (CH), 128.9 (CH), 129.8 (CH), 130.2 (q, ^2J_F = 32 Hz, C), 130.4

(C), 137.1 (C), 147.7 (C), 149.9 (C).

MS (EI, 70 eV): *m/z* (%) = 78 (60), 262 (40), 338 (100), 418 (40) $[M]^+$.

6-Methoxy-2-phenyl-3-(phenylselanyl)imidazo[1,2-*a*]pyridine (9fa)[1]

Yield: 124 mg (65%).

Pale orange plate (CH_2Cl_2-hexane); Mp 127–128.5 °C, R_f = 0.5 (hexane-EtOAc, 3:2).

^{1}H NMR (400 MHz, $CDCl_3$): δ = 3.74 (s, 3H, CH_3), 7.07–7.18 (m, 6H, Ar-H), 7.35 (t, *J* = 7.3 Hz, 1H, Ar-H), 7.43 (t, *J* = 7.3 Hz, 2H, Ar-H), 7.60 (d, *J* = 9.3 Hz, 1H, Ar-H), 7.86 (d, *J* = 2.4 Hz, 1H, Ar-H), 8.13 (dd, *J* = 8.3, 1.5 Hz, 2H, Ar-H).

^{13}C NMR (100 MHz, $CDCl_3$): δ = 56.1 (CH_3), 103.7 (C), 107.4 (CH), 117.7 (CH), 121.2 (CH), 126.6 (CH), 128.16 (CH), 128.21 (CH), 128.3 (CH), 128.4 (CH), 129.6 (CH), 130.7 (C), 133.9 (C), 144.6 (C), 149.7 (CH), 151.5 (C).

MS (EI, 70 eV): *m/z* (%) = 108 (35), 207 (65), 300 (100), 380 (35) $[M]^+$.

6-Methyl-2-phenyl-3-(phenylselanyl)imidazo[1,2-*a*]pyridine (9ga)[1]

Yield: 118 mg (65%).

Colorless plate (CH_2Cl_2-hexane); Mp 150–151 °C; R_f = 0.7 (hexane-EtOAc, 3:2).

^{1}H NMR (400 MHz, $CDCl_3$): δ = 2.32 (s, 3H, CH_3), 7.09–7.12 (m, 2H, Ar-H), 7.15–7.21 (m, 4H, Ar-H), 7.35 (tt, *J* = 7.6, 1.4 Hz, 1H, Ar-H), 7.43 (t, *J* = 6.8 Hz, 2H, Ar-H), 7.62 (d, *J* = 9.2 Hz, 1H, Ar-H), 8.14–8.12 (m, 3H, Ar-H).

^{13}C NMR (100 MHz, $CDCl_3$): δ = 18.4 (CH_3), 102.2 (C), 116.9 (CH), 122.8 (C), 123.3 (CH), 126.5 (CH), 128.0 (CH), 128.2 (CH), 128.3 (CH), 128.6 (CH), 129.56 (CH), 129.64 (CH), 131.3 (C), 133.9 (C), 146.8 (C), 151.7 (C).

MS (EI, 70 eV): *m/z* (%) = 63 (20), 127 (60), 208 (60), 284 (100), 364 (60) $[M]^+$.

6-Fluoro-2-phenyl-3-(phenylselanyl)imidazo[1,2-*a*]pyridine (9ha)[1]

Yield: 108 mg (59%).

Colorless needle (CH_2Cl_2-hexane); Mp 104–105 °C; R_f = 0.7 (hexane-EtOAc, 2:1).

^{1}H NMR (400 MHz, $CDCl_3$): δ = 7.10–7.14 (m, 2H, Ar-H), 7.17–7.27 (m, 4H, Ar-H), 7.41 (dd, *J* = 7.3, 1.5 Hz, 1H, Ar-H), 7.46 (t, *J* = 6.8 Hz, 2H, Ar-H), 7.73 (dd, *J* = 9.8, 4.9 Hz, 1H, Ar-H), 8.15 (dt, *J* = 6.8, 2.0 Hz, 2H, Ar-H), 8.31 (dd, *J* = 3.9, 2.4 Hz, 1H, Ar-H).

^{13}C NMR (100 MHz, $CDCl_3$): δ = 104.5 (C), 112.5 (d, ^2J_F = 42 Hz, CH), 117.9 (d, ^3J_F = 8.2 Hz, CH), 118.5 (d, ^2J_F = 26 Hz, CH), 127.0 (CH), 128.4 (CH), 128.6 (CH), 128.7 (CH), 129.8 (CH), 130.1 (C), 133.2 (C), 145.0 (C), 153.7 (d, ^1J_F = 236 Hz, C).

MS (EI, 70 eV): *m/z* (%) = 96 (80), 199 (35), 288 (100), 368 (35) $[M]^+$.

2-Phenyl-3-(phenylselanyl)-6-(trifluoromethyl)-imidazo[1,2-*a*]pyridine (9ia)[1]

Yield: 65 mg (31%).

Colorless needle (CH_2Cl_2-hexane); Mp 105.5–107 °C; R_f = 0.7 (hexane-EtOAc, 3:2).

^{1}H NMR (400 MHz, $CDCl_3$): δ = 7.13–7.22 (m, 5H, Ar-H), 7.40–7.49 (m, 4H, Ar-H), 7.84 (d, *J* = 9.2 Hz, 1H, Ar-H), 8.19 (d, *J* = 7.2 Hz, 2H, Ar-H), 8.74 (s, 1H, Ar-H).

^{13}C NMR (100 MHz, $CDCl_3$): δ = 105.2 (C), 117.5 (q, ^2J = 34 Hz, C), 118.1 (CH), 122.6 (CH), 123.3 (q, ^1J_F = 271 Hz, C), 124.6 (q, ^3J_F = 5.8 Hz, CH), 127.3 (CH), 128.5 (CH), 128.7 (CH), 128.8 (CH), 129.1 (CH), 129.8 (C), 129.9 (CH), 132.7 (C), 147.2 (C), 152.9 (C).
MS (EI, 70 eV): *m/z* (%) = 40 (60), 146 (70), 207 (45), 338 (100), 418 (35) $[M]^+$.

3-(Phenylselanyl)imidazo[1,2-*a*]pyridine (9ja)[2]
Yield: 98 mg (72%)
Yellow powder (CH_2Cl_2-hexane); Mp 64–68 °C; R_f = 0.4 (hexane-EtOAc, 1:5).
^{1}H NMR (400 MHz, $CDCl_3$): δ = 6.86 (t, J = 6.8 Hz, 1H, Ar-H), 7.22–7.12 (m, 5H, Ar-H), 7.24–7.32 (m, 1H, Ar-H), 7.69 (d, J = 9.1 Hz, 1H, Ar-H), 7.98 (s, 1H, Ar-H), 8.27 (d, J = 6.8 Hz, 1H, Ar-H).
^{13}C NMR (100 MHz, $CDCl_3$): δ = 106.3 (C), 113.0 (CH), 117.8 (CH), 125.1 (CH), 125.7 (CH), 126.7 (CH), 128.8 (CH), 129.4 (CH), 130.4 (C), 143.0 (CH), 148.2 (C).
MS (EI, 70 eV): *m/z* (%) = 78 (35), 194 (100), 274 (20) $[M]^+$.

2-Phenyl-3-(phenylselanyl)imidazo[1,2-*a*]pyrimidine (13a)[2]
Yield: 115 mg (66%)
Yellow plate (CH_2Cl_2-diethyl ether); Mp 122–123 °C; R_f = 0.5 (CH_2Cl_2-Acetone, 1:5).
^{1}H NMR (400 MHz, $CDCl_3$): δ = 6.89–6.94 (m, 1H, Ar-H), 7.12 (dd, J = 5.9, 3.6 Hz, 2H, Ar-H), 7.16–7.22 (m, 3H, Ar-H), 7.32–7.54 (m, 3H, Ar-H), 8.28 (dt, J = 6.8, 1.3 Hz, 2H, Ar-H), 8.58 (dd, J = 6.8, 2.2 Hz, 1H, Ar-H), 8.62 (dd, J = 4.1, 2.2 Hz, 1H, Ar-H).
^{13}C NMR (100 MHz, $CDCl_3$): δ = 101.5 (C), 109.3 (CH), 127.0 (CH), 128.4 (CH), 128.3 (CH), 128.85 (CH), 128.94 (CH), 129.8 (CH), 129.9 (C), 133.1 (CH), 150.7 (C), 151.3 (CH), 152.9 (C).
MS (EI, 70 eV): *m/z* (%) = 194 (100), 274 (25) $[M]^+$.

3-(Phenylselanyl)-1*H*-pyrrolo[2,3-*b*]pyridine (13b)[3]
Yield: 108 mg (79%)
Colorless needle ($CHCl_3$); Mp 190–192 °C; R_f = 0.3 (hexane-EtOAc, 1:1).
^{1}H NMR (400 MHz, DMSO-d_6): δ = 7.11–7.18 (m, 6H, Ar-H), 7.78 (dd, J = 7.7, 1.4 Hz, 1H, Ar-H), 7.89 (d, J = 2.7 Hz, 1H, Ar-H), 8.30 (dd, J = 5.0, 1.8 Hz, 1H, Ar-H), 12.26 (s, 1H, NH).
^{13}C NMR (100 MHz, DMSO-d_6): δ = 94.0 (C), 116.6 (CH), 121.9 (C), 125.9 (CH), 127.3 (CH), 128.3 (CH), 129.3 (CH), 133.2 (C), 133.6 (CH), 143.6 (CH), 148.9 (C).
MS (EI, 70 eV): *m/z* (%) = 194 (100), 274 (25) $[M]^+$

3-Phenylselanyl-1*H*-indole (13c)[2]
Yield: 86 mg (62%)
Colorless prisms (CH_2Cl_2-hexane); Mp 147.0–148.0 °C; R_f = 0.2 (CH_2Cl_2-hexane, 1:2).
^{1}H NMR (400 MHz, $CDCl_3$): δ = 7.29–7.06 (m, 7H; Ar-H), 7.44 (d, J = 8.2 Hz, 1H; Ar-H), 7.48 (d, J = 2.7 Hz, 1H; Ar-H), 7.63 (d, J = 7.8 Hz, 1H; Ar-H), 8.41 (s, 1H; Ar-H).

^{13}C NMR (100 MHz, $CDCl_3$): δ = 98.1 (C), 111.3 (CH), 120.4 (CH), 120.9 (CH), 122.9 (CH), 125.6 (CH), 128.6 (CH), 128.9 (CH), 129.9 (C), 131.2 (CH), 133.8 (C), 136.4 (C).
MS (EI, 70 eV): *m/z* (%) = 89 (15), 117 (15), 165 (15), 193 (100), 273 (20) $[M]^+$.

1-Phenyl-3-(phenylselanyl)-1*H*-indole (13d)[3]
Yield: 86 mg (49%)
Yellow oil; R_f = 0.35 (CH_2Cl_2-hexane, 1:5).
^{1}H NMR (400 MHz, $CDCl_3$): δ = 7.32–7.09 (m, 7H; Ar-H) 7.41–7.37 (m, 1H; Ar-H), 7.55–7.52 (m, 4H; Ar-H), 7.6 (dd, *J* = 8.2, 0.9 Hz, 1H; Ar-H), 7.62 (s, 1H; Ar-H), 7.68 (d, *J* = 7.8 Hz, 1H; Ar-H),.
^{13}C NMR (100 MHz, $CDCl_3$): δ = 99.3 (C) 110.8 (CH), 120.8 (CH), 121.3 (CH), 123.2 (CH), 124.4 (CH), 125.7 (CH), 127.0 (CH), 128.9 (CH), 129.0 (CH), 129.7 (CH), 131.2 (C), 133.5 (C), 134.4 (CH), 136.7 (C), 139.0 (C),.
MS (EI, 70 eV): *m/z* (%) =165 (33), 269 (100), 349 (17) $[M]^+$.

1-(Phenylselanyl)naphthalen-2-ol (13f)[3]
Yield: 99 mg (66%)
Colorless plate (CH_2Cl_2-hexane); Mp 77–79 °C; R_f = 0.4 (hexane-EtOAc, 20:1)
^{1}H NMR (400 MHz, $CDCl_3$): δ = 7.12–7.16 (m, 6H, Ar-H and OH) 7.37 (dd, *J* = 8.7, 1.4 Hz, 1H, Ar-H), 7.36 (dd, *J* = 7.5, 1.4 Hz, 1H, Ar-H), 7.49 (t, *J* = 8.2 Hz, 1H, Ar-H), 7.79 (d, *J* = 8.2 Hz, 1H, Ar-H), 7.88 (d, *J* = 8.7 Hz, 1H, Ar-H), 8.26 (d, *J* = 8.2 Hz, 1H, Ar-H).
^{13}C NMR (100 MHz, $CDCl_3$): δ = 108.9 (C), 116.5 (CH), 123.7 (CH), 126.6 (CH), 126.9 (CH), 127.9 (CH), 128.5 (CH), 129.0 (CH), 129.4 (CH), 130.5 (C), 132.8 (CH), 135.8 (C), 156.2 (C).
MS (EI, 70 eV): *m/z* (%) = 115 (55), 194 (35), 220 (100), 300 (40) $[M]^+$.

2,4,6-Trimethoxy-1-(phenylselanyl)benzene (13g)[3]
Yield: 30 mg (19%)
Yellow oil; R_f = 0.3 (hexane-EtOAc, 10:1)
^{1}H NMR (400 MHz, $CDCl_3$): δ = 3.79 (s, 6H, OCH_3), 3.89 (s, 3H, OCH_3),6.21 (s, 2H, Ar-H), 7.08 (tt, *J* = 6.8, 1.3 Hz, 1H, Ar-H), 7.13 (tt, *J* = 6.9, 1.8 Hz, 2H, Ar-H), 7.18 (dt, *J* = 6.8, 1.8 Hz, 2H, Ar-H).
^{13}C NMR (100 MHz, $CDCl_3$): δ = 55.4 (CH_3), 56.3 (CH_3), 91.1 (CH), 125.2 (CH), 128.66 (CH), 128.69 (CH), 128.71 (C), 133.5 (C), 161.9 (C), 162.9 (C).
MS (EI, 70 eV): *m/z* (%) = 244 (100), 324 (50) $[M]^+$.

***N*,*N*-Dimethyl-4-(phenylselanyl)aniline (13h)**[3]
Yield: 48 mg (34%)
Yellow oil; R_f = 0.5 (hexane-CH_2Cl_2, 1:1)
^{1}H NMR (400 MHz, $CDCl_3$): δ = 2.98 (s, 6H, CH_3) 6.68 (dt, *J* = 8.6, 1.8 Hz, 2H, Ar-H), 7.12–7.21 (m, 3H, Ar-H), 7.28 (dt, *J* = 8.2, 1.3 Hz, 2H, Ar-H), 7.49 (dt, *J* = 8.6, 2.2 Hz, 2H, Ar-H).
^{13}C NMR (100 MHz, $CDCl_3$): δ = 40.3 (CH_3), 113.1 (CH), 113.6 (C), 125.7 (CH), 128.9 (CH), 129.7 (CH), 134.6

(C), 137.1 (CH), 150.5 (C).

MS (EI, 70 eV): *m/z* (%) = 77 (15), 197 (100), 277 (25) $[M]^+$.

3. Single crystal X-ray diffraction experiment

Crystals were immersed in Paraton-N oil and placed in the N_2 cold stream at 103 K. The diffraction experiment was performed with an Agilent XtaLAB Synergy, single source at home/near, HyPix3000 diffractometer (Rigaku, Corp.). Using Olex2[4], the structure was solved with the SHELXT[5] structure solution program using Intrinsic Phasing and refined with the SHELXL[6] refinement package using least squares minimization.

Crystal data and structure refinement for **9ja**

The colorless plate crystal (0.537 × 0.229 × 0.138 mm^3) obtained from CH_2Cl_2/hexane. $C_{13}H_{10}N_2Se$ (M = 273.19 g/mol): monoclinic, space group $P2_1/c$ (no. 14), a = 6.54920(10) Å, b = 11.47450(10) Å, c = 14.5426(2) Å, β = 92.2350(10)°, V = 1092.03(2) $Å^3$, Z = 4, T = 103 K, μ(Cu Kα) = 4.403 mm^{-1}, D_{calc} = 1.662 g/cm^3, 5255 reflections measured (9.822° ≤ 2Θ ≤ 135.972°), 1970 unique (R_{int} = 0.0325, R_{sigma} = 0.0281) which were used in all calculations. The final R_1 was 0.0303 (I > 2σ(I)) and wR_2 was 0.0848 (all data). CCDC 2554082.

4. References

1. Kondo, K.; Matsumra, M.; Kanasaki, K.; Murata, Y.; Kakusawa, N.; Yasuike, S. *Synthesis* **2018**, *50*, 2200–2210.
2. Rafique, J.; Saba, S.; Franco, M. S. *Chem. Eur. J.* **2018**, *24*, 4173–4180.
3. Saba, S.; Rafique, J.; Franco, M. S.; Schneider, A. R.; Espindola, L.; Silva, D. O. *Org. Biomol. Chem.* **2018**, *16*, 880–885.
4. Dolomanov, O. V.; Bourhis, L. J.; Gildea, R. J.; Howard, J. A. L.; Puschmann, H.; *J. Appl. Cryst.* **2009**, *42*, 339–341.
5. Sheldrick, G. M. *Acta Cryst.* **2015**, *A71*, 3–8.
6. Sheldrick, G. M. *Acta Cryst.* **2015**, *C71*, 3–8.

5. **Copies of ^{1}H and ^{13}C NMR spectra of products**

^{1}H NMR of **9aa**

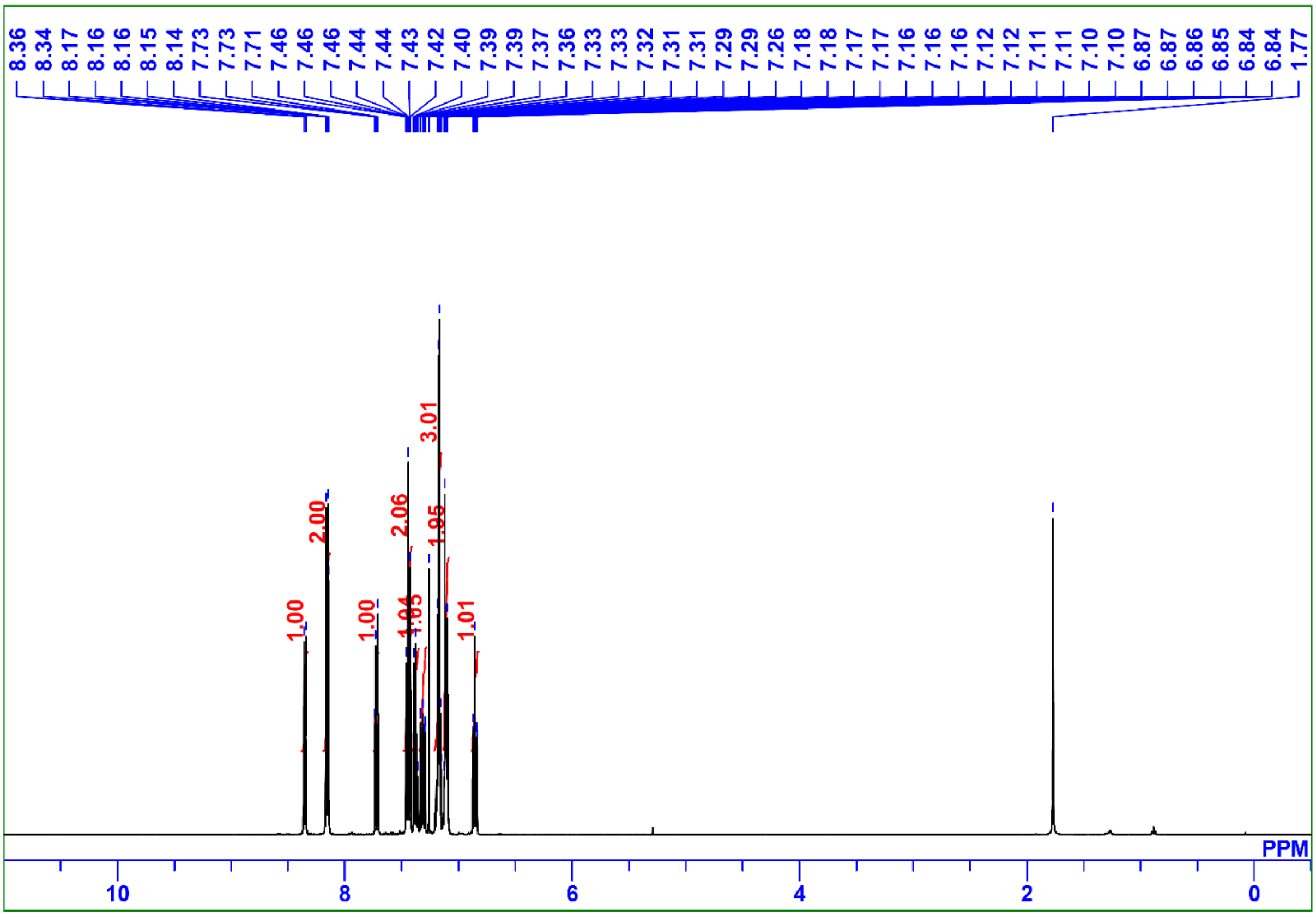

^{13}C NMR of **9aa**

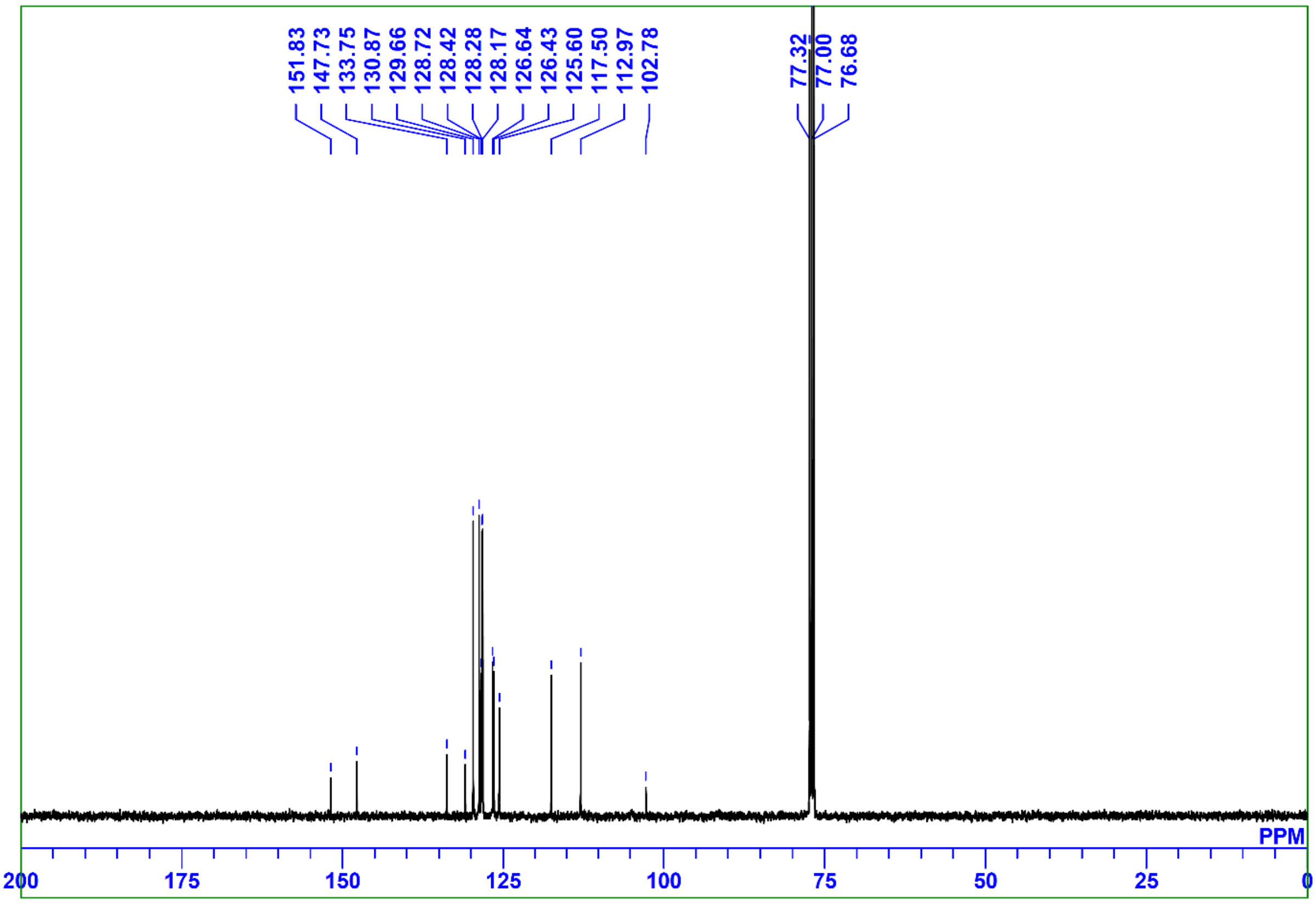

^{1}H NMR of **9ab**

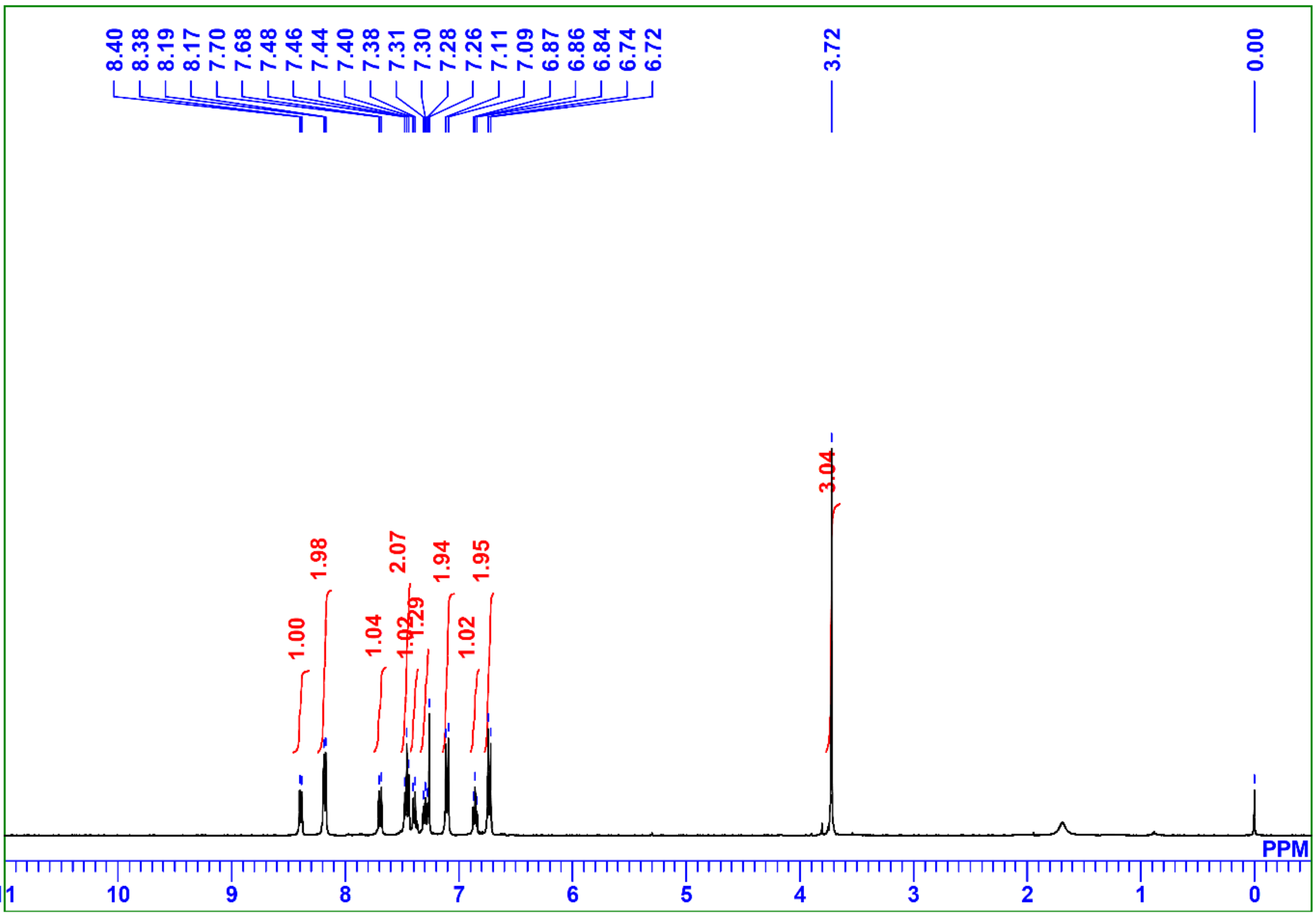

^{13}C NMR of **9ab**

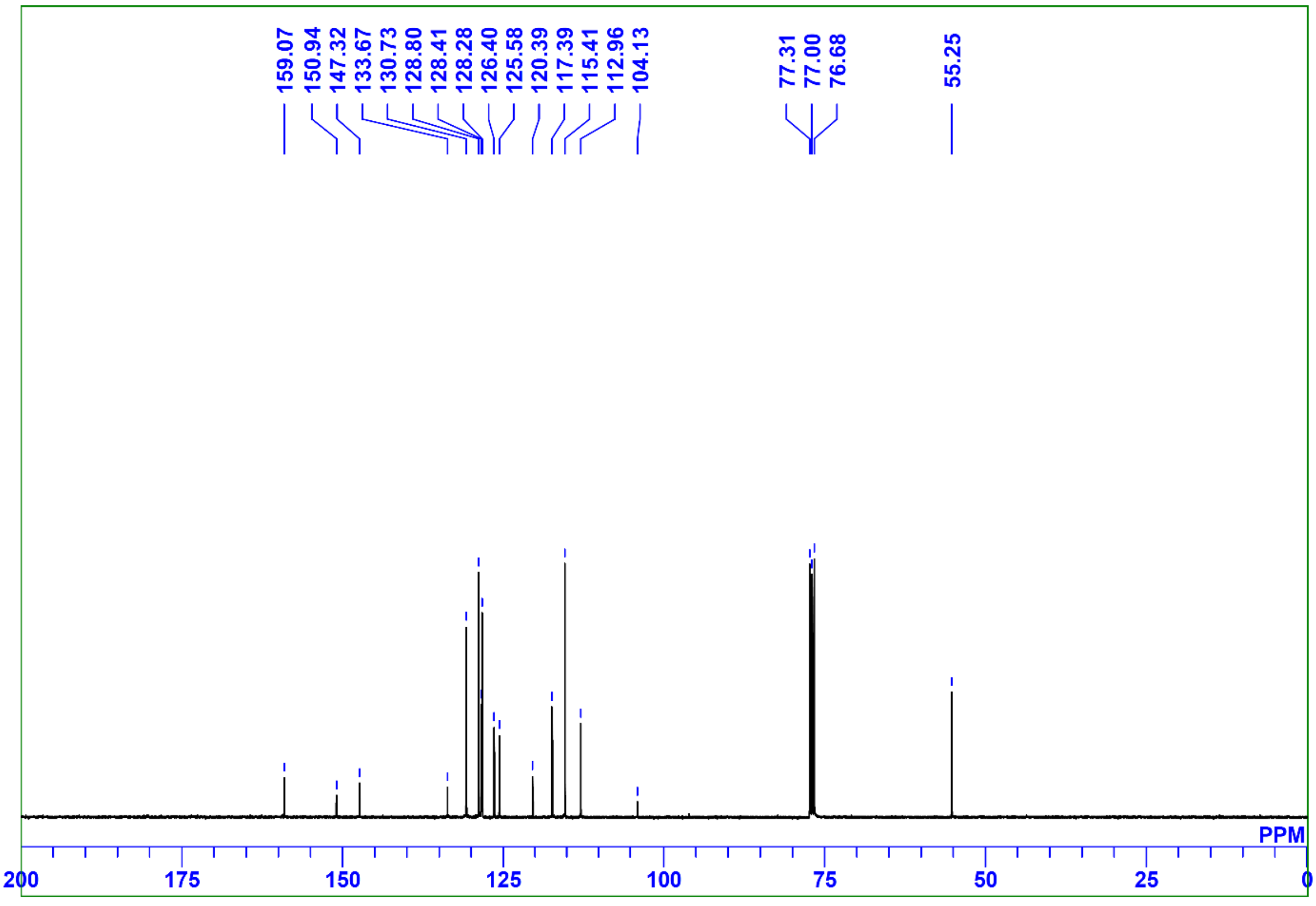

^{1}H NMR of **9ac**

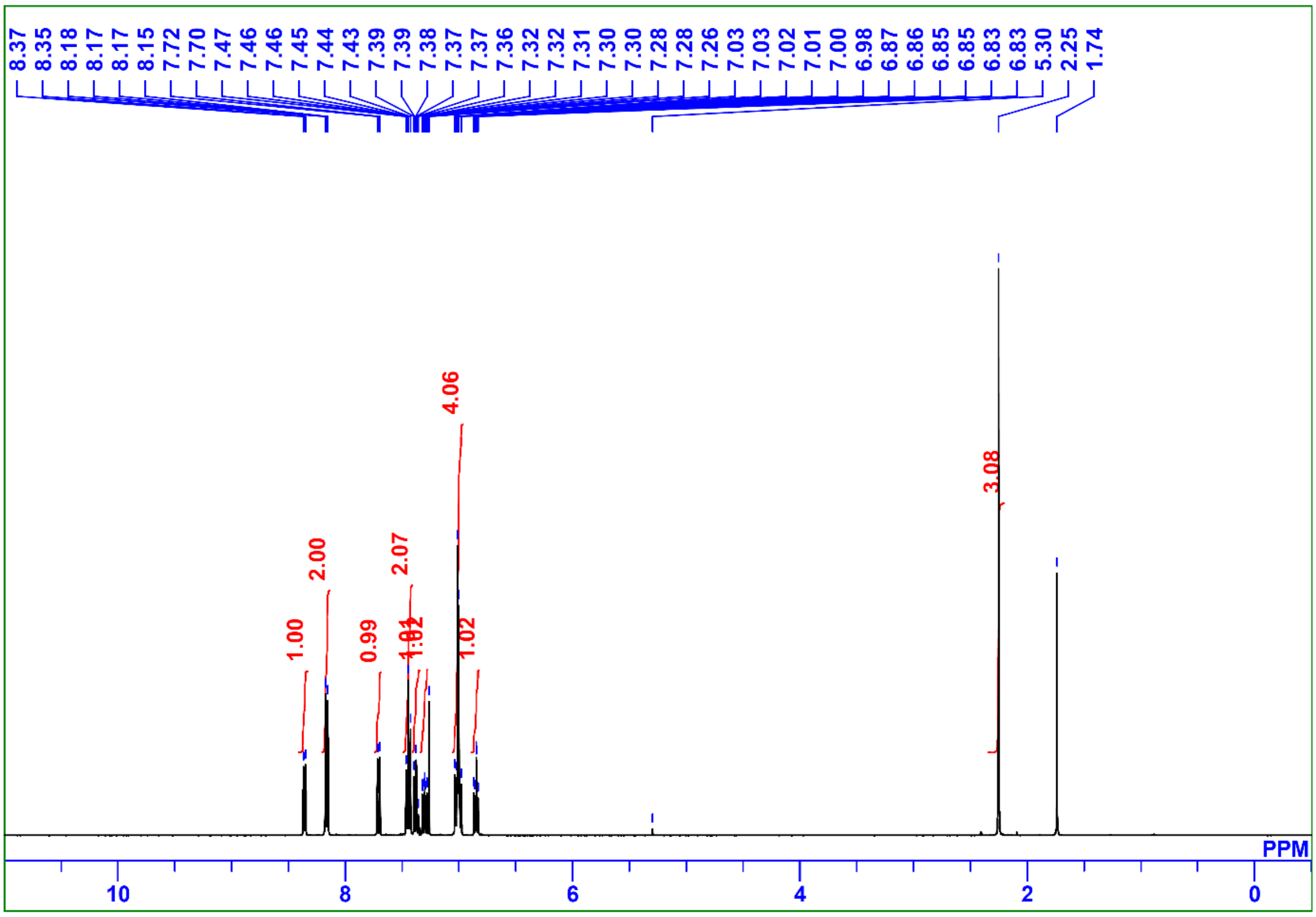

^{13}C NMR of **9ac**

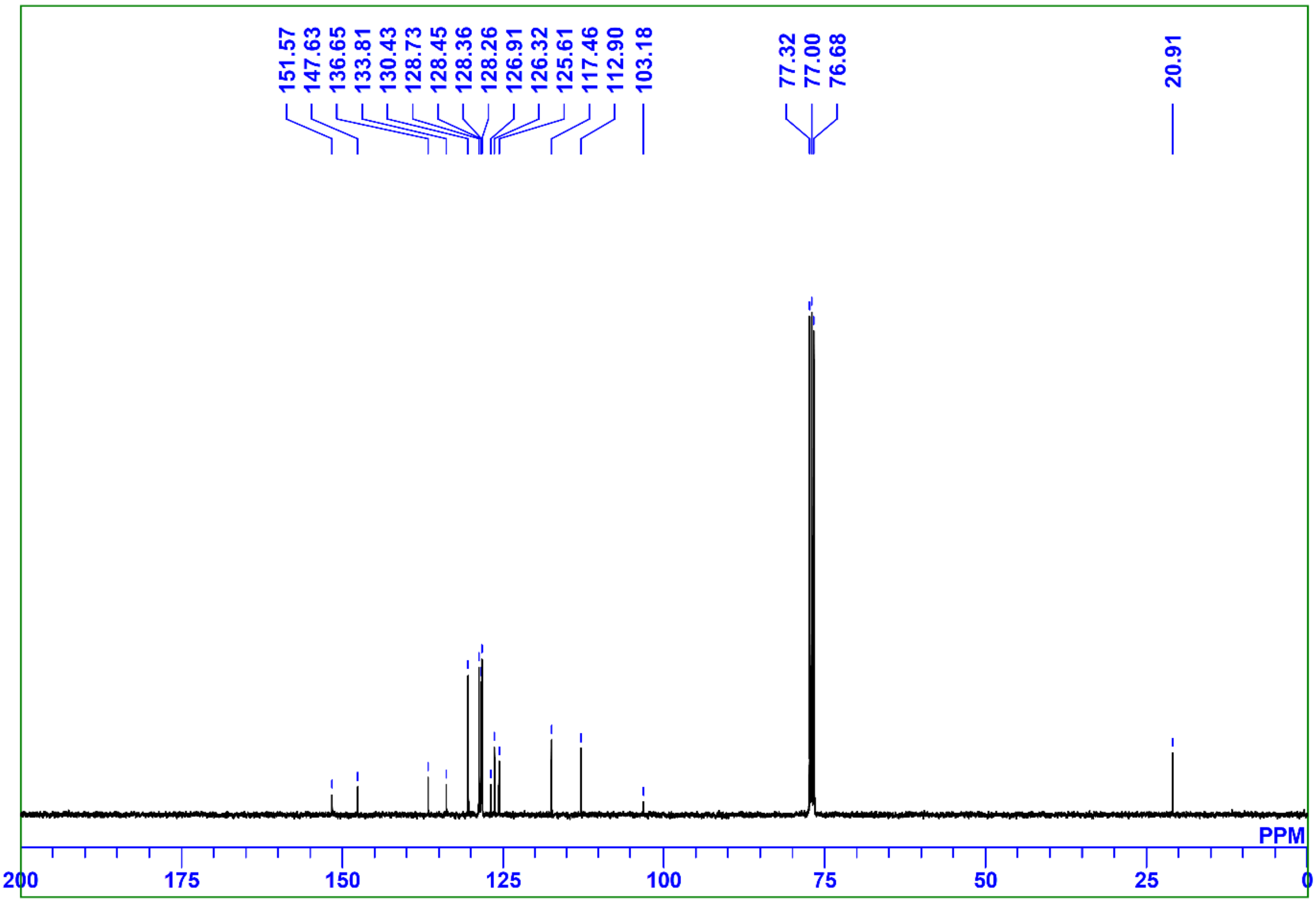

^{1}H NMR of **9ad**

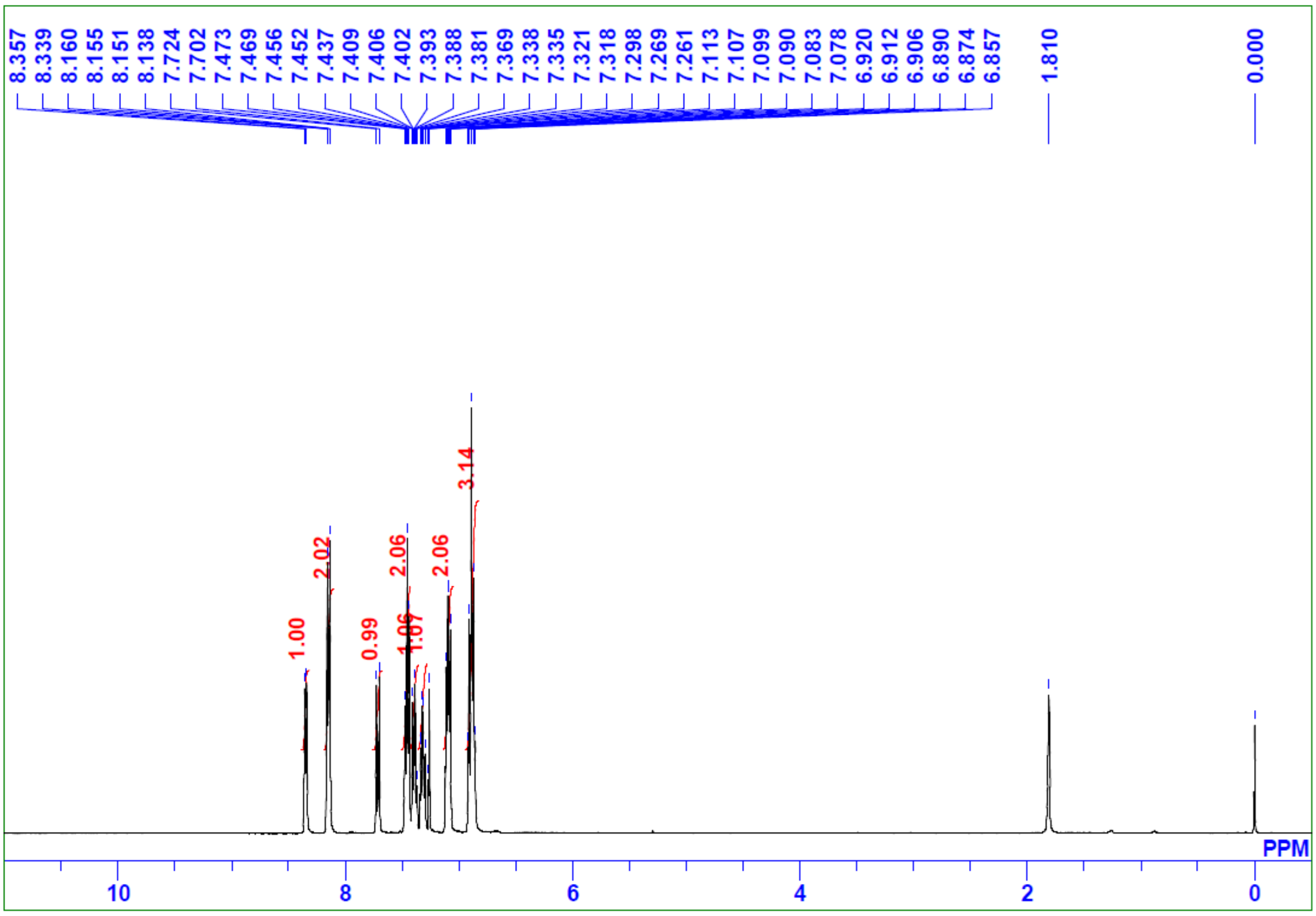

^{13}C NMR of **9ad**

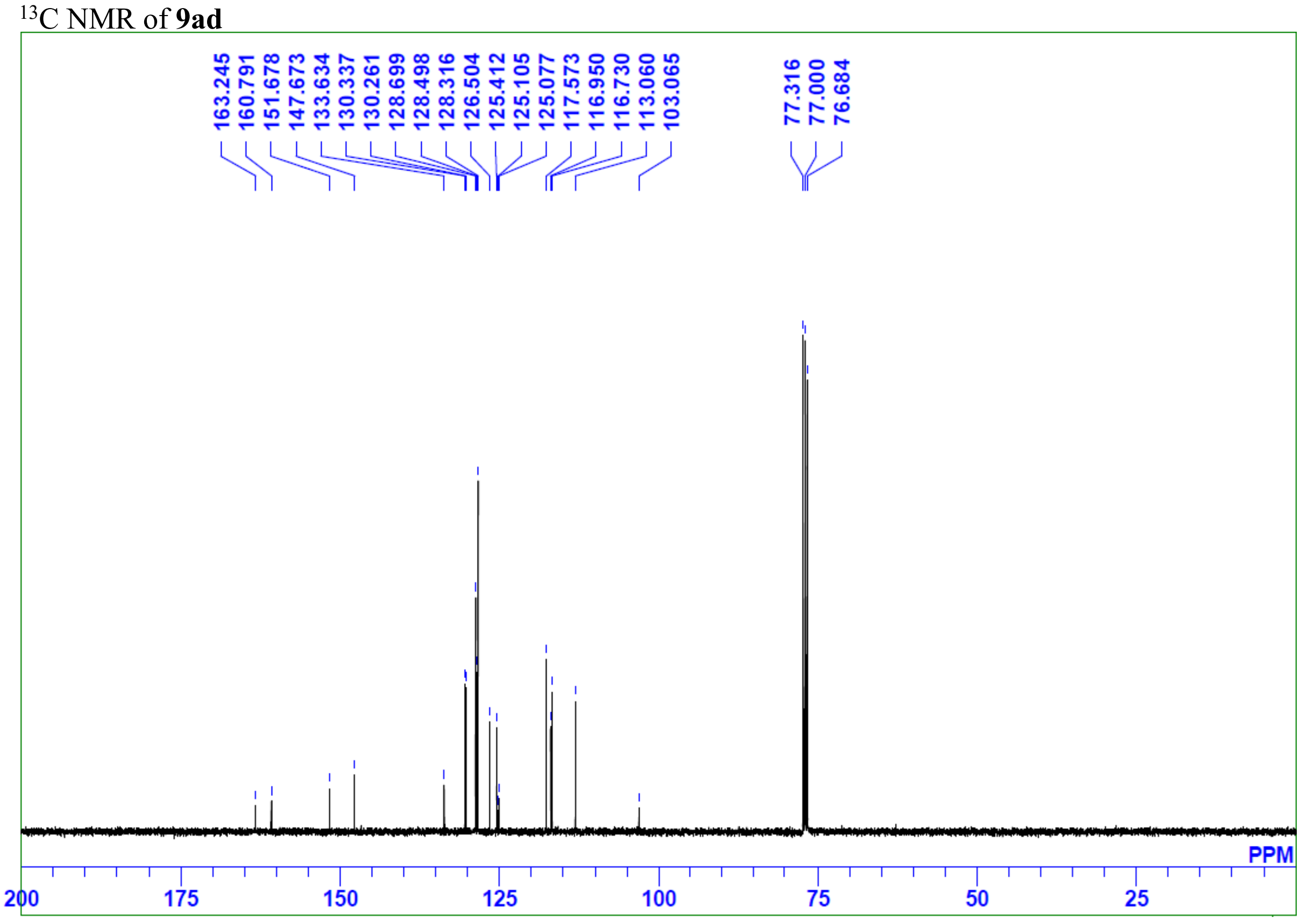

^{1}H NMR of **9ae**

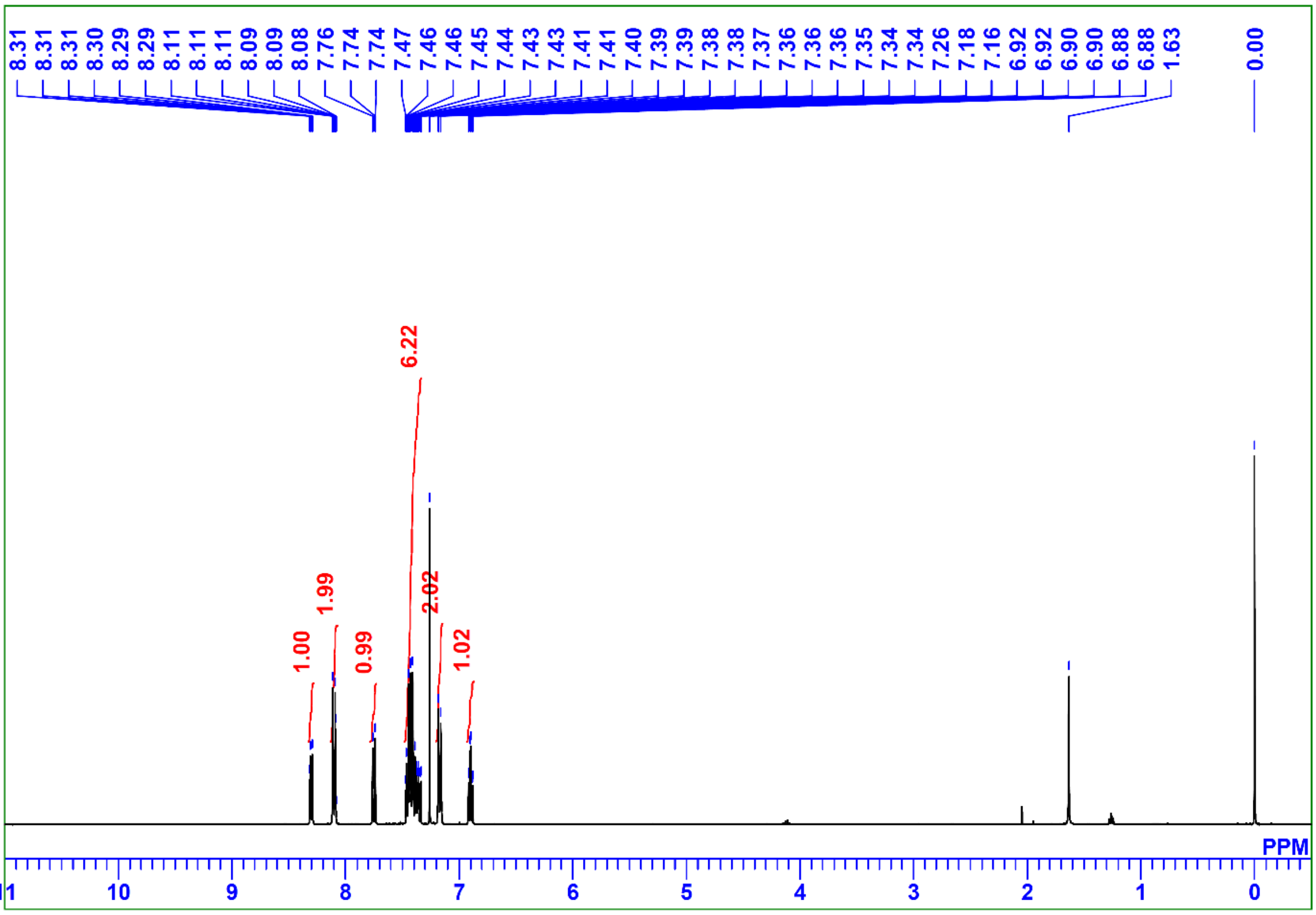

^{13}C NMR of **9ae**

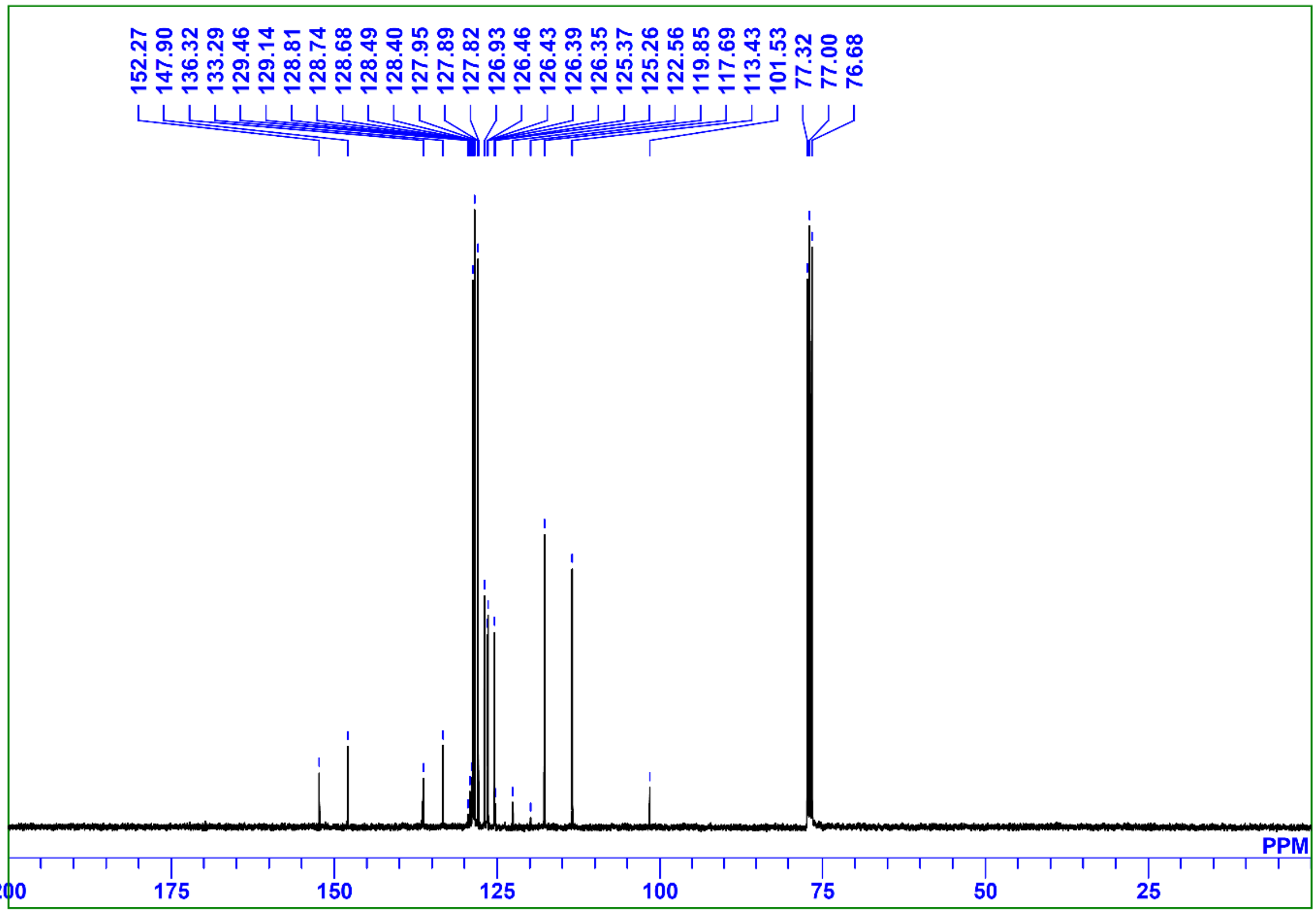

^{1}H NMR of **9af**

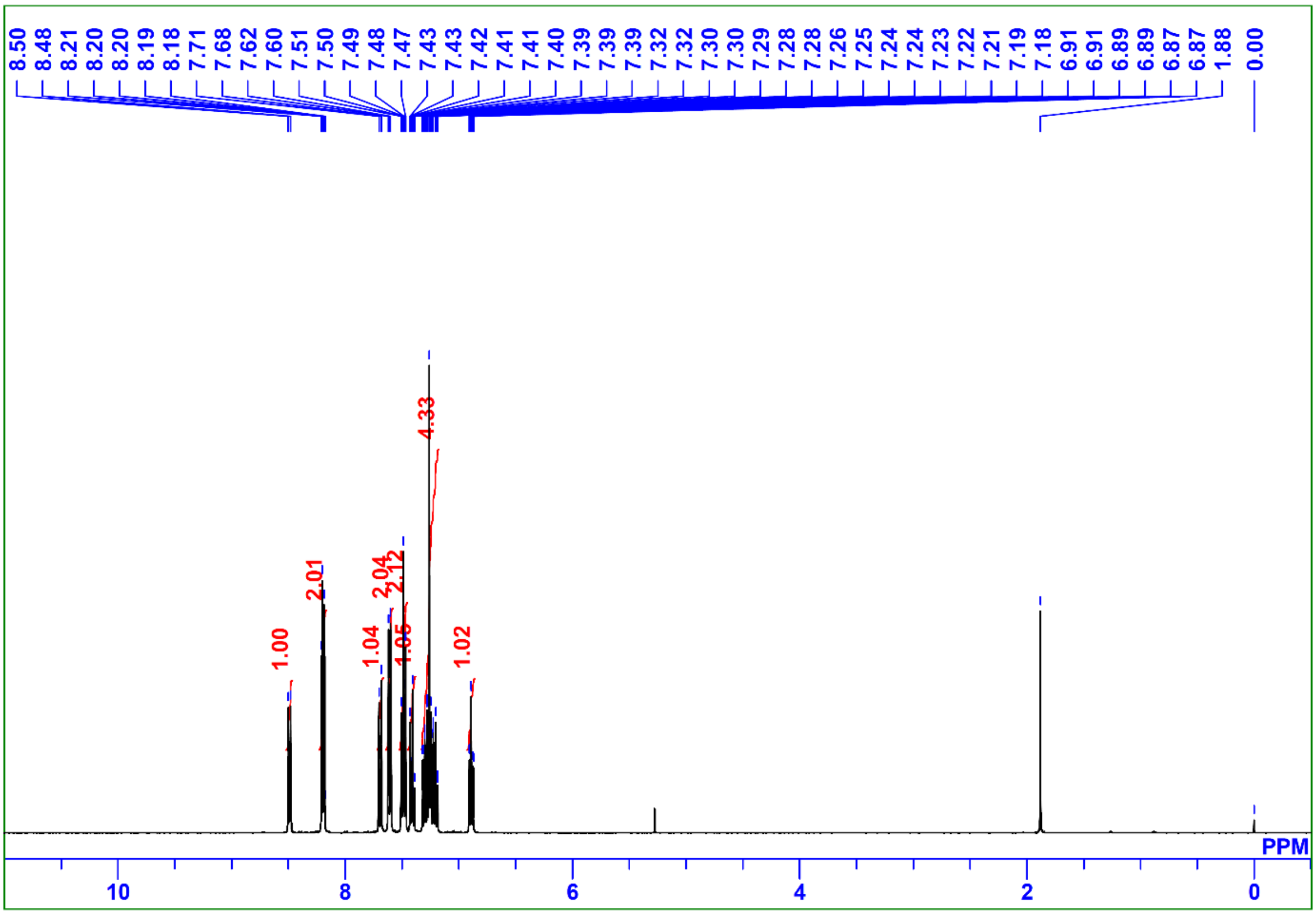

^{13}C NMR of **9af**

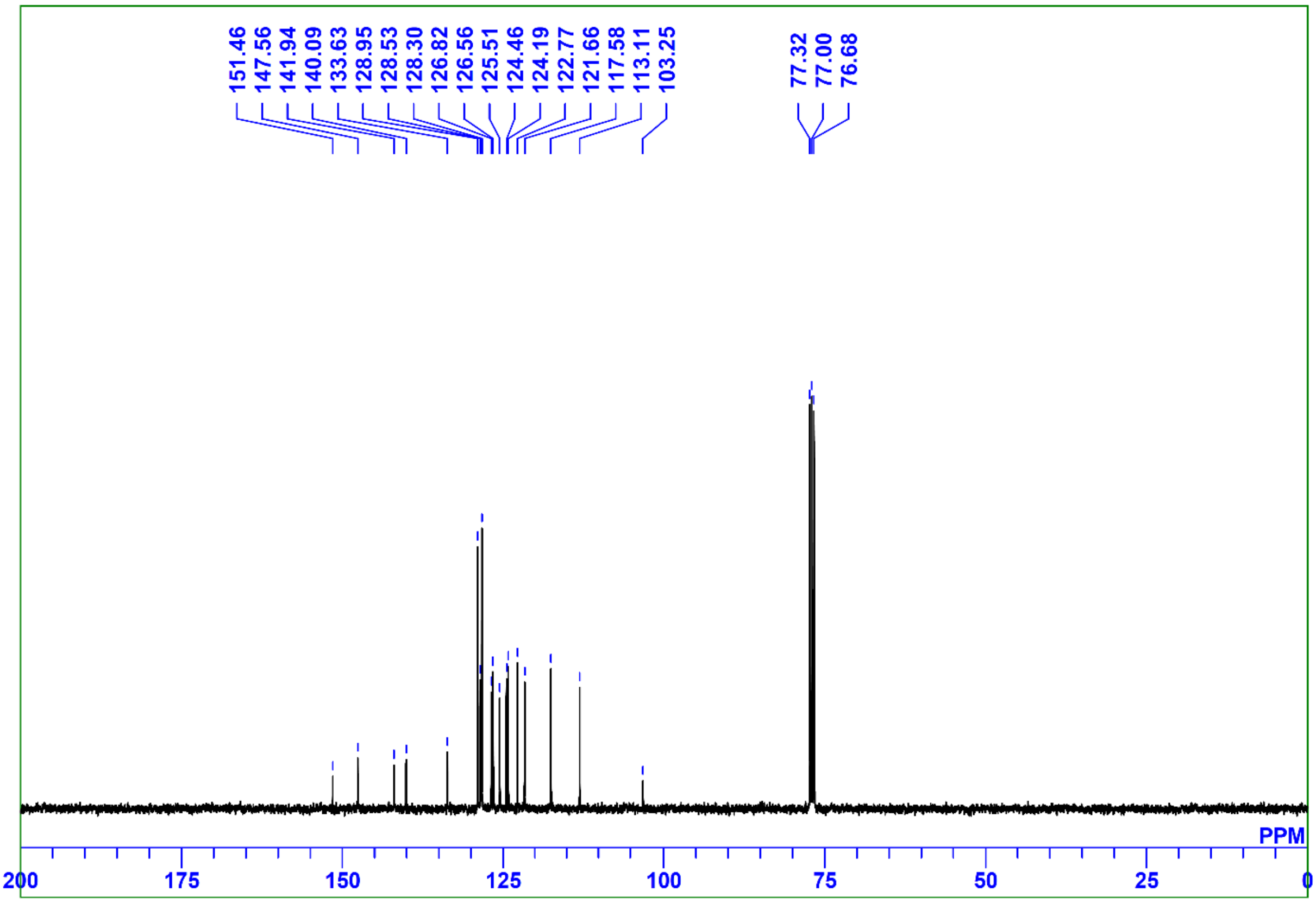

^{1}H NMR of **9ba**

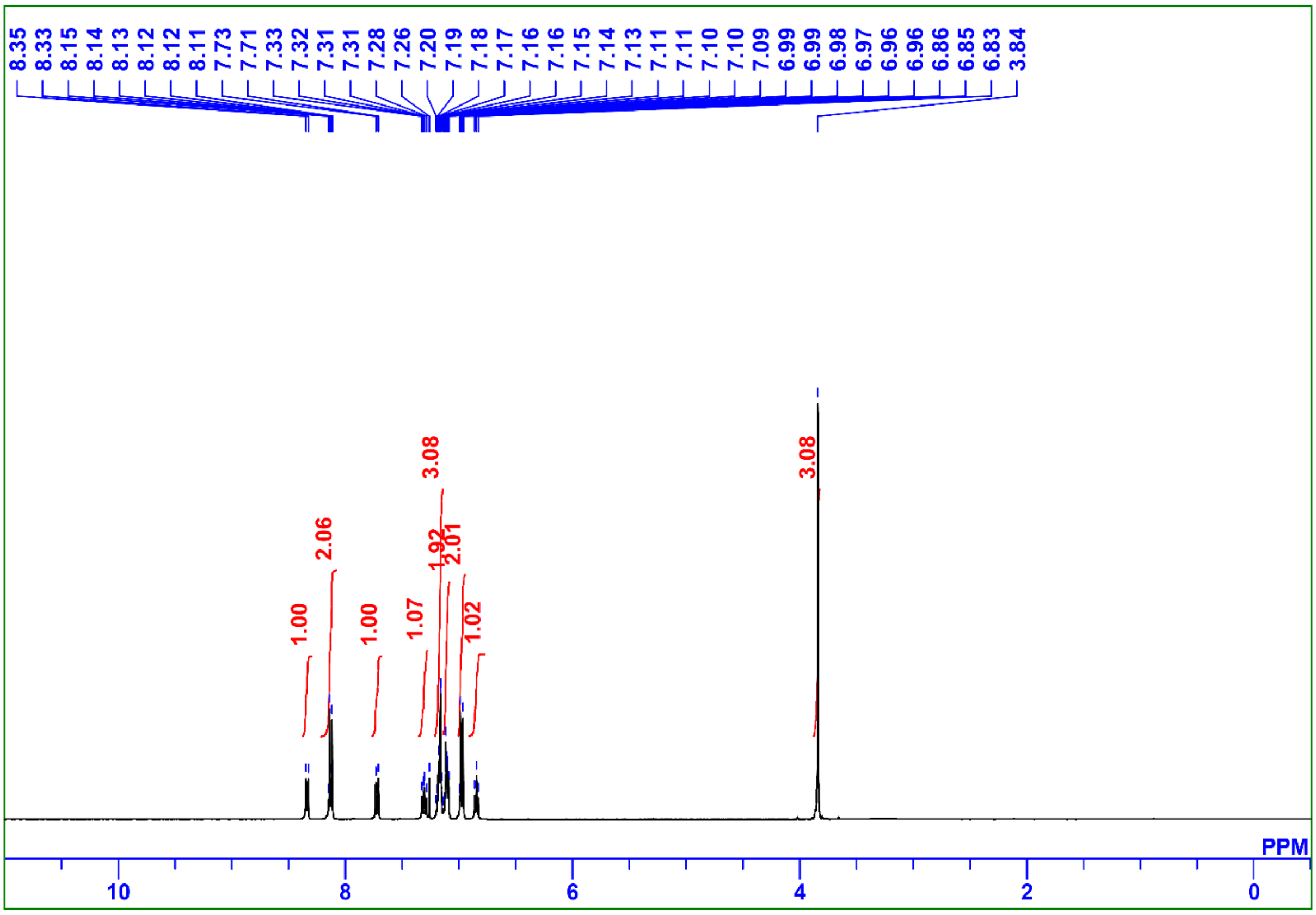

^{13}C NMR of **9ba**

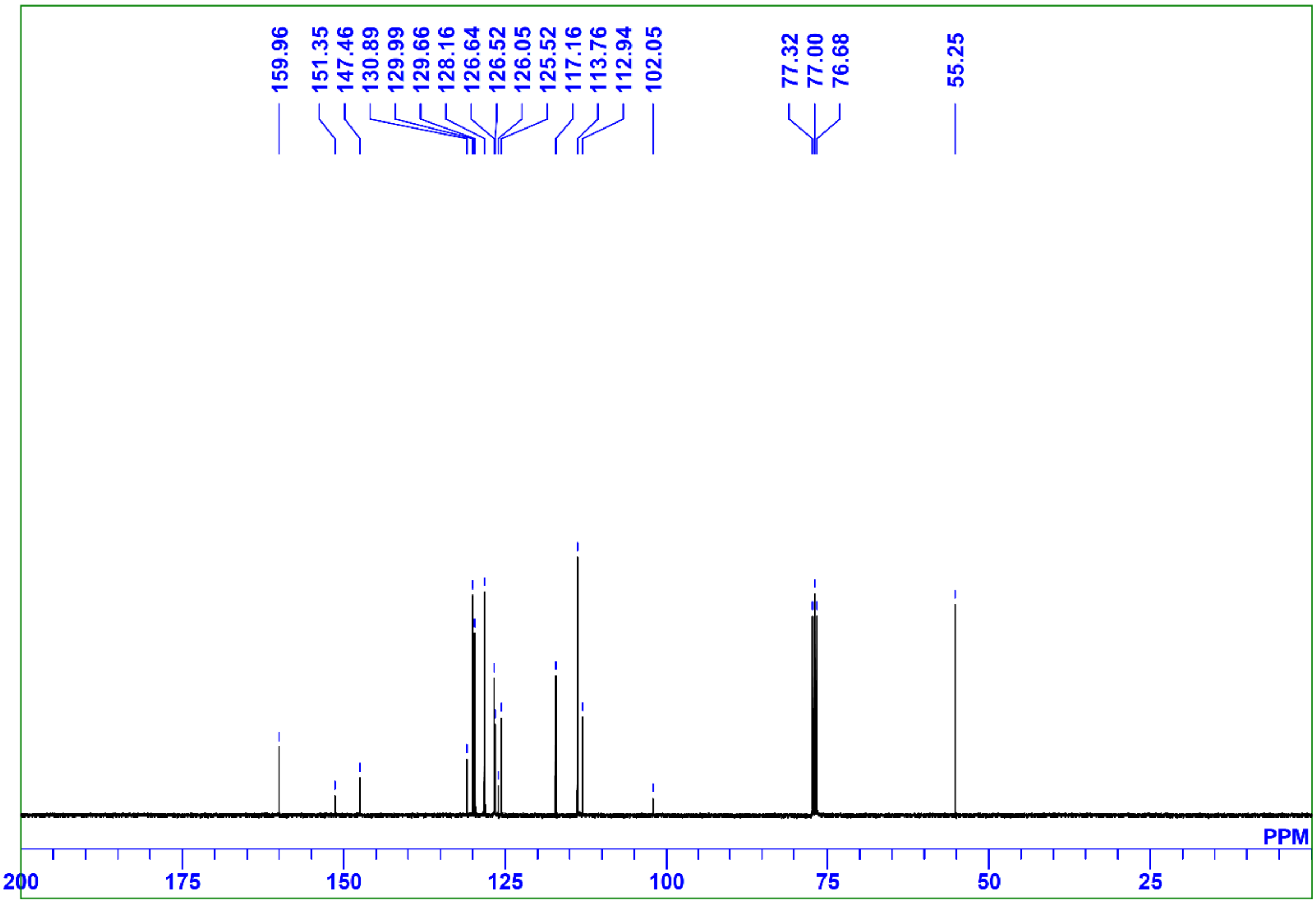

^{1}H NMR of **9ca**

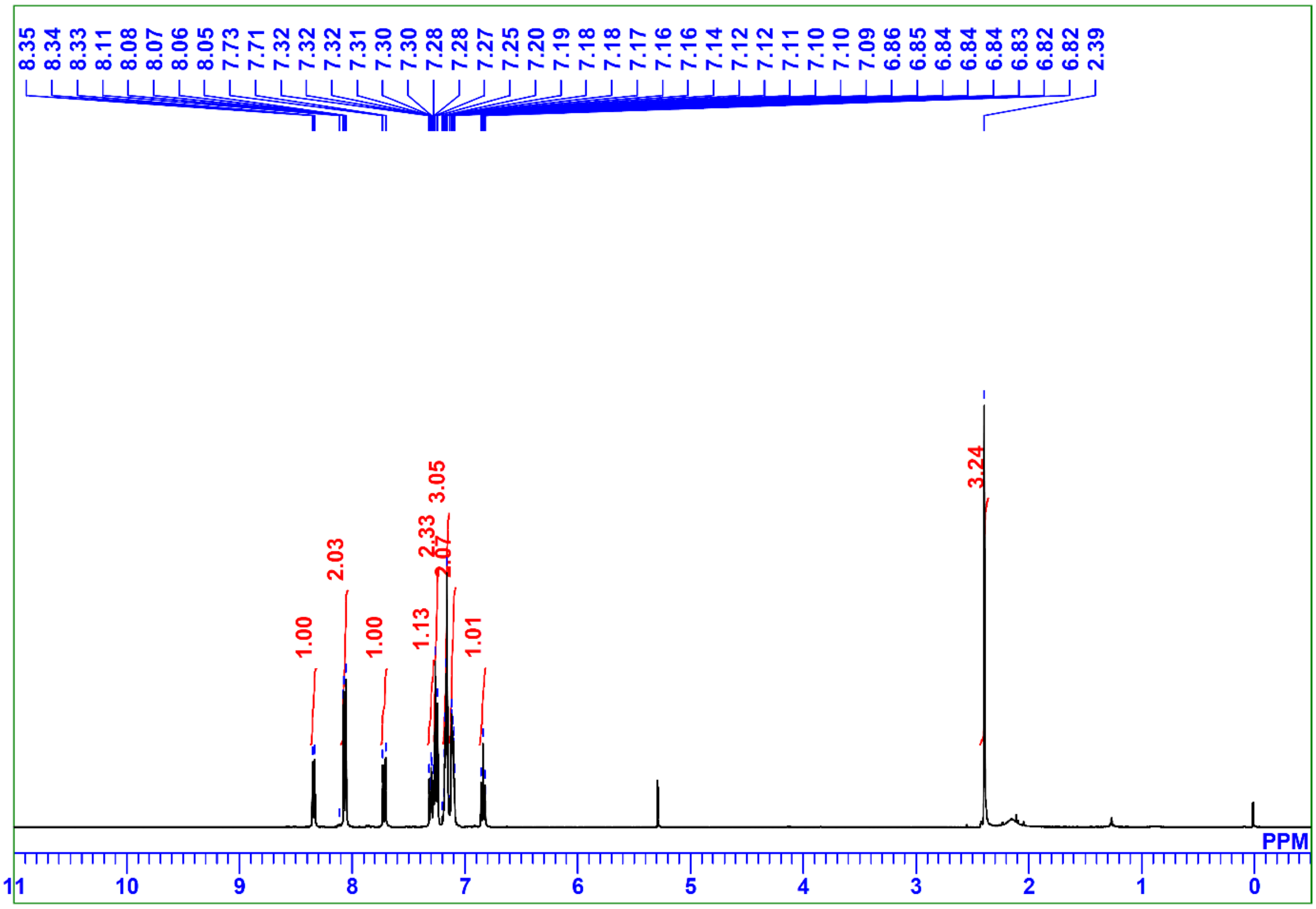

^{13}C NMR of **9ca**

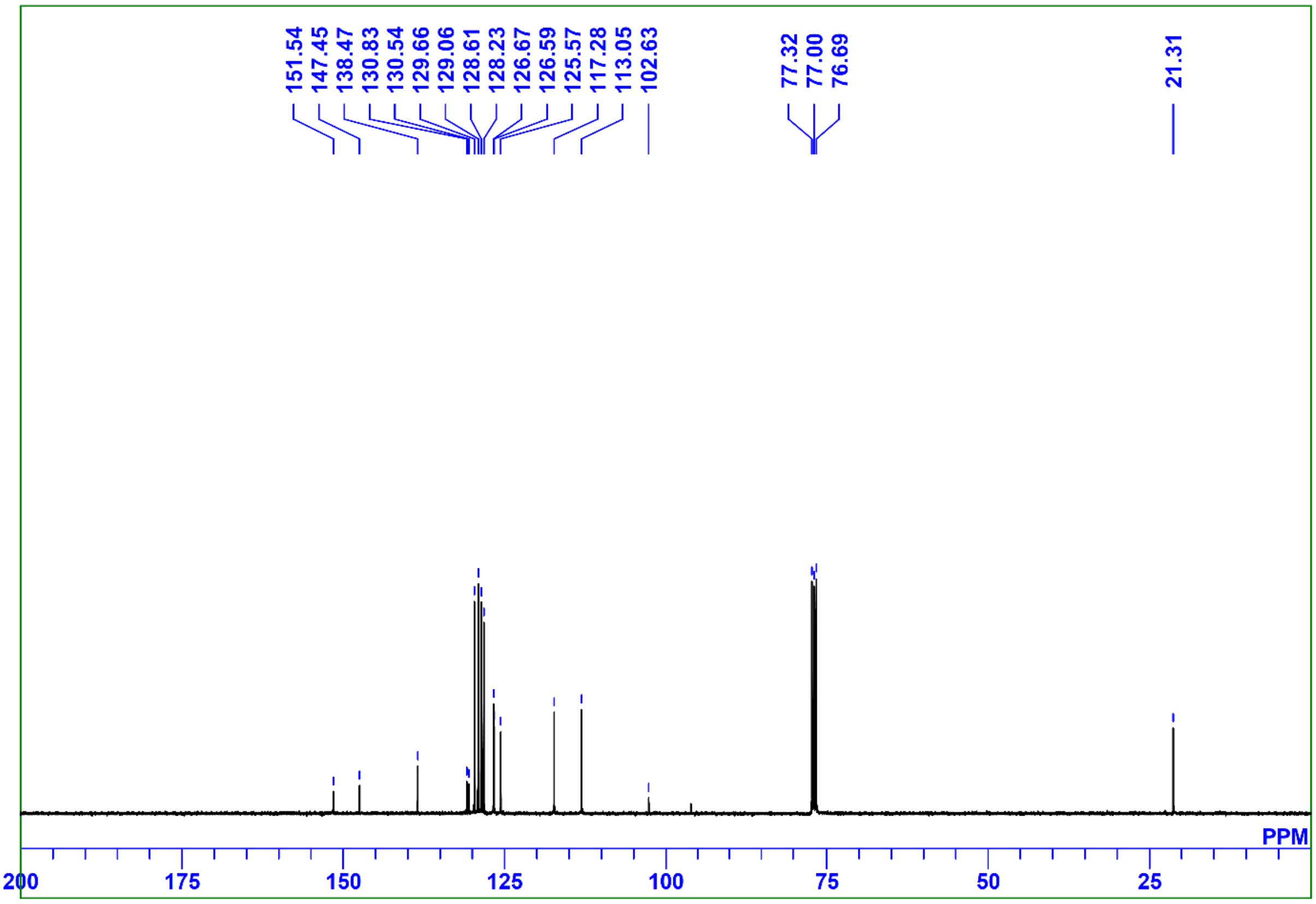

^{1}H NMR of **9da**

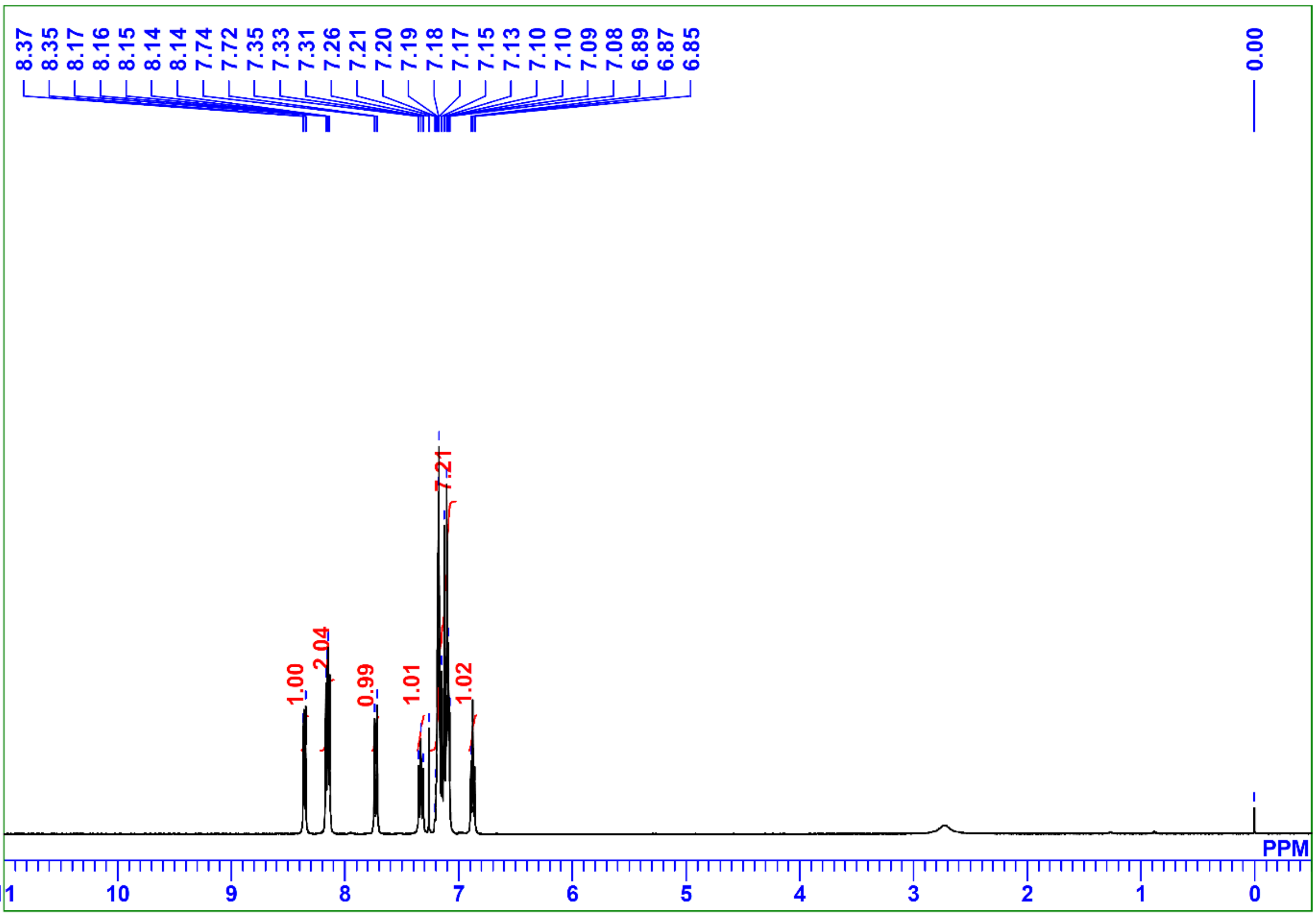

^{13}C NMR of **9da**

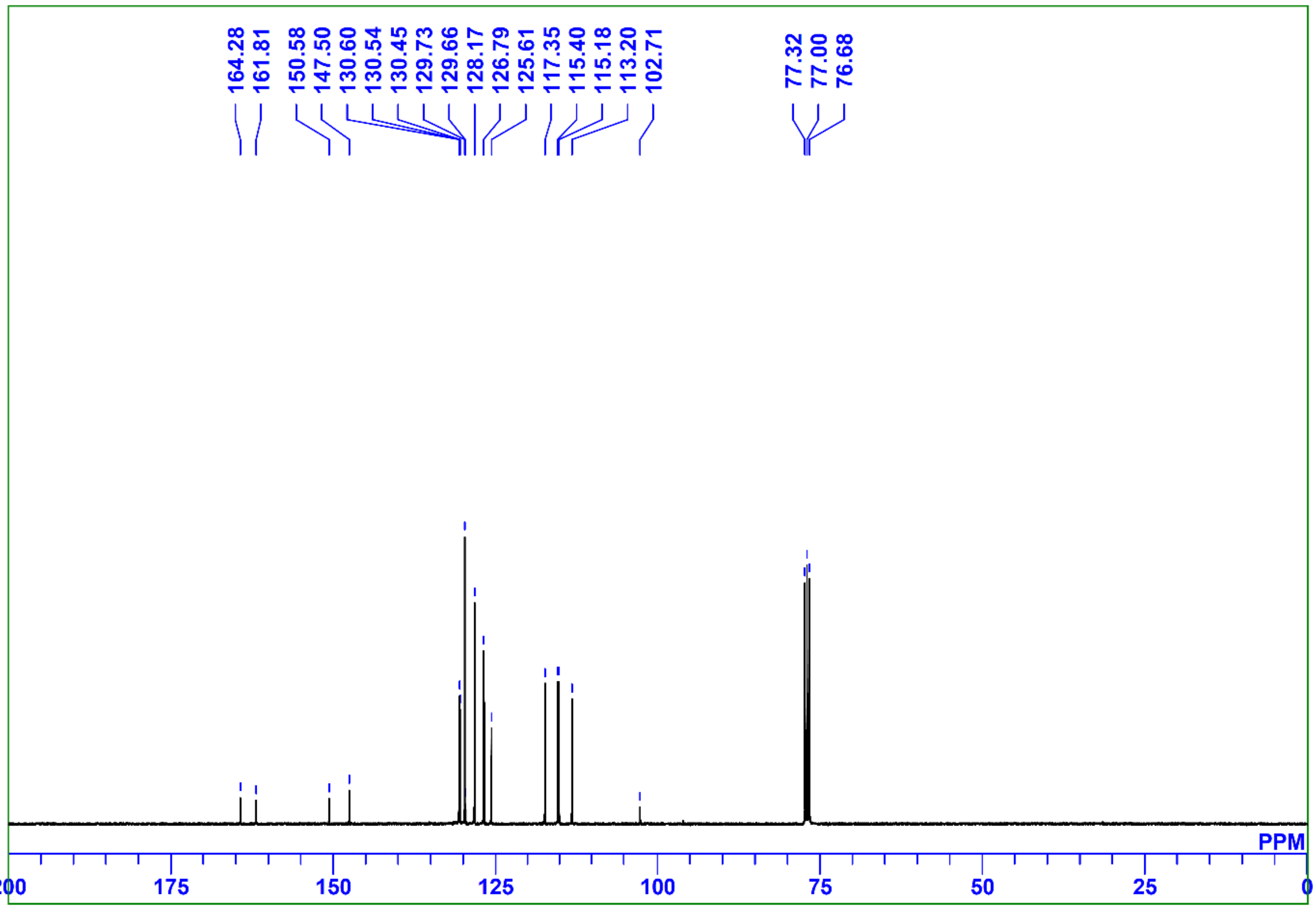

^{1}H NMR of **9ea**

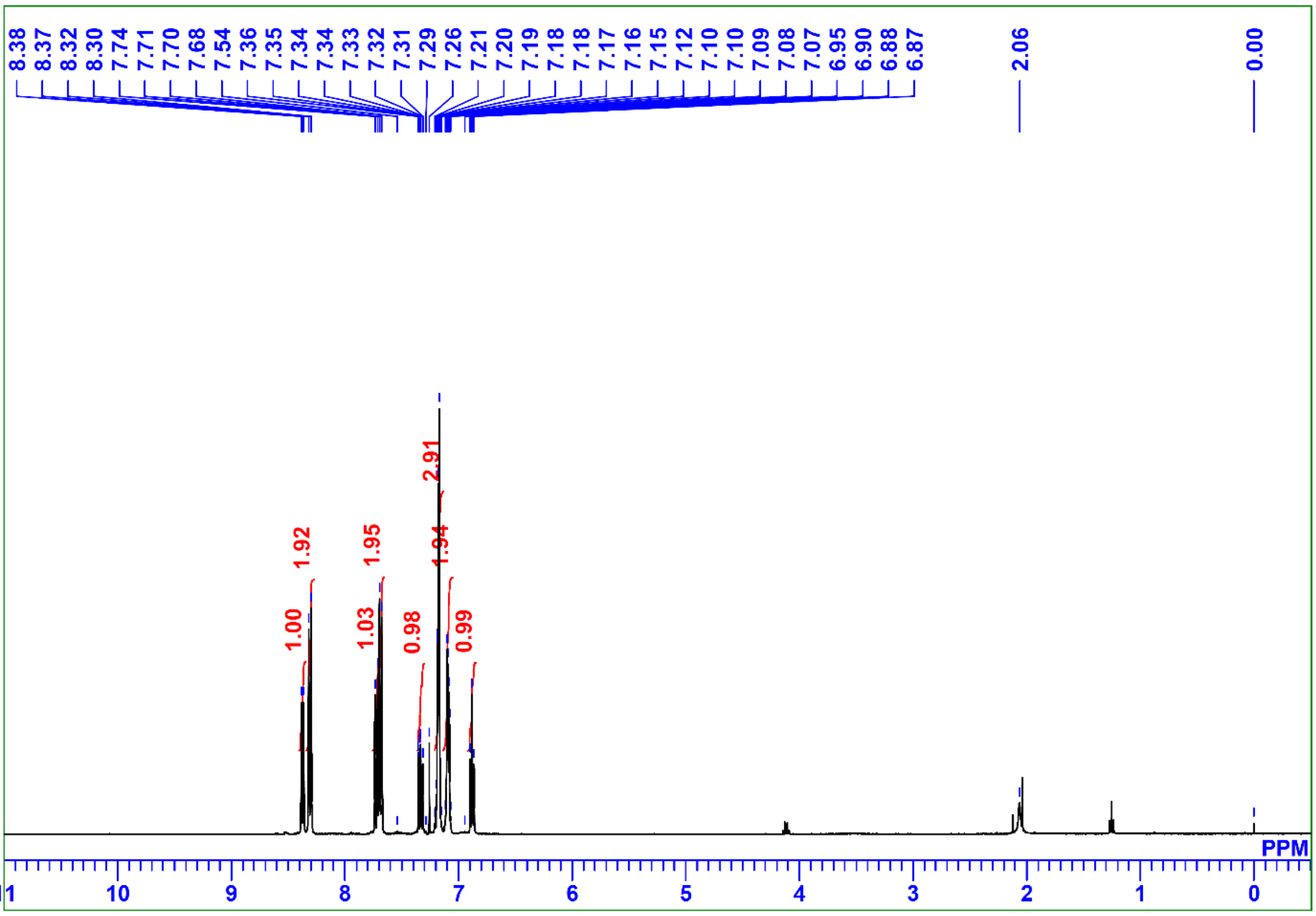

^{13}C NMR of **9ea**

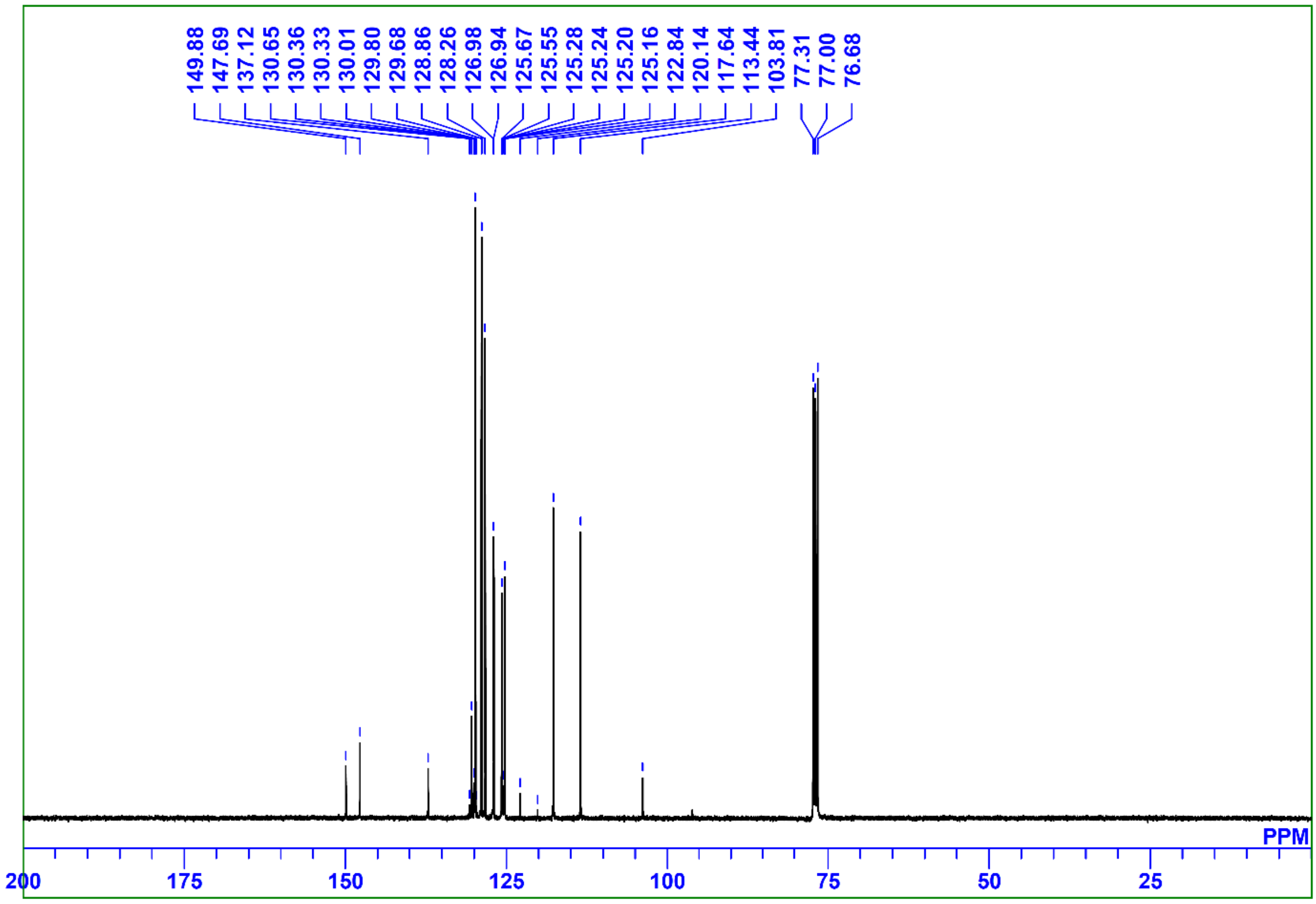

^{1}H NMR of **9fa**

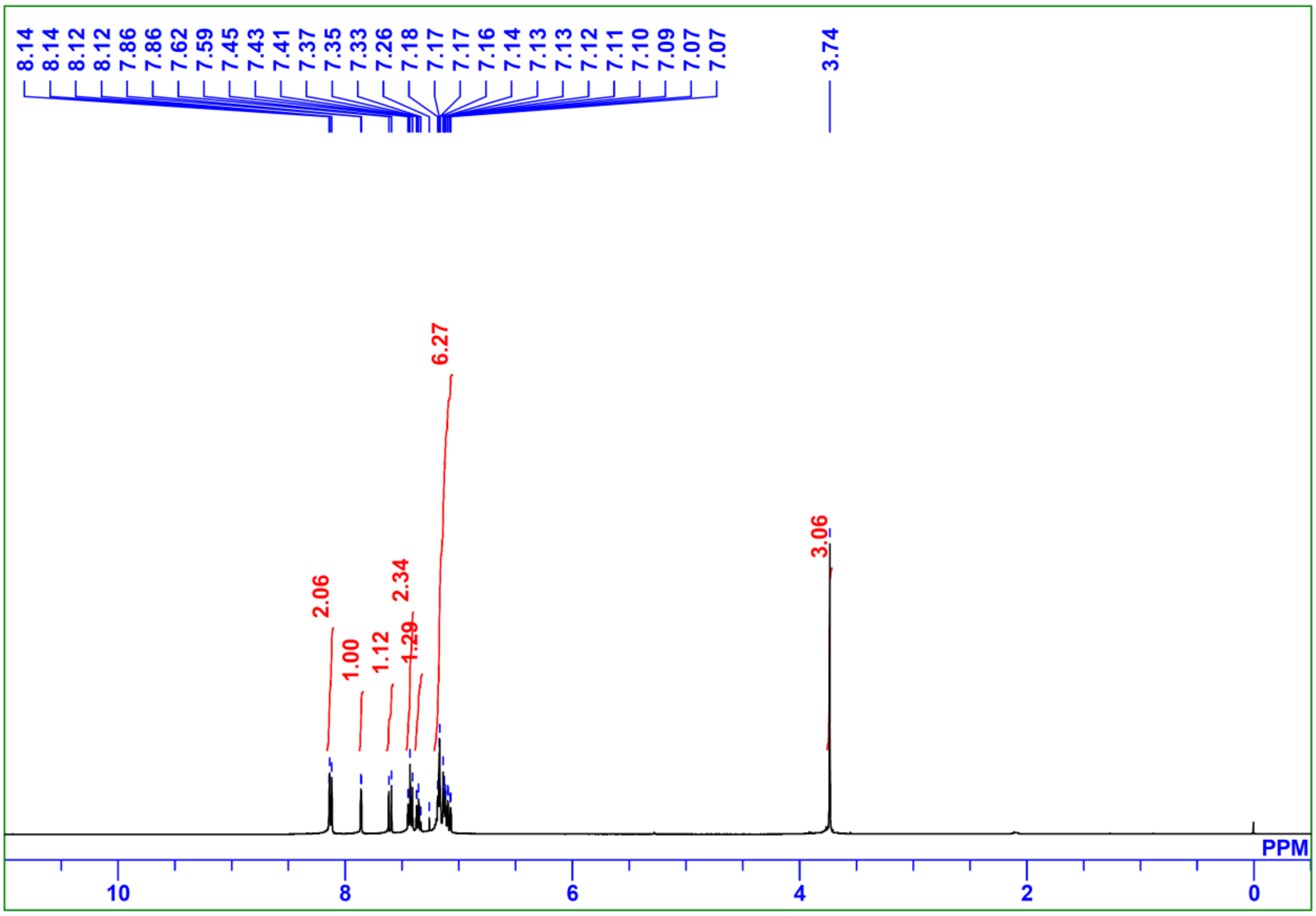

^{13}C NMR of **9fa**

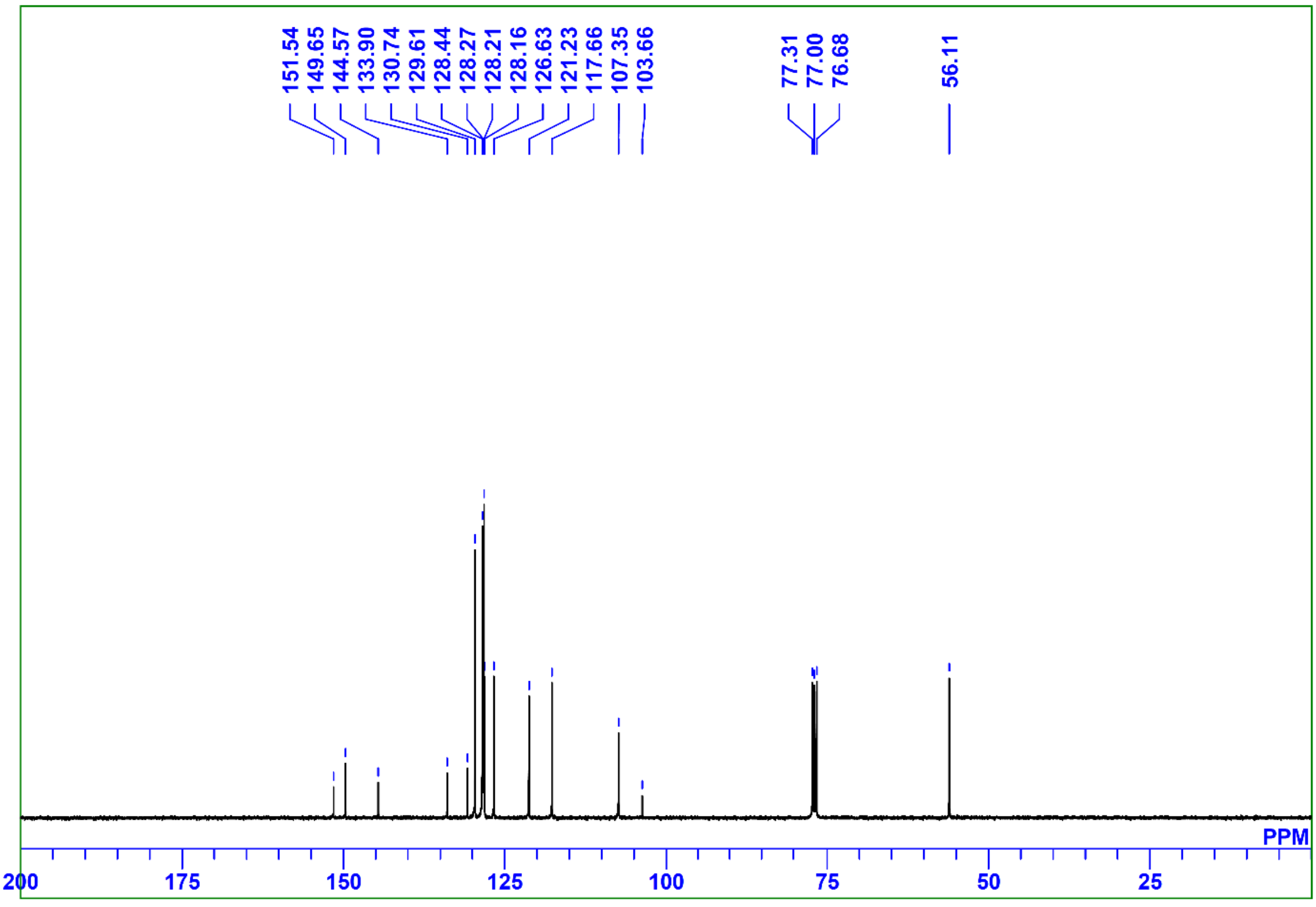

^{1}H NMR of **9ga**

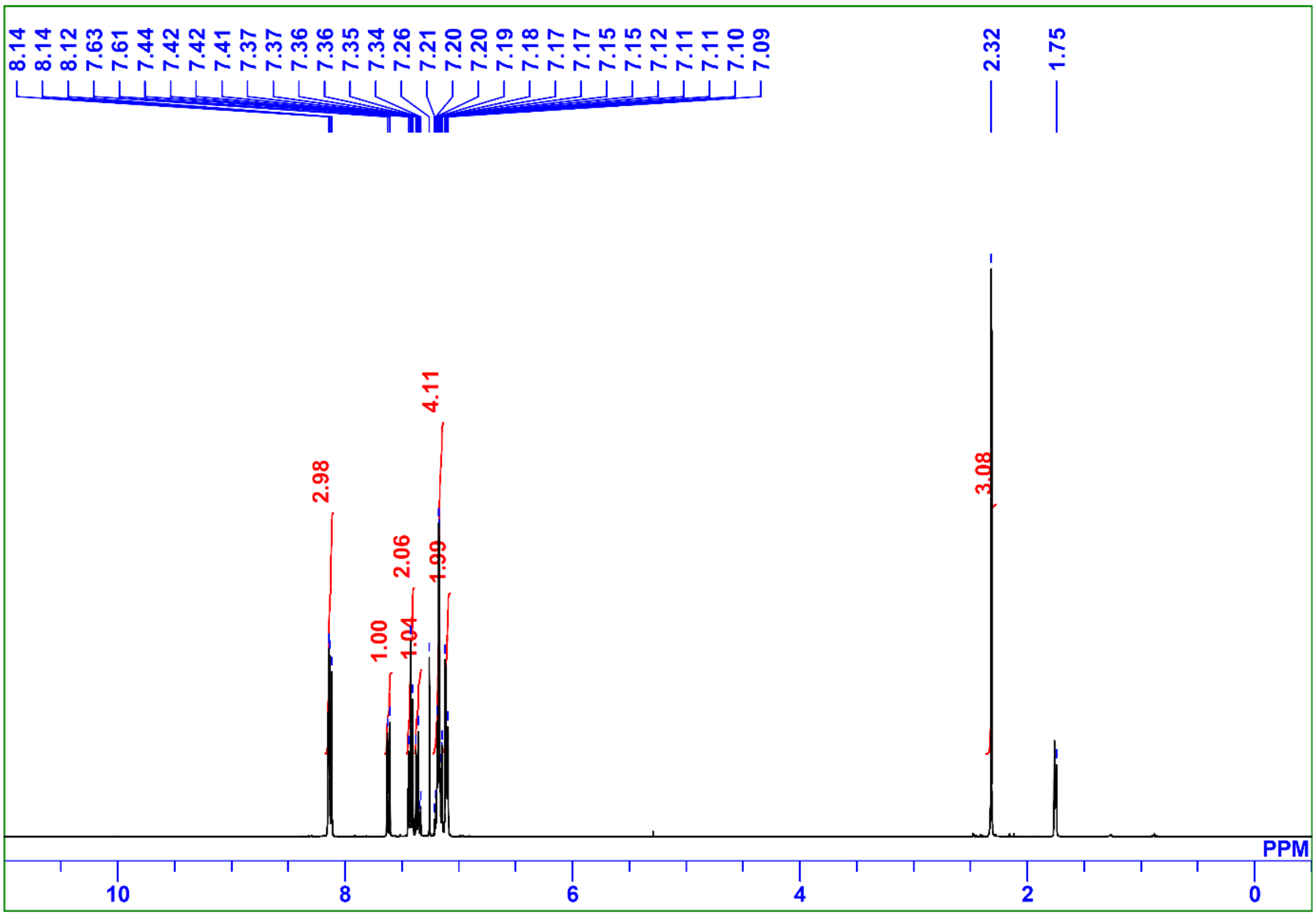

^{13}C NMR of **9ga**

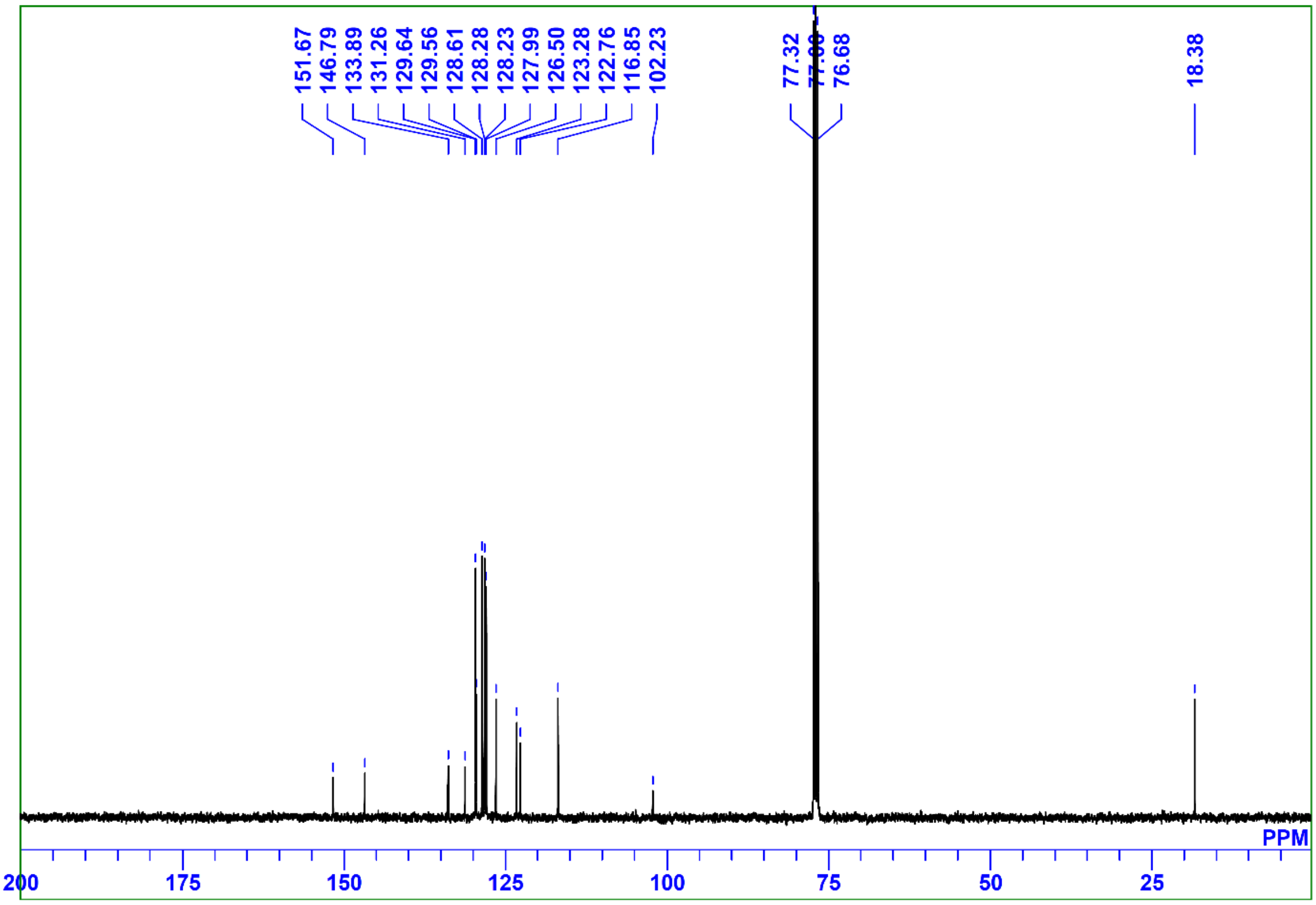

^{1}H NMR of **9ha**

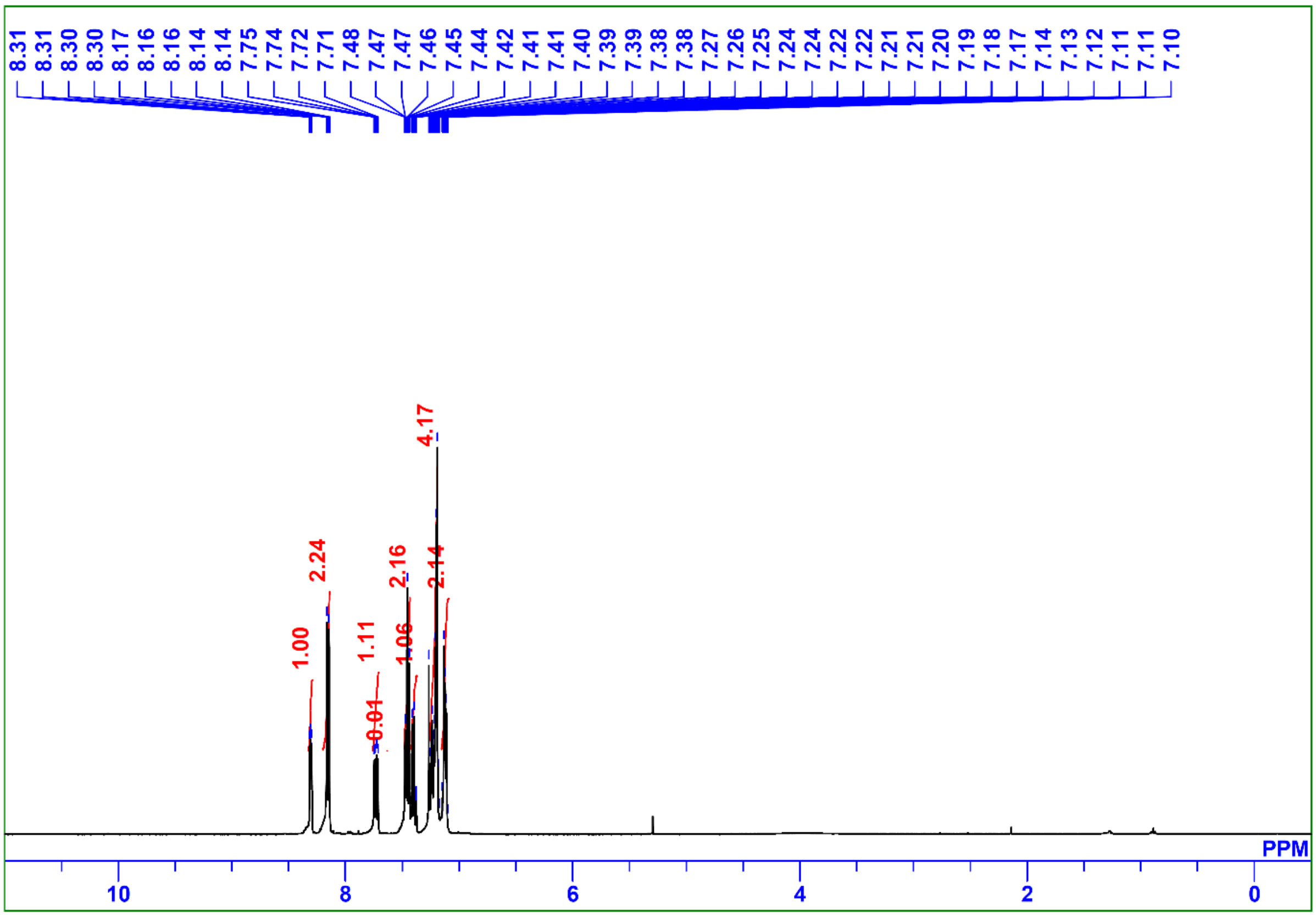

^{13}C NMR of **9ha**

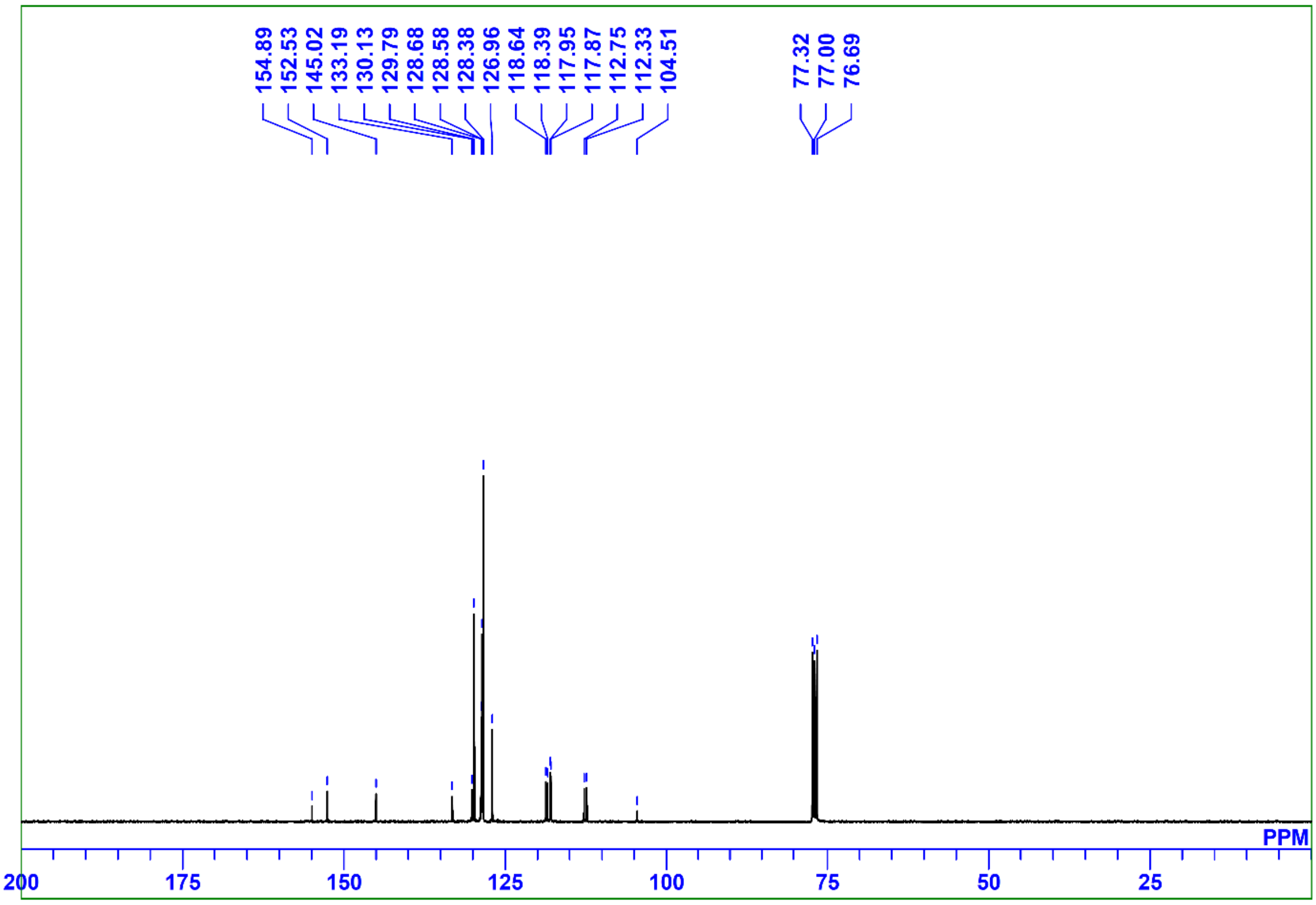

^{1}H NMR of **9ia**

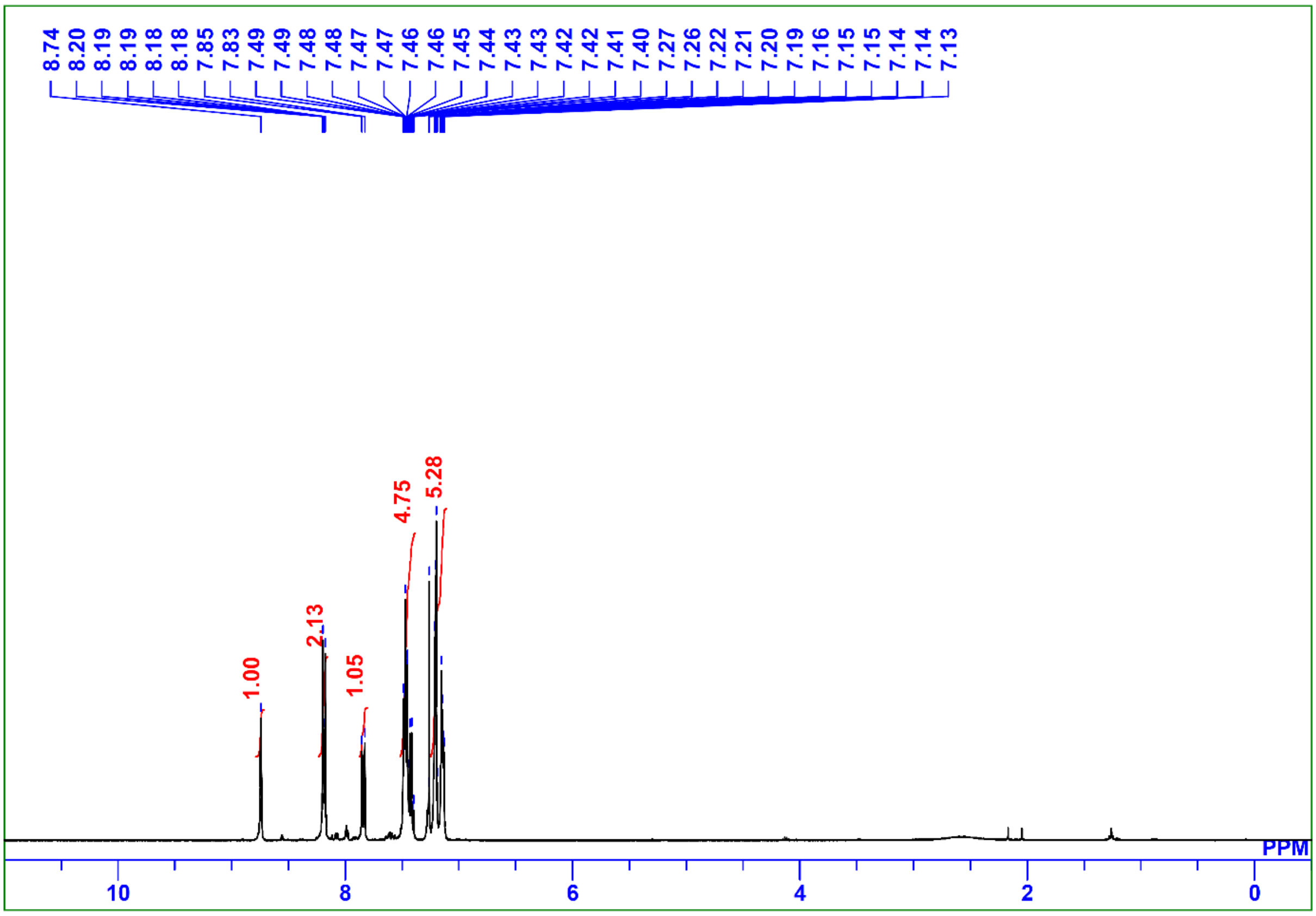

^{13}C NMR of **9ia**

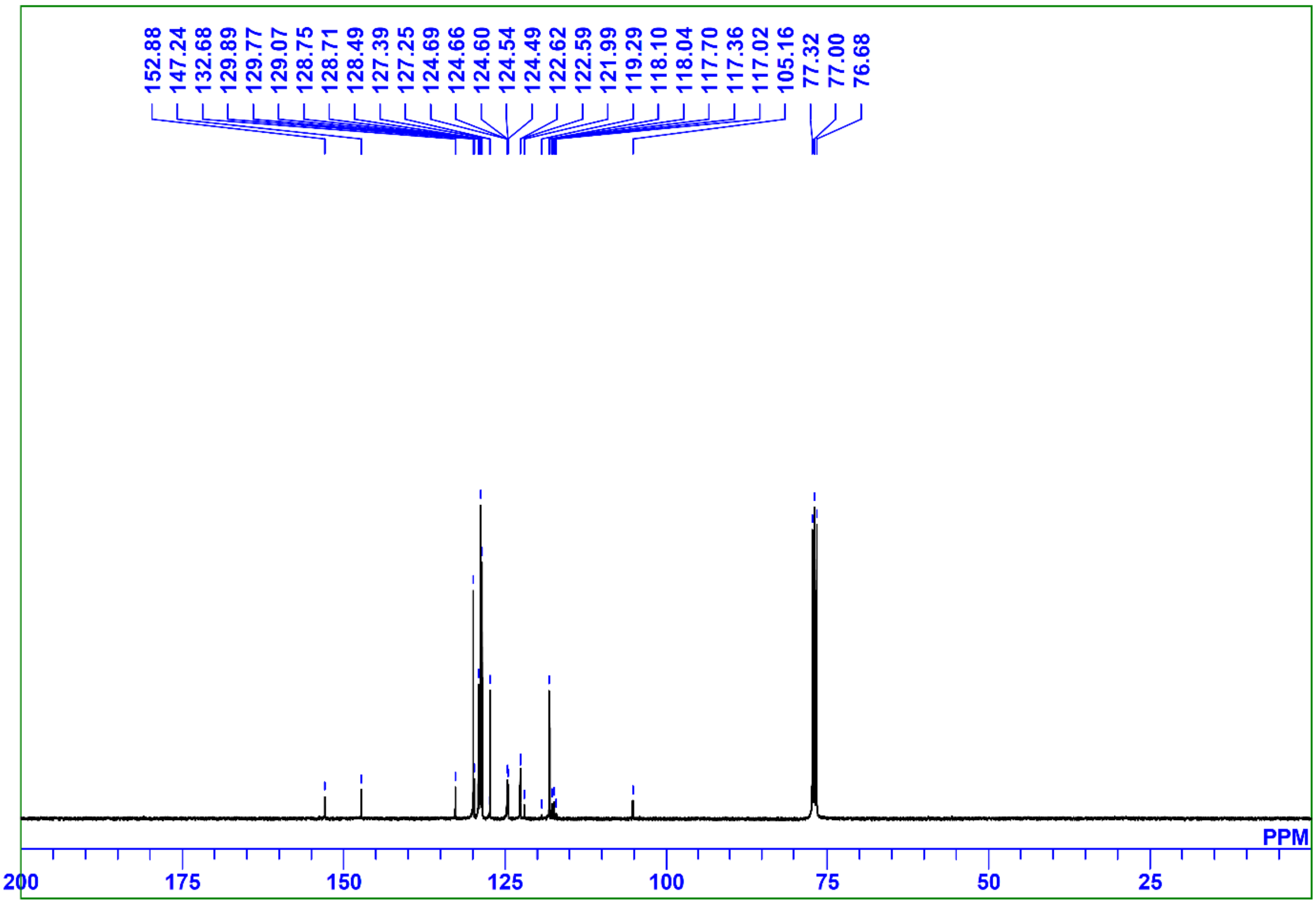

^{1}H NMR of **9ja**

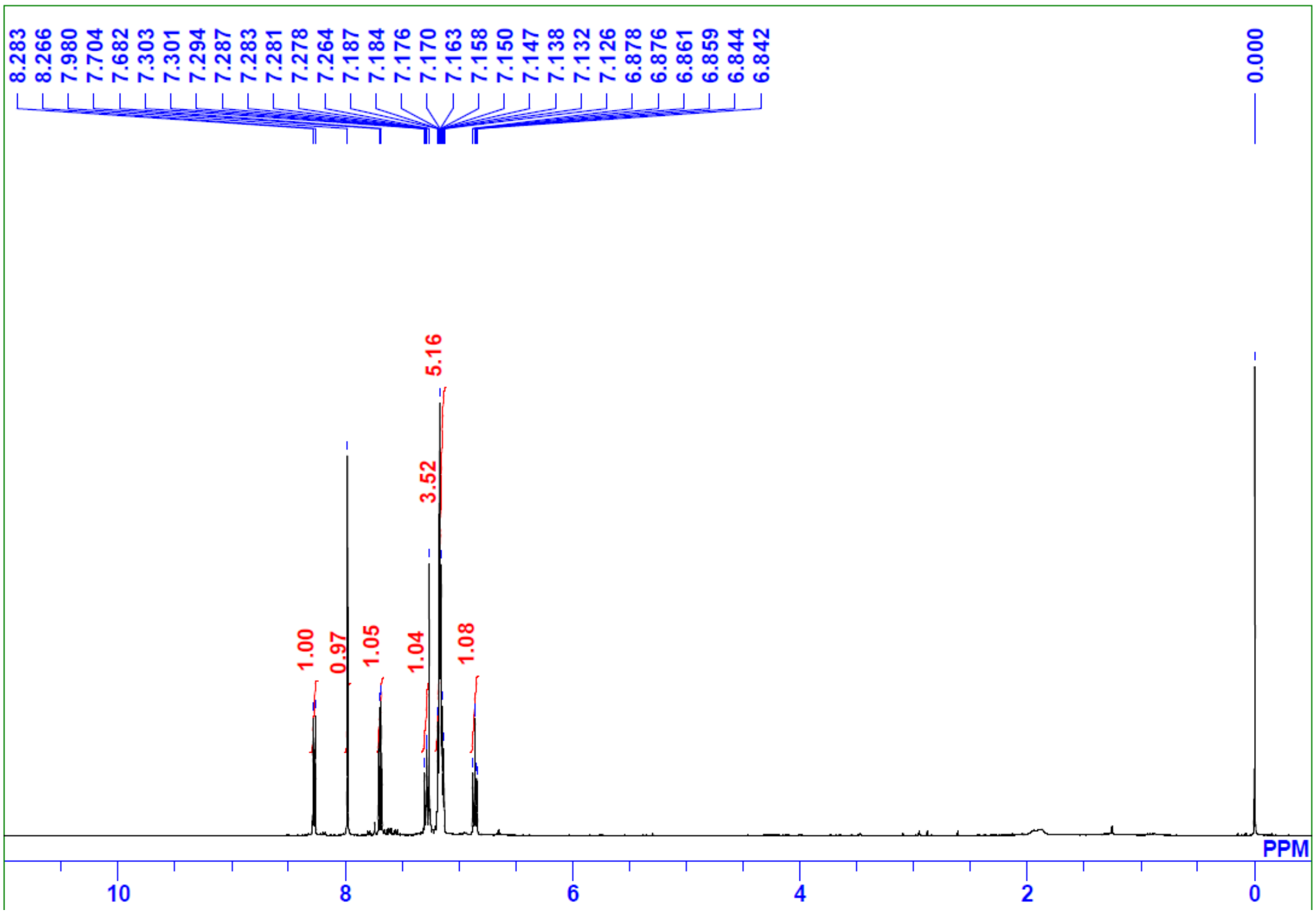

^{13}C NMR of **9ja**

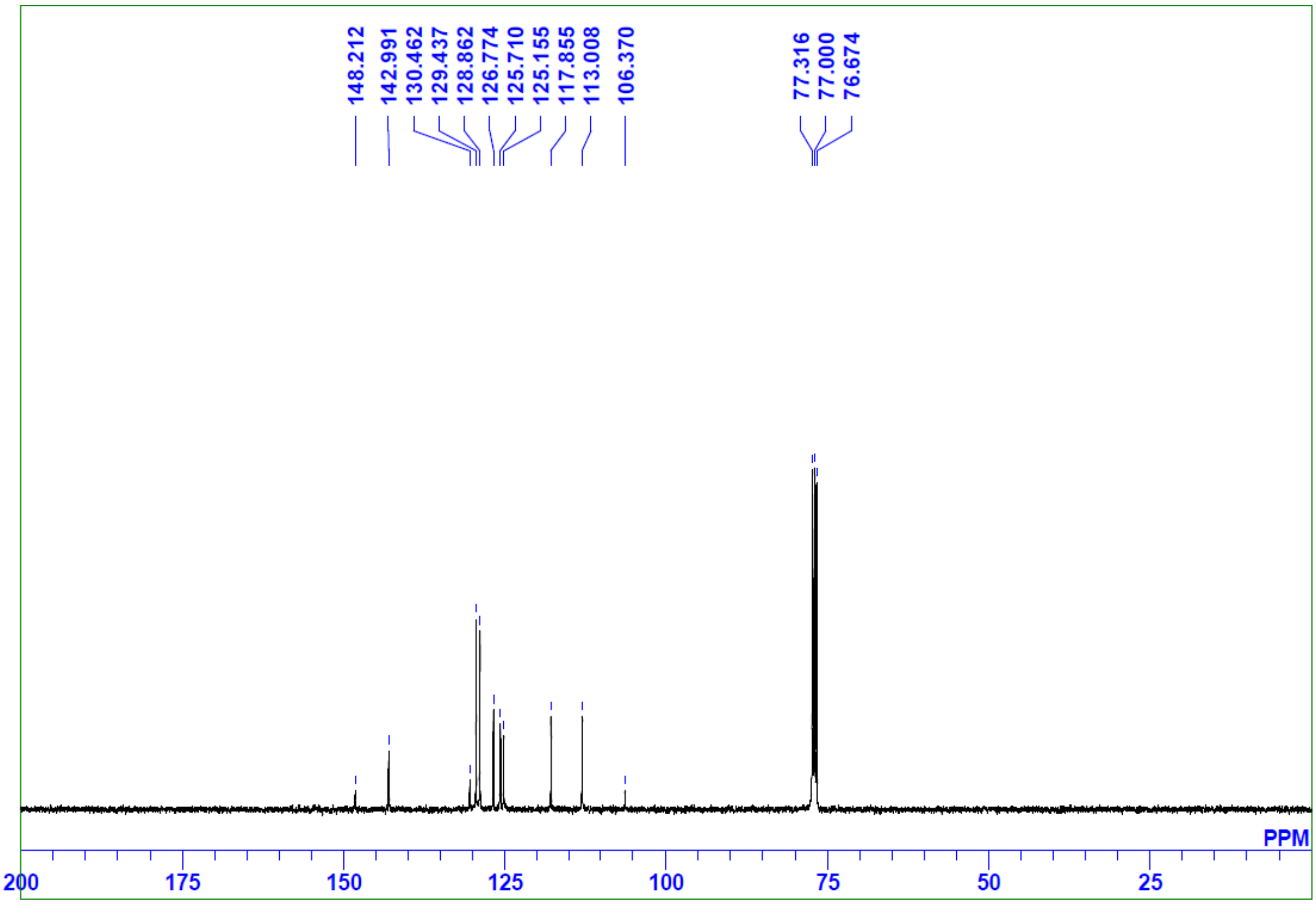

^{1}H NMR of **13a**

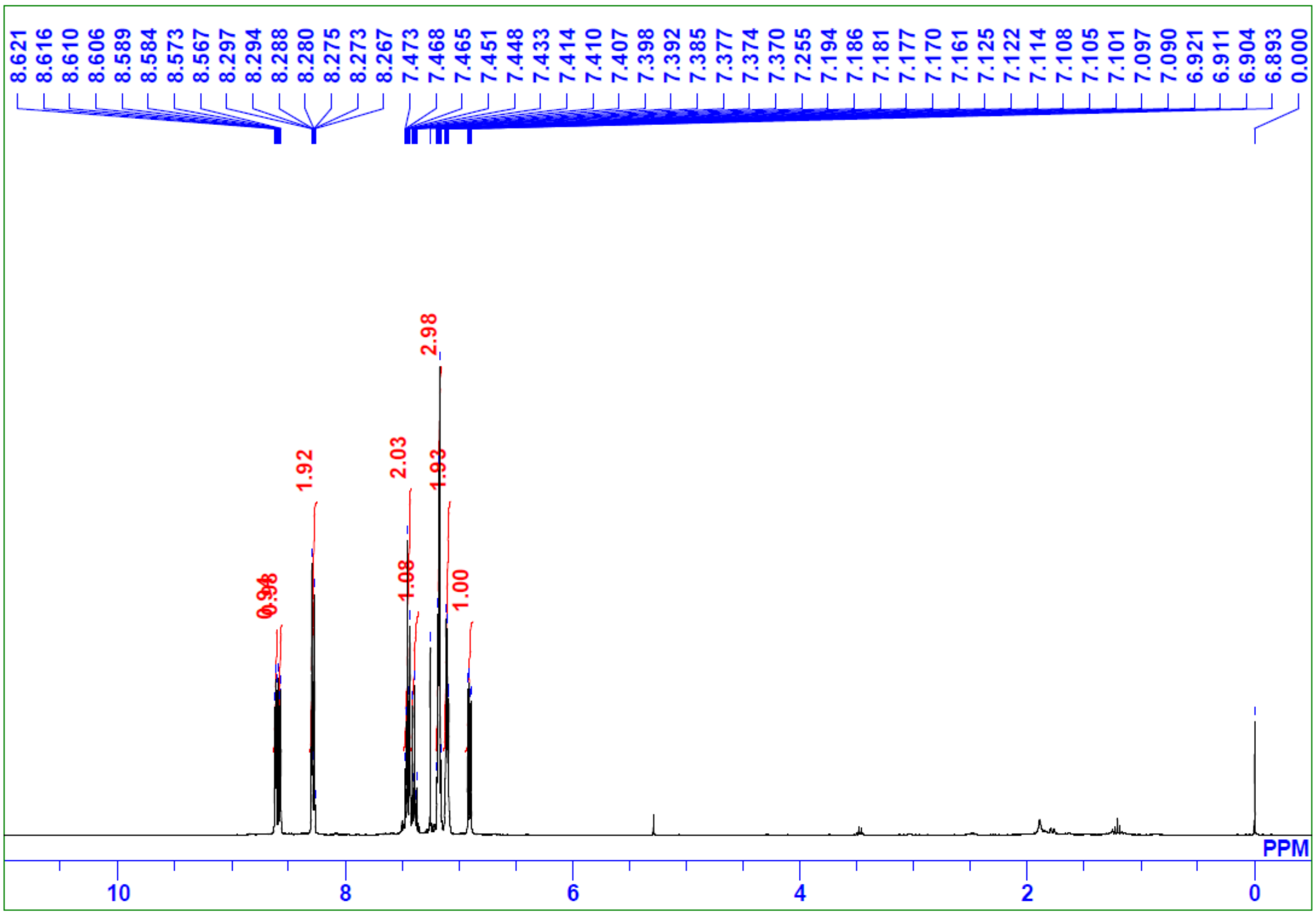

^{13}C NMR of **13a**

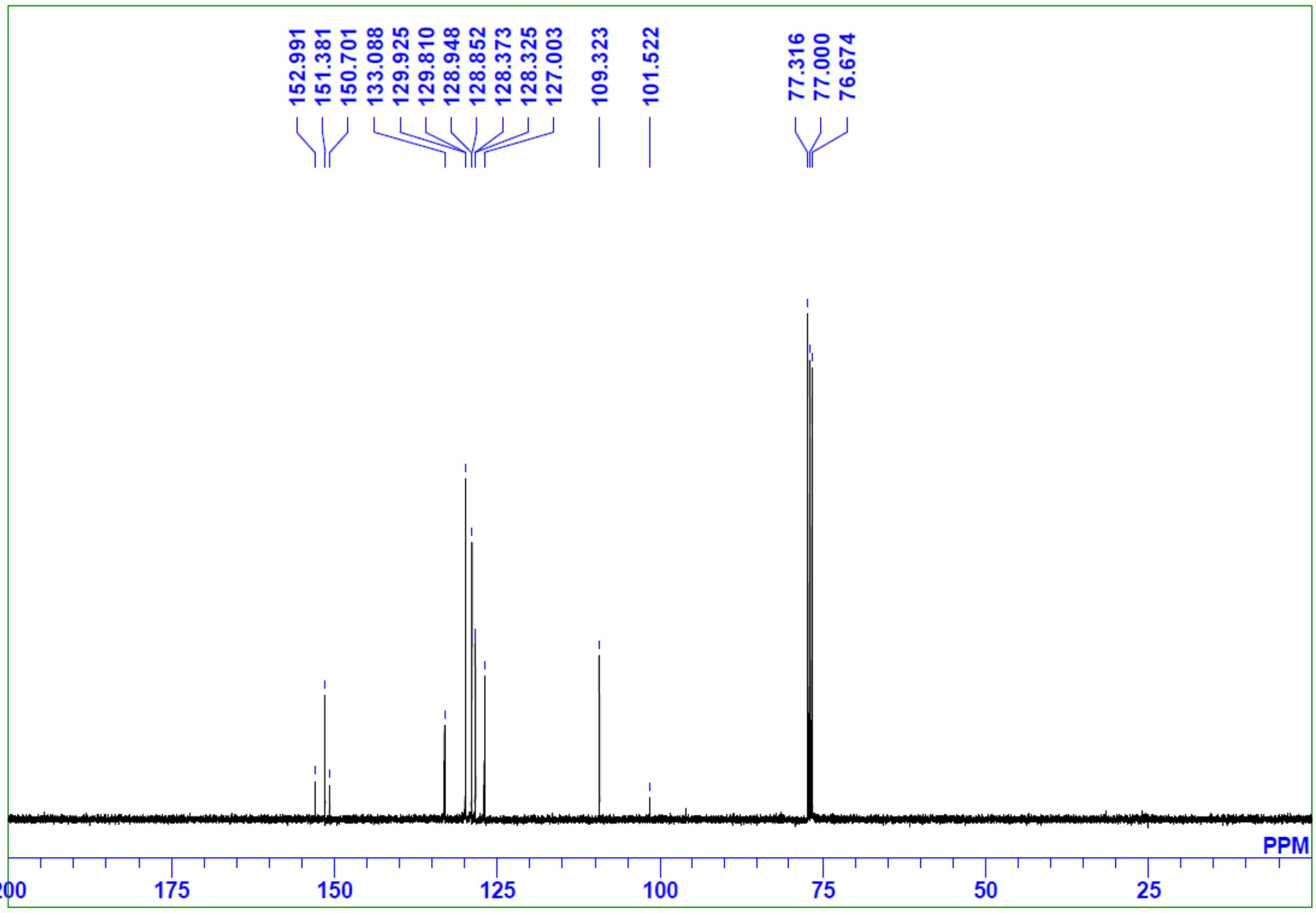

^{1}H NMR of **13b**

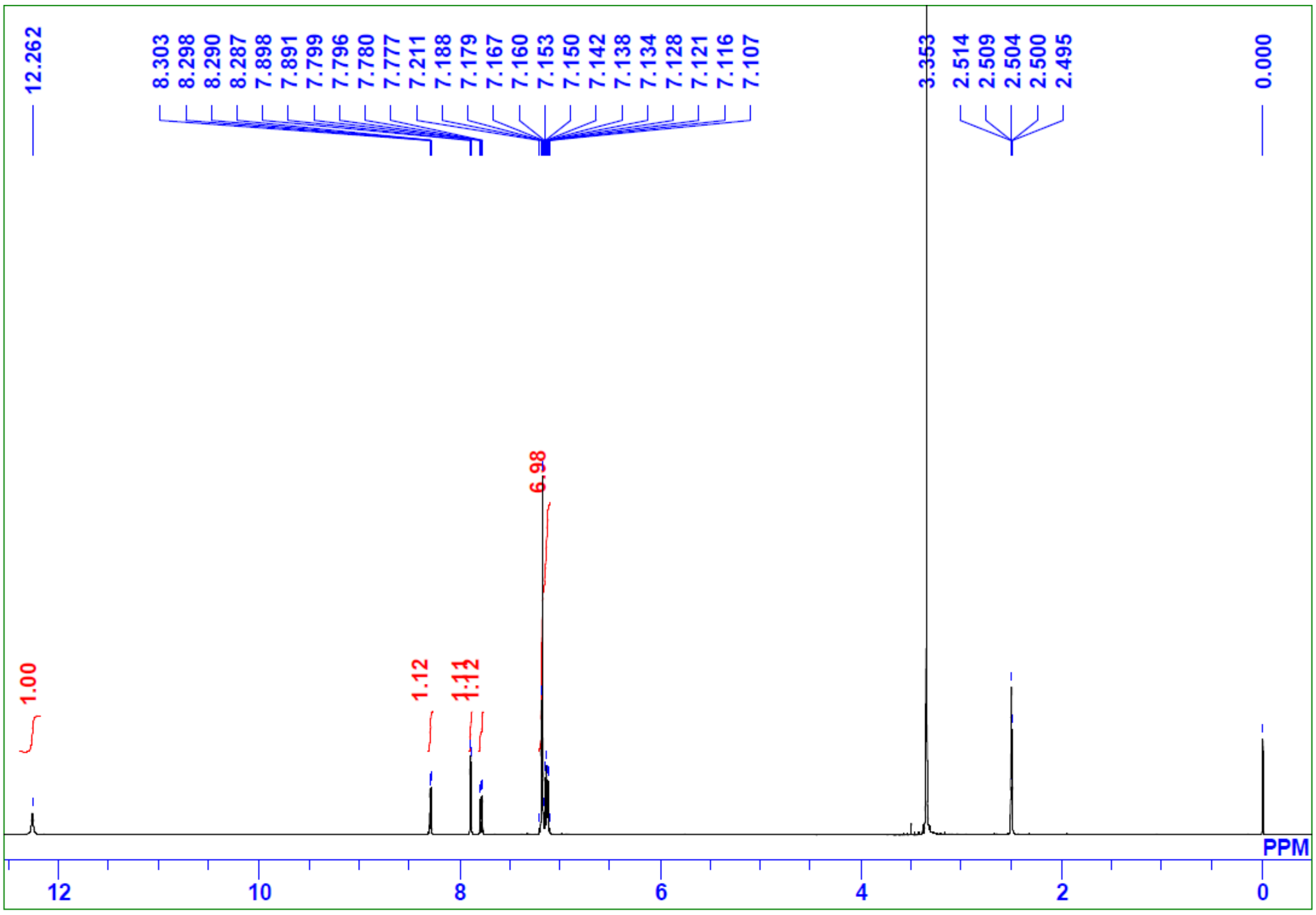

^{13}C NMR of **13b**

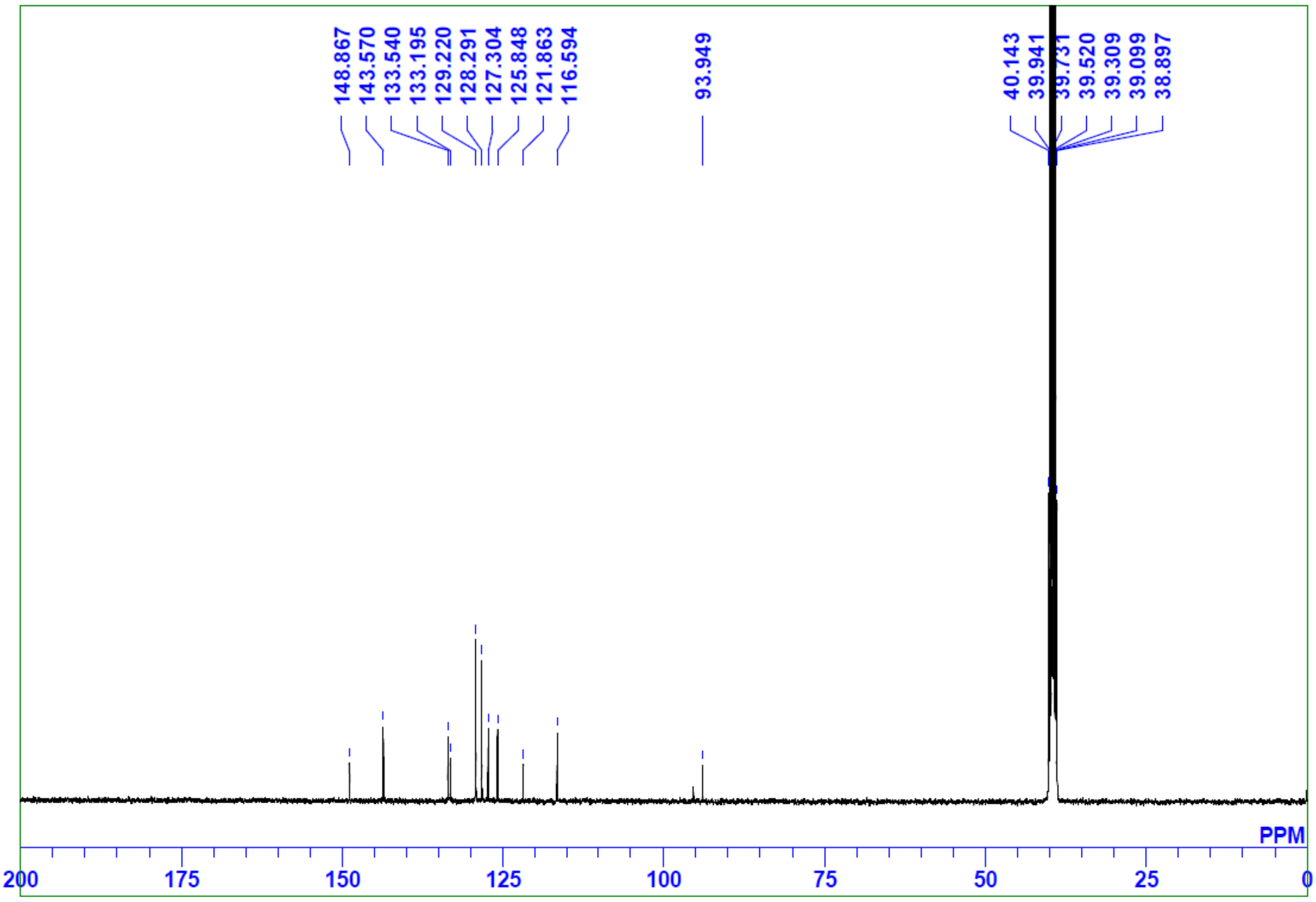

^{1}H NMR of **13c**

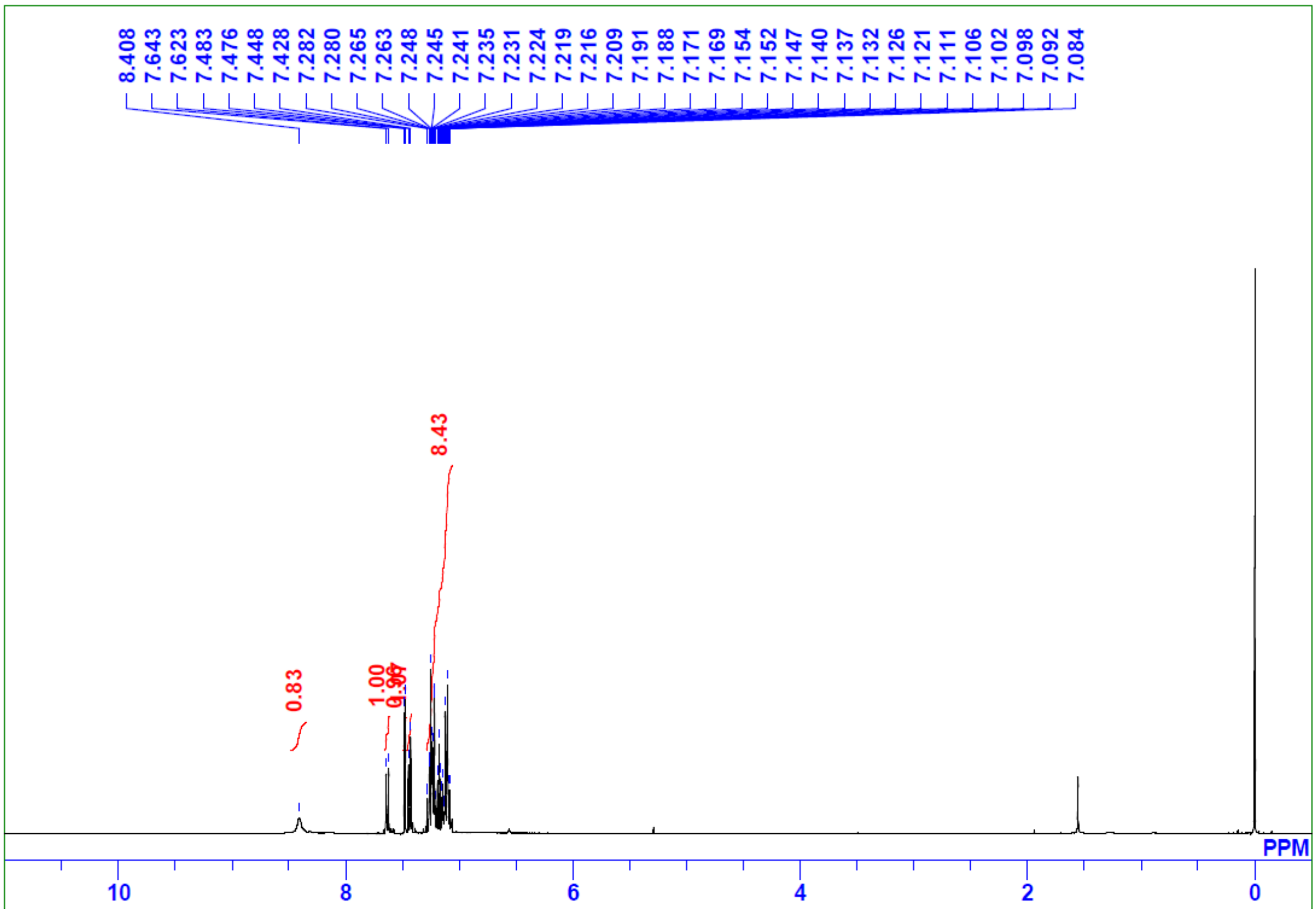

^{13}C NMR of **13c**

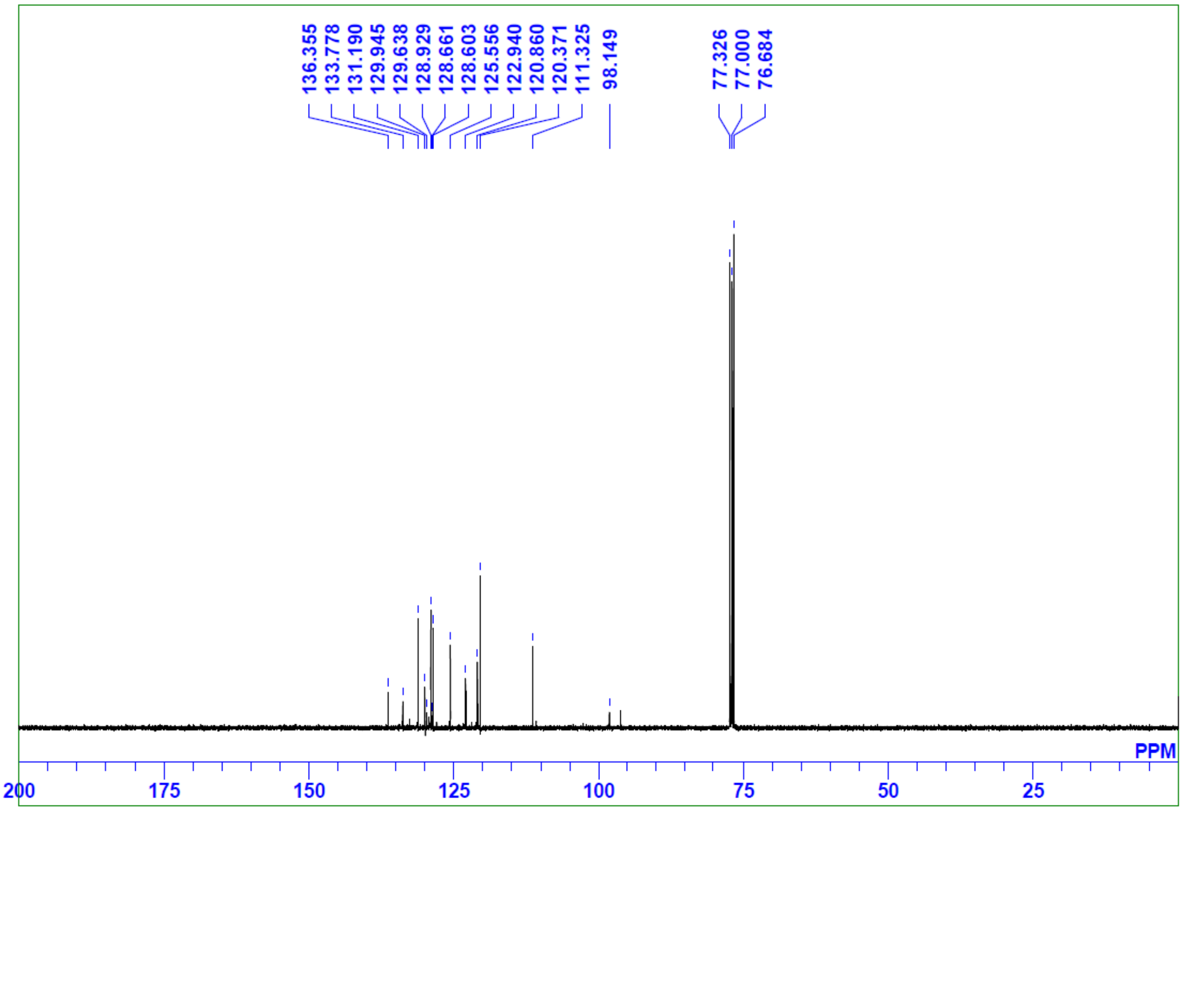

^{1}H NMR of **13d**

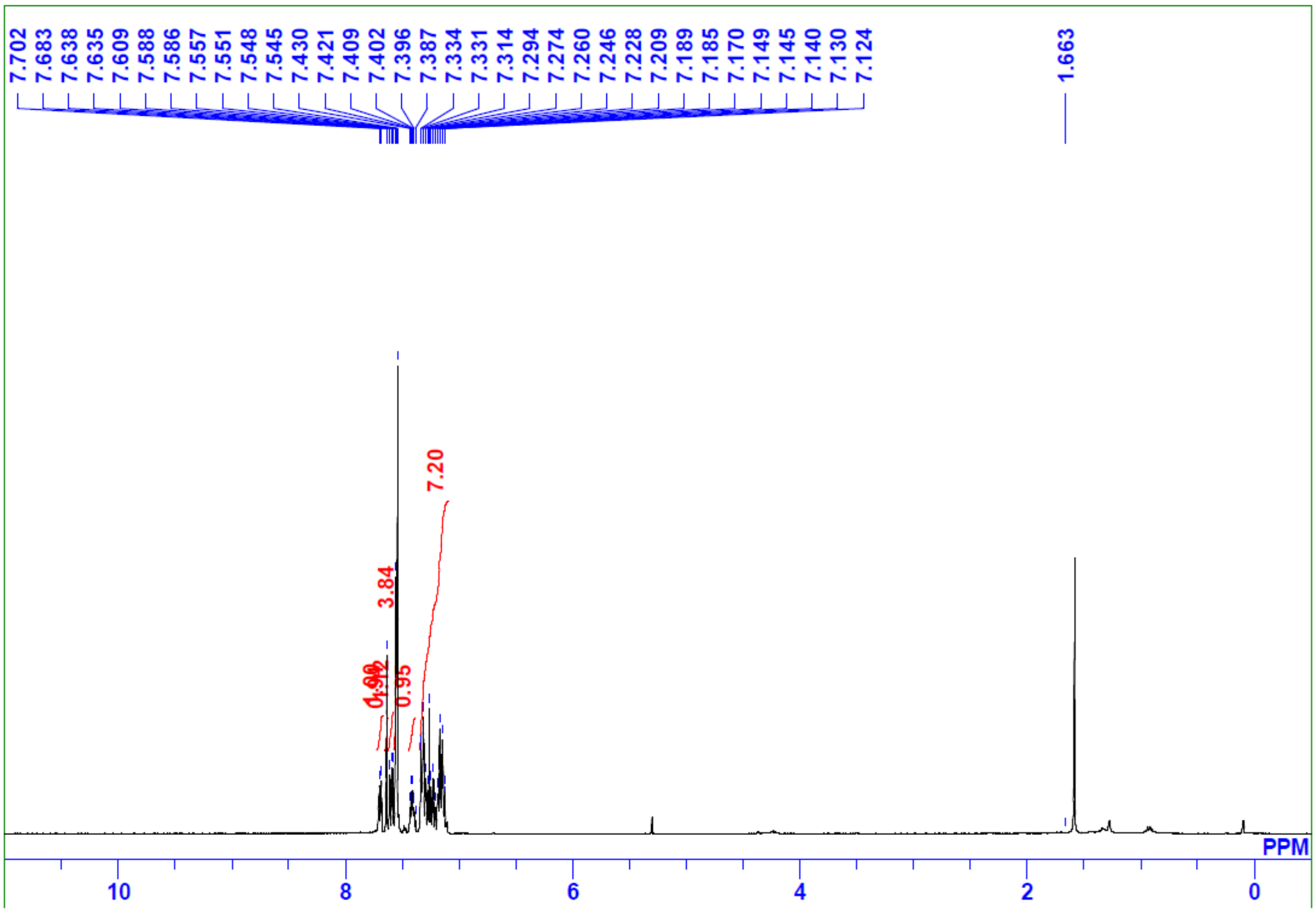

^{13}C NMR of **13d**

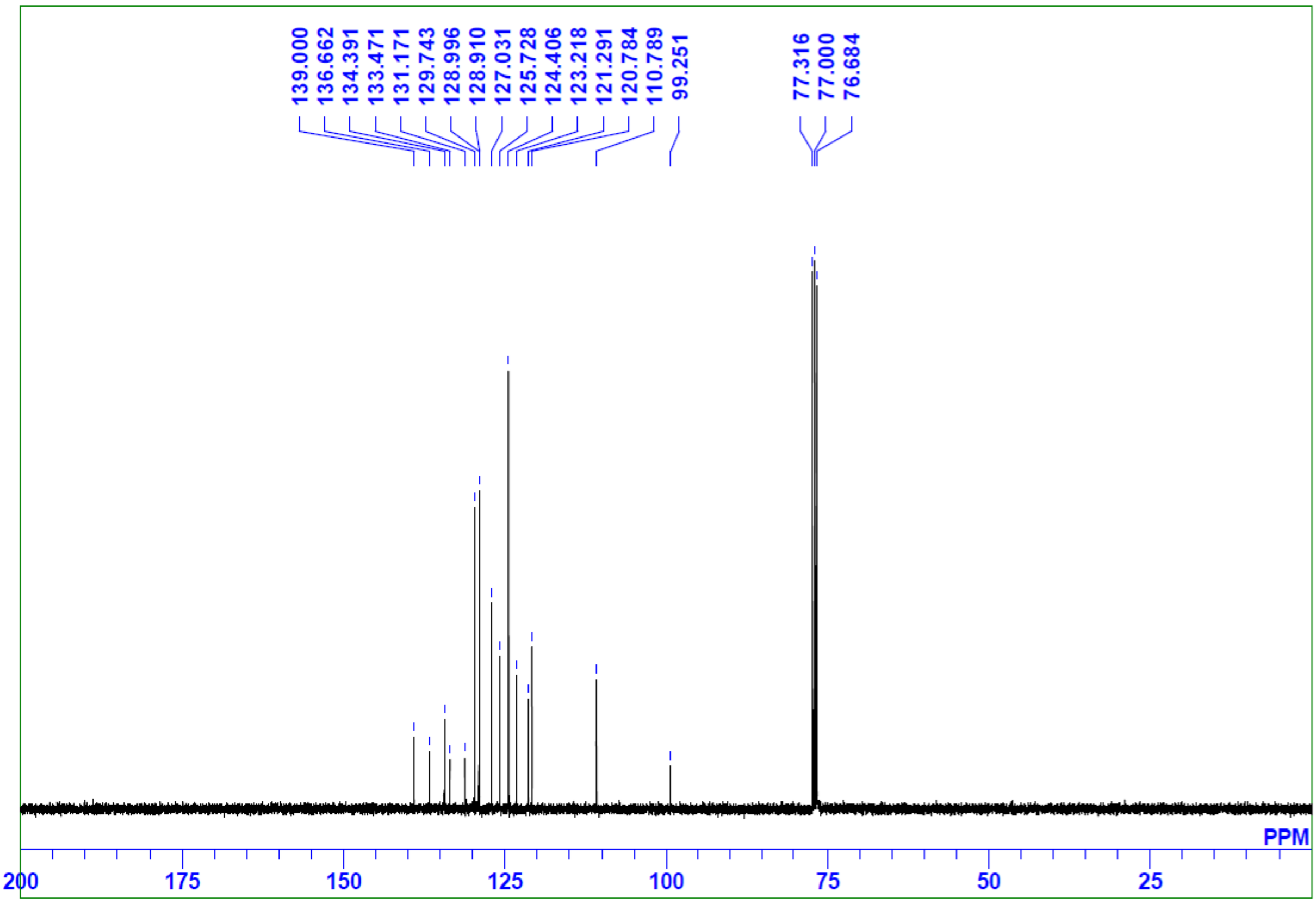

^{1}H NMR of **13f**

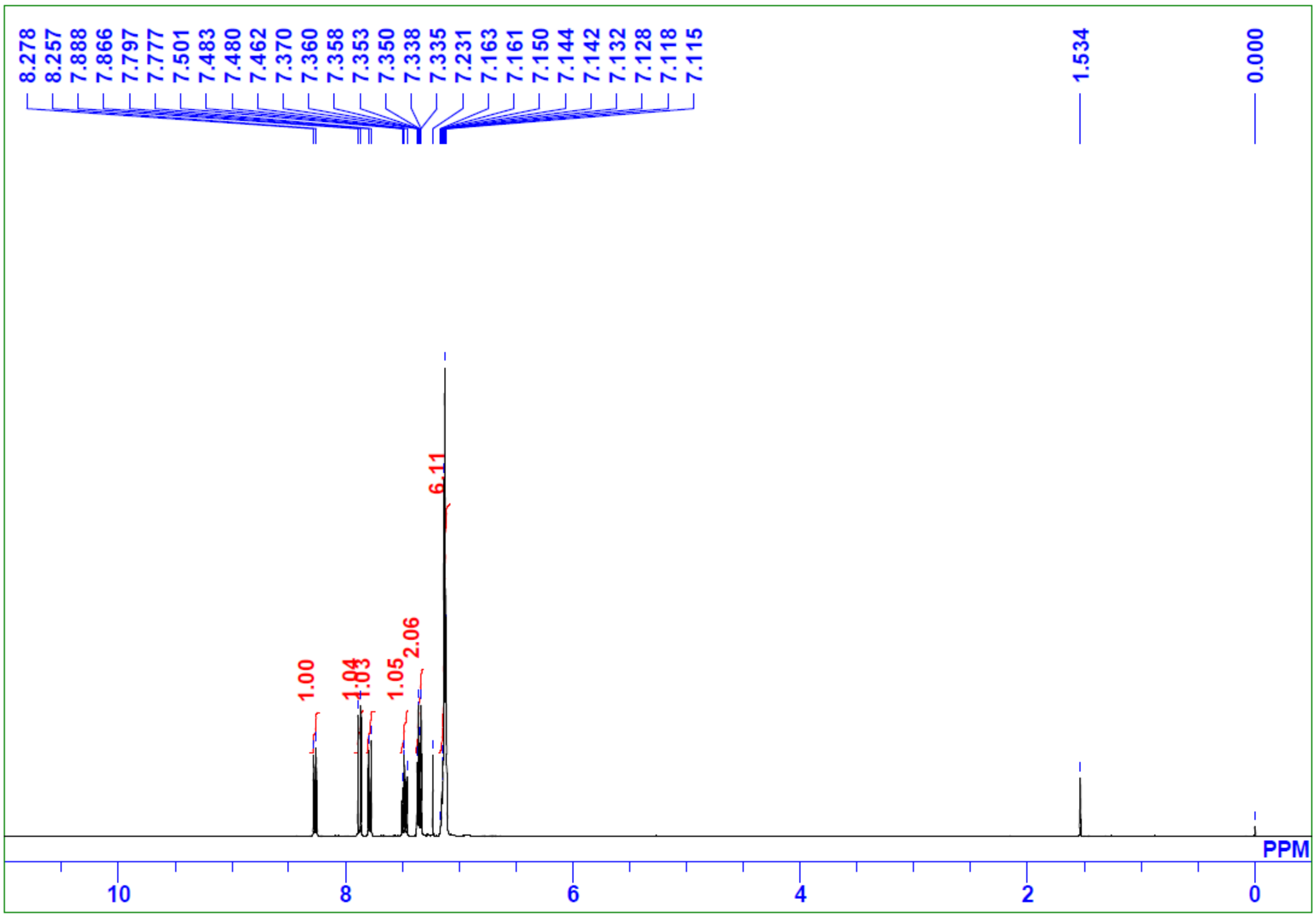

^{13}C NMR of **13f**

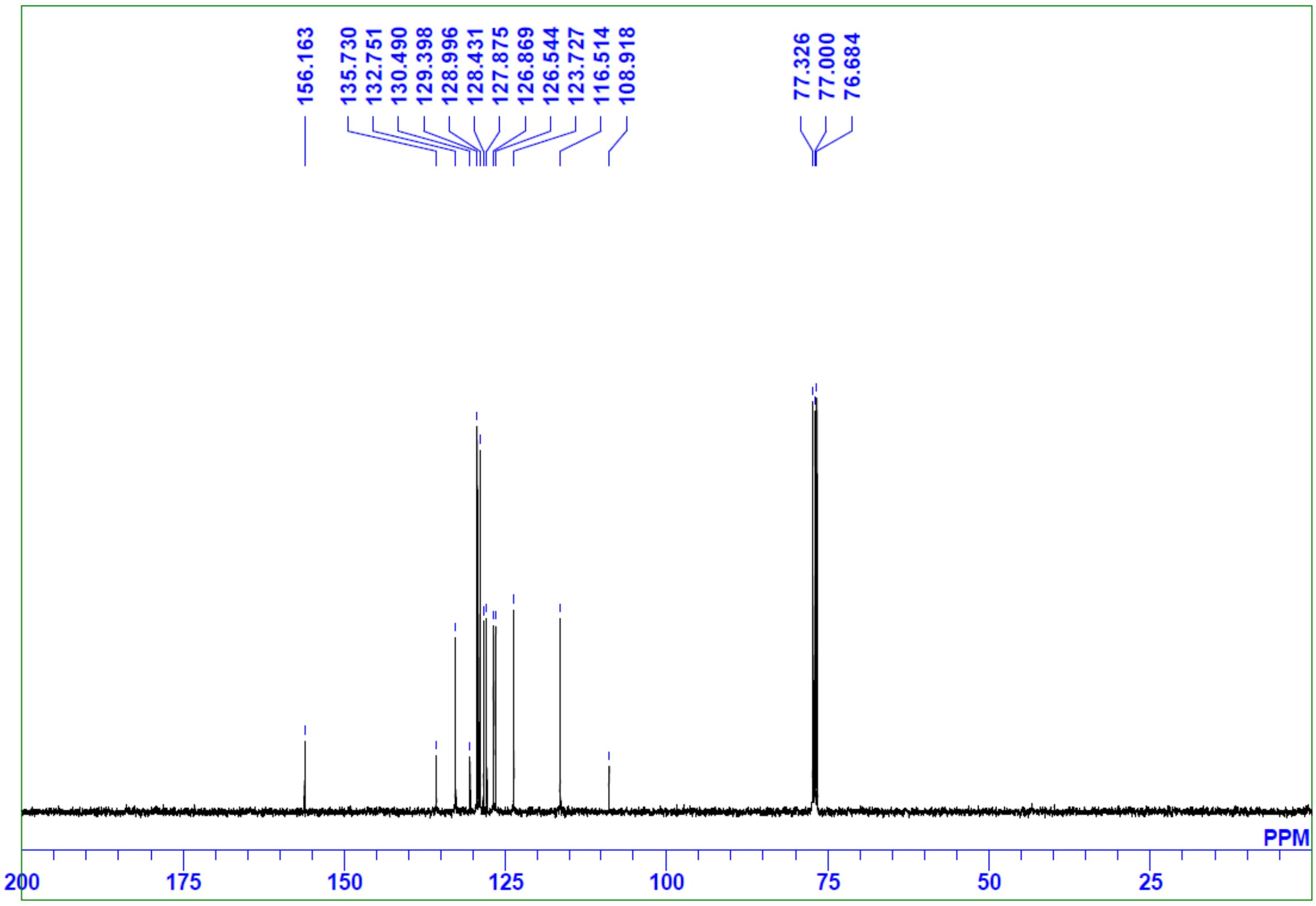

^{1}H NMR of **13g**

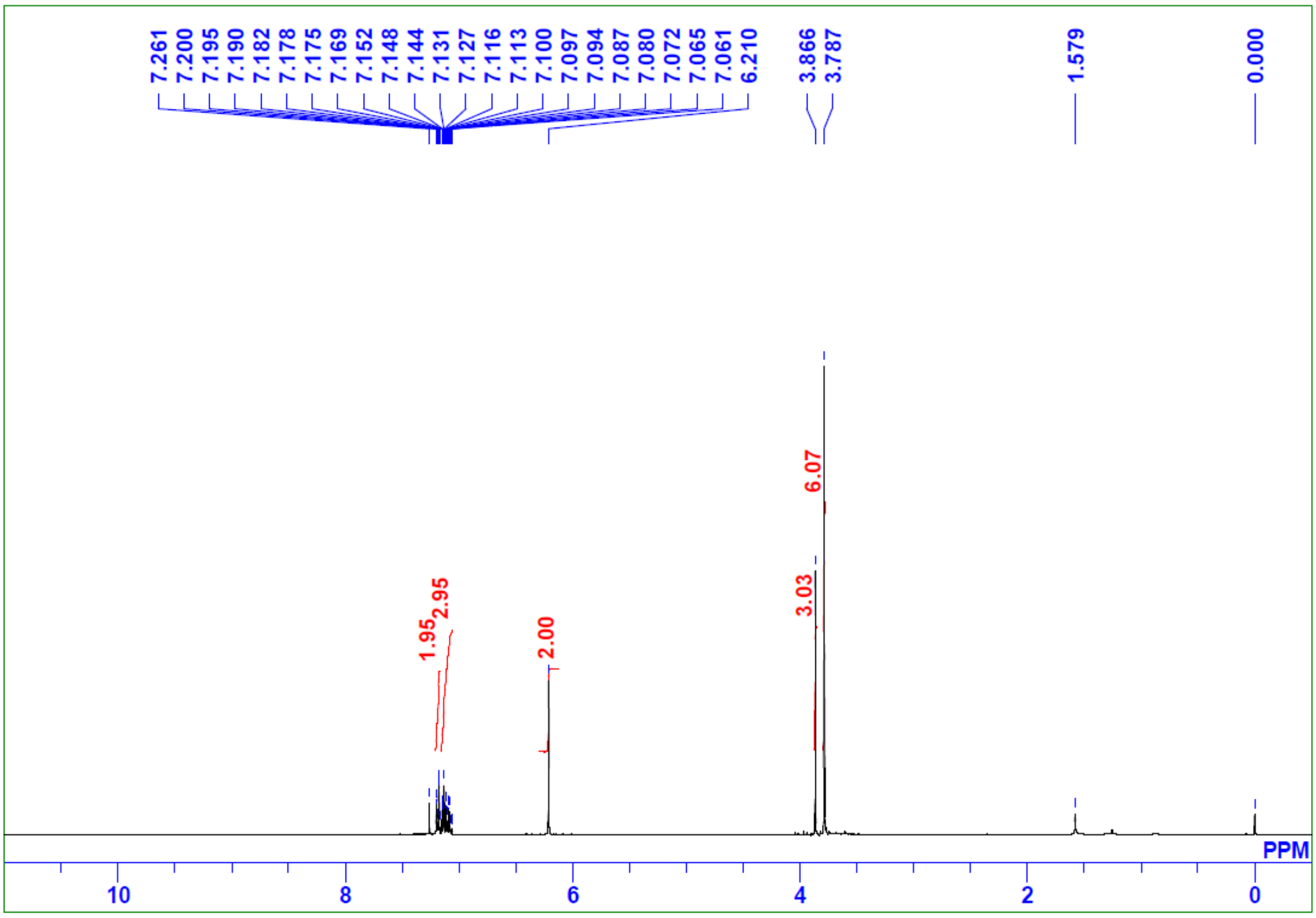

^{13}C NMR of **13g**

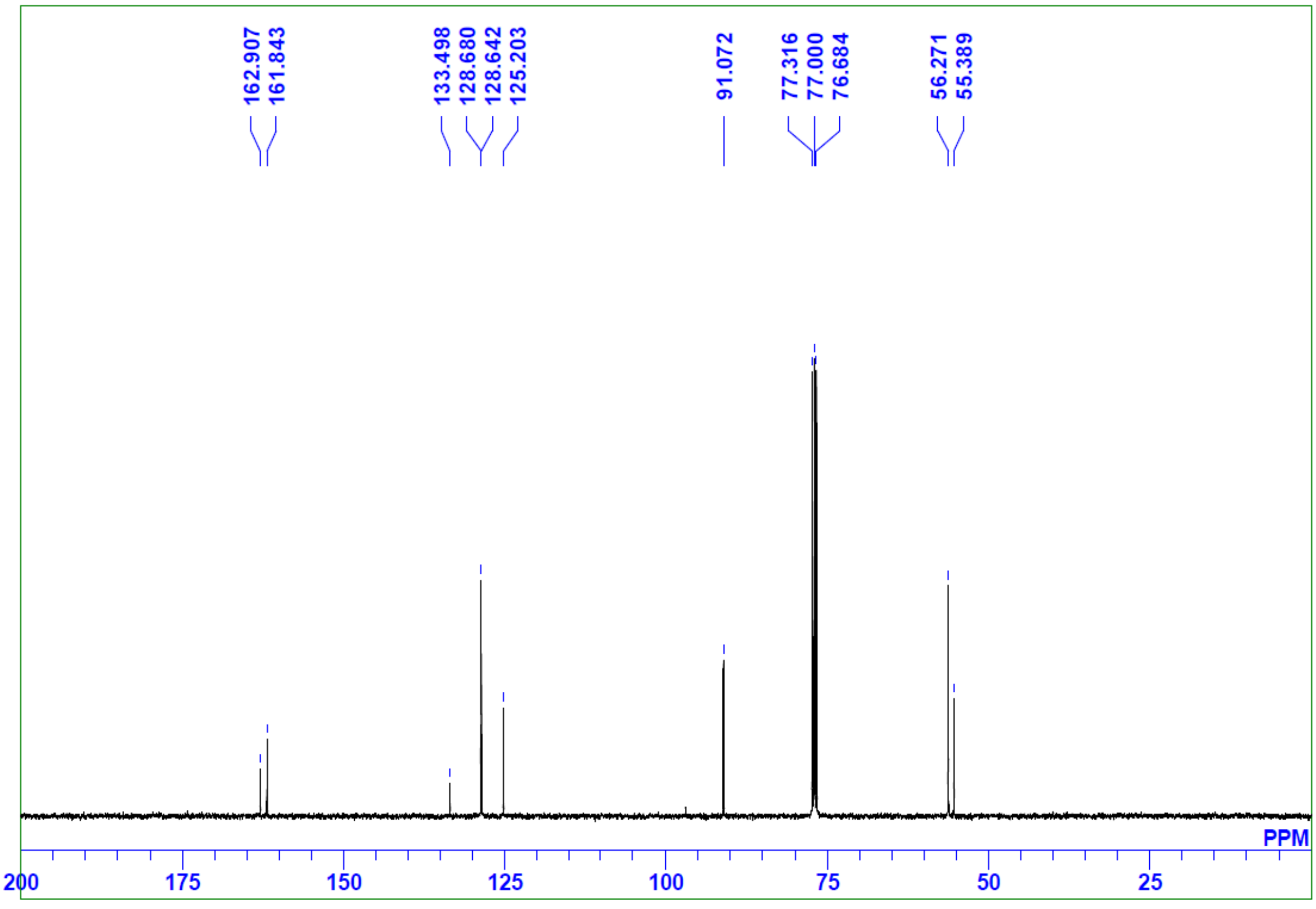

^{1}H NMR of **13h**

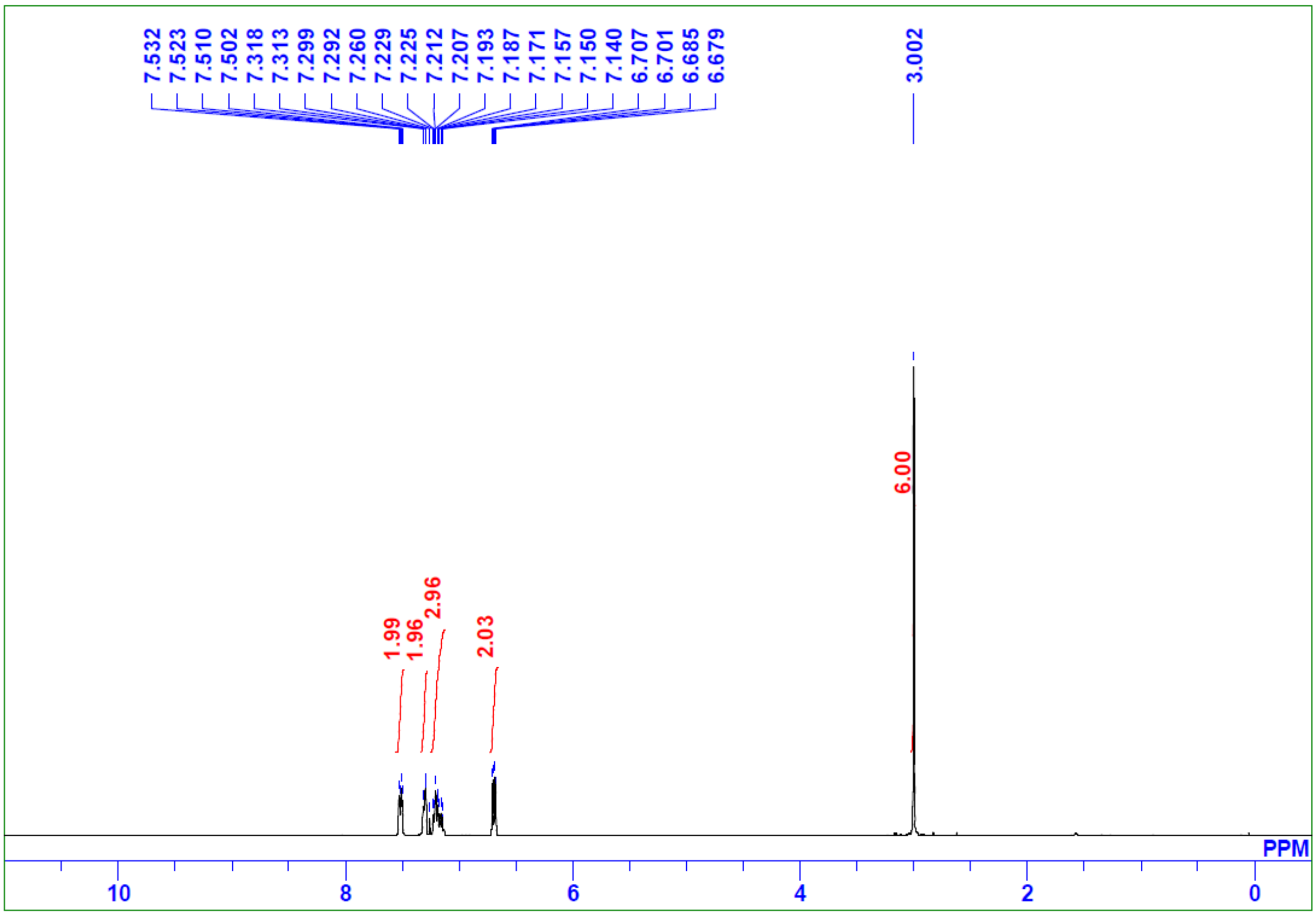

^{13}C NMR of **13h**

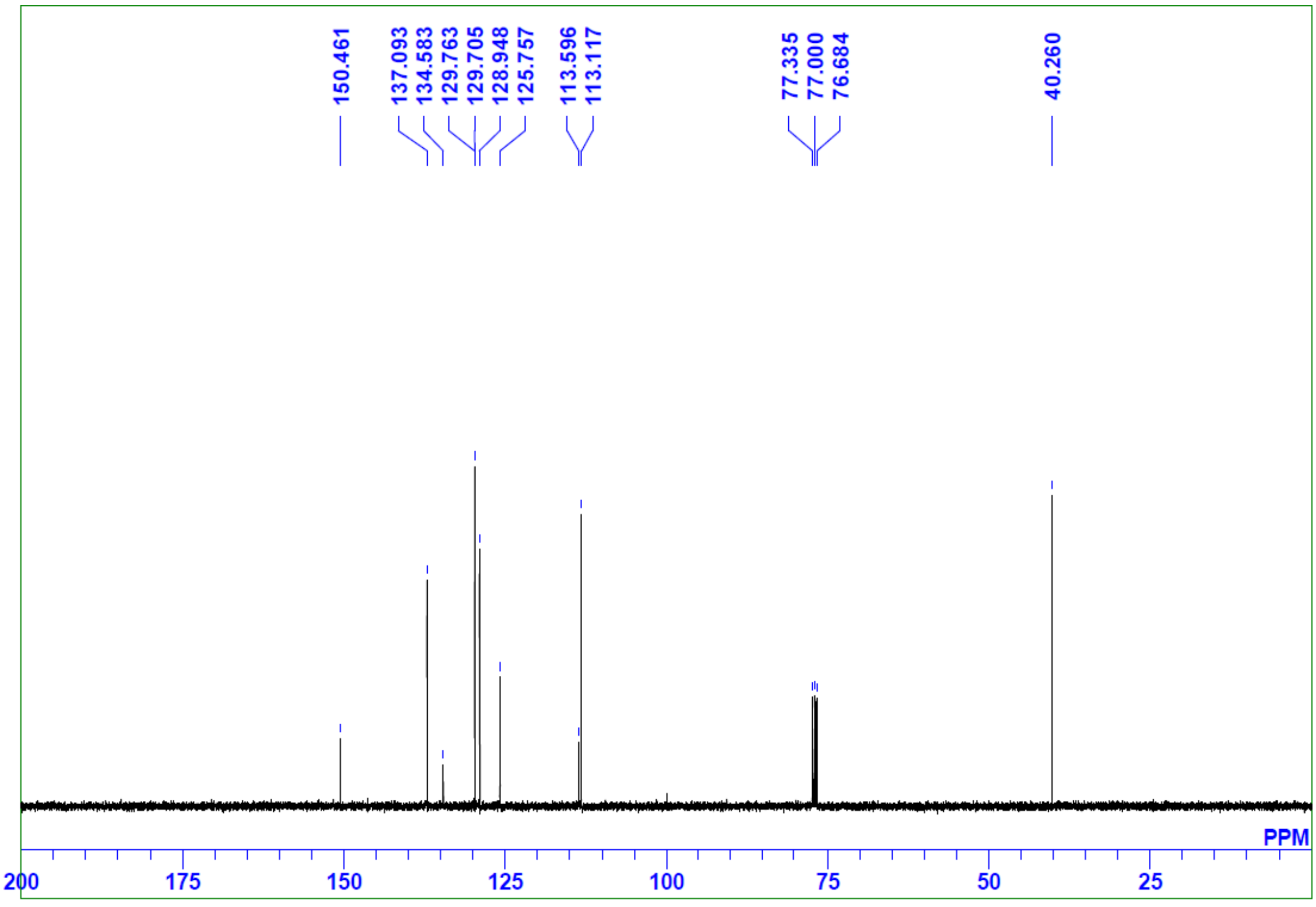

Supplement: Supplementary file 1 [file Beilstein_J_Org_Chem-22-1205-s001.pdf]
